# Huayu Jiedu Fang Protects Ovarian Function in Mouse with Endometriosis Iron Overload by Inhibiting Ferroptosis

**DOI:** 10.1155/2022/1406820

**Published:** 2022-08-30

**Authors:** Jie Ding, Qianqian Zhao, Zhihao Zhou, Wen Cheng, Shuai Sun, Zhexin NI, Chaoqin Yu

**Affiliations:** ^1^Department of Gynecology, The First Affiliated Hospital of Naval Military Medical University, Shanghai, China; ^2^Department of Pharmaceutical Sciences, Beijing Institute of Radiation Medicine, Beijing, China

## Abstract

Endometriosis (EM) is a common chronic inflammatory disease in women. Sampson's retrograde menstruation theory is the most widely accepted theory of EM pathogenesis. The periodic bleeding of ectopic lesions is an important pathological feature of this disease, and the occurrence and progression of EM are closely associated with the iron overload caused by ectopic lesions. However, animal models that simulate menstrual-blood reflux and hemorrhage from EM lesions are lacking. In this study, we performed intraperitoneal injection of endometrial fragments and periodic intraperitoneal blood injection to simulate the real cause and disease state of EM and successfully constructed a mouse model of EM iron overload. Our research found that the number, size, and degree of adhesion of EM lesions in the iron-overload model mouse were significantly higher than those in the model mouse. Moreover, the iron concentration in the abdominal fluid and ovary significantly increased, and the level of malondialdehyde (MDA) in the ovary increased. Conversely, GPX4, GSH, and other anti-ferroptosis-related proteins were downregulated, proving the occurrence of ferroptosis. Huayu Jiedu Fang (HYJDF) is an empirical prescription for EM treatment. This study combined animal experiments, UHPLC-QE-MS analysis, and network pharmacology to analyze whether HYJDF can inhibit ferroptosis to slow down the progression of EM and protect ovarian function. Based on the constructed iron-overload model, HYJDF can reduce the volume of EM lesions and the degree of adhesion, downregulate the total iron concentration in the peritoneal fluid and ovary, upregulate GPX4 expression and GSSG in the ovary, downregulate the level of MDA in the ovary, and promote the development of follicles. We further confirmed that HYJDF can inhibit the progression of EM disease and improve the ovarian function of the model mouse by inhibiting ferroptosis. Finally, through UHPLC-QE-MS and network pharmacology analysis, the natural compounds in HYJDF were identified and verified and the regulatory effect of HYJDF on the EM ferroptosis pathway through the IL-6/hepcidin pathway was preliminarily elucidated.

## 1. Introduction

Endometriosis (EM), an estrogen-dependent disease, is characterized by the growth of endometrial stromal and glandular cells outside the uterine mucosa and the myometrium [1]. EM often manifests as ovarian cysts, chronic pelvic pain, and infertility and affects 10%–15% of women of reproductive age [2]. Due to pain and infertility, EM greatly damages women's quality of life and psychological state and is also a major economic burden [3]. Nearly 40% of EM patients have infertility. The reasons for EM infertility are complex, ranging from anatomical distortion caused by adhesions and fibrosis to endocrine abnormalities and immune disorders, and then to insufficient ovarian reserve [4, 5]. The current treatment of EM-related infertility focuses on stimulating follicular development and ovulation or inhibiting the growth and development of EM lesions [5].

The pathogenesis of EM is not yet fully elucidated. Current theories include Sampson's retrograde menstruation theory, lymphatic dissemination, venous dissemination, and coelomic metaplasia theory [6]. Among them, Sampson's retrograde menstruation theory is the most widely accepted theory. During menstruation, the glandular epithelium and mesenchymal cells of the endometrium may reflux with menstrual blood, enter the pelvic cavity through the fallopian tube, and be planted in the ovary and adjacent pelvic peritoneum. The ectopic endometrium continues to grow and spread at the planting place, thereby forming EM [7]. Menstrual reflux is present in about 80%–90% of women, and because of the menstrual cycle, it is a problem that cannot be avoided for women of reproductive age. Additionally, an important pathological feature of EM is the periodic bleeding of ectopic lesions. Macrophages phagocytose the lysed erythrocytes and their degradation product hemoglobin. These are degraded to ferric ions by the action of heme oxygenase-1 (HO-1) [8]. Random retrograde menstruation and local bleeding from the lesions cause an increase in erythrocytes in the peritoneal tissue, ectopic lesions, and peritoneal fluid of EM patients, followed by iron aggregation and iron overload. Excess iron can generate large amounts of reactive oxygen species (ROS) via the Fenton reaction, causing lipid peroxidation, producing malondialdehyde (MDA) and 4-hydroxynonenal, and promoting the growth of ectopic lesions and the formation of adhesions [9]. Meanwhile, the ovaries are located in the pelvic and abdominal cavities, and the progression of EM disease must have an impact on ovarian function. In a previous study, our team found that the peritoneal-fluid iron levels of EM patients are elevated, follicular-fluid iron concentrations are abnormal, and follicular-fluid transferrin levels in stage III/IV EM infertility patients are significantly decreased, iron-ion levels are increased, and iron overload is present. Moreover, follicular fluid from stage III/IV EM infertility patients causes a significant increase in ROS levels in mouse oocytes and a significant decrease in oocyte maturation rate [10]. Accordingly, we propose the hypothesis that recurrent bleeding from pelvic and ovarian ectopic lesions leads to iron overload in the perivitelline environment and in the ovary itself, subsequently inducing the onset of ferroptosis, downregulating ovarian function, and affecting follicular development and oocyte quality.

However, with the exception of primate models, which are similar to humans in that they have periodic menstrual bleeding and focal bleeding, current EM models are simply endometrial tissue transplants. These models do not consider the periodic bleeding environment of EM focal lesions, and the animal models themselves do not have menstrual bleeding, differing from the clinical and physiological situation of EM [11]. Animal models for research on EM are also too costly to use for large-scale experiments. To better simulate the real pathology of human EM and determine whether iron overload affects ovarian function in EM disease, we constructed the first mouse model of iron overload in EM based on the original rodent model by periodically injecting blood intraperitoneally to simulate retrograde hemorrhage and periodic hemorrhage from the lesion, thereby further matching the actual disease state of EM.

Current treatment options for EM include pharmacological and surgical treatments, but all are insufficiently effective, have high side effects, and have a high recurrence rate. Additionally, all first-line EM treatment options inhibit ovarian function [12]. The ovarian reserve function of EM patients is related to the severity of EM. In the management of EM infertility, neither pharmacological treatment nor postsurgical pharmacological treatment improves the natural pregnancy rate in EM patients. The surgical treatment of ovarian EM can lead to ovarian damage during the removal of EM tissue, leading to the deterioration of ovarian reserve function and thus exacerbating EM infertility. The abovementioned problems render complementary and alternative medicine a more valuable option for EM treatment. Chinese medicine has been used in China for thousands of years and has the advantage of stable efficacy and low side effects. Meanwhile, Chinese medicine is individualized and systemic in nature, meeting the need for individualized treatment of EM patients [13, 14]. Therefore, the search for safe and effective Chinese medicine therapies for EM treatment is highly significant.

Based on the successful model construction, we investigated the effects of Huayu Jiedu decoction (HYJDF) on EM and ovarian function. HYJDF is an empirical formula for the clinical treatment of EM. It is primarily composed of *Epimedium brevicornum Maxim (EbM, Chinese name”yin yang huo*),” *Sargentodoxae caulis (SCC, Chinese name“da xue teng*),” *Patrinia scabiosaefolia (PS, Chinese name“pai jiang cao*),” and *Typha angustifolia* L*(TaL, Chinese name“pu huang*).” Through the treatment of 58 clinical cases, our team found that the total clinical efficacy of HYJDF was 82.8%. The patients' dysmenorrhea and pelvic pain scores were significantly reduced after treatment *P* < 0.01(*P* < 0.01), and the ovarian endometrioid cysts were significantly reduced after treatment (*P* < 0.05) (clinical observation on stasis-transforming toxin-resolving formula for 58 cases of endometriosis). In a mouse model, we found that HYJFD could improve the intestinal environment of endometriosis mice, reduce the level of LPS *in vivo*, and reduce the fibrosis of ectopic lesions (Huayu Jiedu prescription alleviates gut microbiota and fecal metabolites in mouse with endometriosis). Meanwhile, we found that HYJDF significantly increased the pregnancy rate in EM patients and model mice, reduced the level of oxidative stress in oocytes after follicular-fluid treatment, and increased the oocyte maturation rate in patients with EM infertility [10,15]. However, whether HYJDF can treat EM and promote follicular development by regulating iron overload and inhibiting ferroptosis remains an open question. Which are ferroptosis-related targets? Accordingly, we used ultra-high-performance liquid chromatography (UHPLC), Q-Exactive (QE), mass spectrometry (MS) analysis, and network pharmacology to analyze and designed the whole experiment to test and verify.

**Figure 1:**
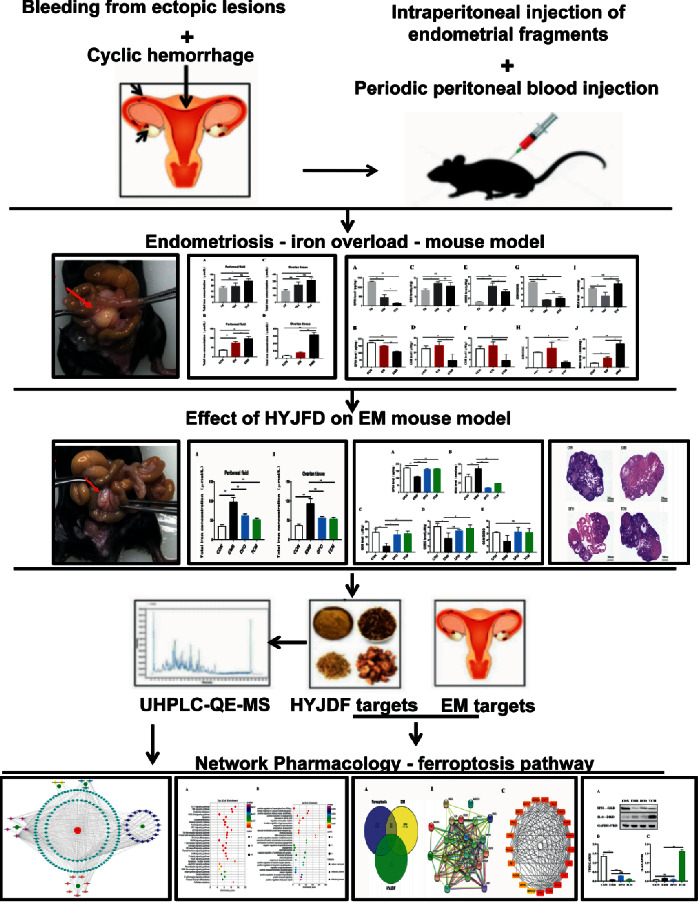
| The flow chart of iron-overload model mouse construction and HYJDF treatment of EM. First, an iron-overload model of EM mouse was constructed based on periodic intraperitoneal blood administration to demonstrate that ferroptosis plays a role in EM. In the second step, HYJDF was shown to inhibit ferroptosis to treat EM and improve ovarian function. Finally, plasmapheresis and network pharmacology were applied to investigate the core components in HYJDF and the specific mechanism of ferroptosis inhibition.

In the present study, we constructed the first iron-overload model for EM mouse, an animal model that closely resembled the pathophysiological condition of EM. Using MS, we identified the active chemotactic components in HYJDF. Combined with the network pharmacology analysis, we confirmed the relevance of HYJDF to the ferroptosis pathway and conducted preliminary studies on molecules related to the ferroptosis pathway. The overall study idea is shown in Figure 1.

## 2. Materials and Methods

### 2.1. Iron-Overload Model-Mouse Construction and Experimental Validation of HYJDF on EM *In Vivo*

#### 2.1.1. Animals

Female C57BL/6J mice (20–22 g) were purchased from Beijing Weitong Lihua Experimental Animal Technology Co., Ltd. The mice were reared in a specific pathogen-free environment. All experiments were performed in strict accordance with the guidelines of the Experimental Animal Center of Shanghai Changhai Hospital, and the principles of animal protection, animal welfare, and ethics were observed.

#### 2.1.2. Establishment of the Mouse EM Model

EM model mice were prepared according to a previously published study [16]. Every C57 mouse was injected with 0.2 mL of estradiol solution subcutaneously on the back of the neck three times at a frequency of one injection per 4 days. Four days after the last injection, the donor mice were executed. The uterus and adipose tissue were removed and washed in precooled PBS, after which the uterus was cut open longitudinally with ophthalmic scissors. The endometrial layer was peeled off, and the endometrial tissue and adipose tissue were quickly cut into endometrial fragments ≤1 mm^3^ in volume and prepared into the corresponding suspension. After disinfection of the abdominal skin of the recipient mouse, 0.5 cm above the urethral opening in the lower abdomen was used as the entry point for injecting 0.2 mL of endometrial fragments into the peritoneal cavity. The same volume of fat suspension was injected into the sham-operated (CON) group. The entire process from removal of the uterus to injection into the peritoneal cavity of the recipient mouse was completed within 5 min. All recipient mice were given three subcutaneous injections of estradiol after intraperitoneal implantation. A batch of the completely blank mice (20–22 g) was set aside, and then blood was removed from their eyes and they were executed. The fresh blood was stored in an ice box for intraperitoneal injection and was used within 30 min. All of the above operations were performed on an ultraclean table.

#### 2.1.3. Animal Grouping: Iron-Overload Model Mouse

**Figure 2:**
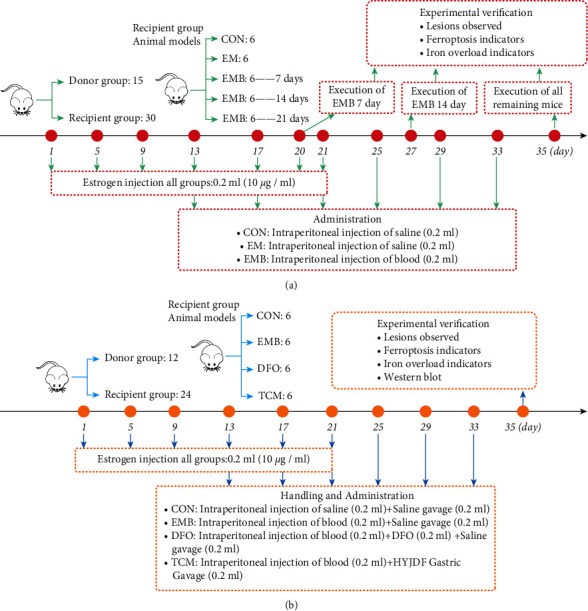
|The two stages of animal treatment and grouping. (a) The experimental procedures of the iron-overload mouse model (b) The experimental procedures of in the *in vivo* EM mouse model.

The experimental procedure of iron-overload model mouse construction is shown in Figure 2(a). A total of 45 mice were randomly divided into 15 donors and 30 recipient mice in a ratio of 1 : 2. A total of 24 recipient mice were used to prepare the EM model mouse, and 6 mice were used to prepare the CON group. The EM model mice were randomly divided into 18 mice in the blood injection group (EMB group) and 6 mice in the nonblood injection group (EM group). Six mice in the 7-day group, six mice in the 14-day group, and six mice in the 21-day group were then set up in the EMB group. (1) For the CON group, saline (0.2 mL/animal) was injected intraperitoneally from the day of implantation once every 4 days for 21 days. (2) For the EM group, saline (0.2 mL/each) was injected intraperitoneally from the day of implantation, and the duration of injection was 21 days. (3) For the EM group, saline (0.2 mL/animal) was injected intraperitoneally from the day of implantation once every 4 days for 21 days. (4) For the EMB group, whole blood (0.2 mL/animal) was injected intraperitoneally from the day of implantation once every 4 days for 7, 14, and 21 days, respectively.

### 2.2. Animal Grouping and Drug Administration: HYJDF Intervention

The experimental procedure of *in vivo* EM mouse model is shown in Figure 2(b). The 36 mice were randomly divided into 12 donors and 24 recipient mice in a ratio of 1 : 2. A total of 18 recipient mice were used to prepare the EM model, and 6 mice were used to prepare the CON model. (1) Six EM mice were randomly divided into six mice in the blood injection group (EMB group), six mice in the iron chelator deferoxamine (DFO) group, and six mice in the Chinese medicine group (TCM group), (0.2 mL/each) every 4 days until the 21^st^ day of implantation. (2) For the EMB group, whole blood was injected intraperitoneally (0.2 mL/animal) from the day of implantation once every 4 days until the 21^st^ day of implantation. At the same time, 0.2 mL of saline gavage was administered daily. (3) For the DFO group, whole blood (0.2 mL/each) was injected intraperitoneally from the day of implantation followed by DFO (0.2 mL/each) every 4 days until day 21 of implantation; at the same time, 0.2 mL of saline gavage was administered daily. (4) For the TCM group, whole blood (0.2 mL/each) was injected intraperitoneally from the day of implantation, and 1 injection was administered every 4 days until day 21 of implantation. At the same time, 0.2 mL of HYJDF gavage was administered daily.

### 2.3. Preparation of HYJDF and Experimental Reagents

The decoction was converted to the equivalent daily dose of 43.68 g/kg for mice according to the conversion table. The equivalent daily dose for the mouse was obtained as 43.68 g/kg after conversion according to the drug equivalent-dose conversion table. The decoction of the herbal medicine was concentrated to 4.368 g/mL of the herbal stock solution by spin-concentration method, and the stock solution was diluted five times with distilled water before use in consideration of drug toxicity. Estradiol benzoate (MedChemExpress, No. HY-B1192) was purchased from MCE, and 0.5 mg of *β*-estradiol benzoate powder was dissolved in 50 mL of experimental sesame oil to form a solution of estradiol at a concentration of 10 *μ*g/mL. The resulting solution was stored at 4°C in a refrigerator protected from light. After dissolving 1 mg of DFO powder in 50 mL of saline to make 20 *μ*g/mL DFO, it was stored away from light in a refrigerator at 4°C.

### 2.4. Sample Collection and Lesions Assessment

Mice were anesthetized using sodium pentobarbital (50 mg/kg; intraperitoneal) and executed. After disinfecting with 75% alcohol, 2 mL of precooled saline was injected into the peritoneal cavity, which was gently massaged. Peritoneal lavage fluid was then collected. After the abdominal cavity was opened, the mice were scored for the degree of abdominal adhesions using Blauer's adhesion scoring system. The scoring criteria were as follows: 0, no adhesions; 1, slight membranous adhesions in the pelvis; 2, dense adhesions, usually with the uterine horns attached to the intestinal canal and bladder; 3, denser and extensive adhesions, with uterine horns attached to the intestinal canal and bladder, with some mobility of the uterus; 4, severe adhesions, with uterine horns attached to the intestinal canal and bladder, with the uterus immobilized [17]. The final adhesion score was obtained by averaging the scores of two experimentalists independently. The number of ectopic lesions was counted, and the length, width, and height of the lesions were measured with Vernier calipers after removal. The volume of the implant was calculated according to formula *V* (mm^3^) = *π*/6 × length (mm) × width (mm) × height (mm) and recorded [18]. Finally, ovarian tissue was collected and stored at −80°C.

### 2.5. Determination of Total Iron Concentration in Peritoneal Fluid and Ovarian Tissue

This assay was performed according to the instructions of an Iron Assay Kit (Nanjing Jiancheng Institute of Biological Engineering, A039-1-1).

(1) Determination of total iron concentration in lavage fluid: the collected lavage fluid was centrifuged at 1300 rpm/min for 5 min at 4°C, and the supernatant was collected and divided into three assay tubes (blank, standard, and assay). Each was added with the iron chromogenic agent, and then double distilled water, iron standard application solution, and the sample to be tested were added to each tube. After mixing, the supernatant was centrifuged at 3500 rpm for 10 min after cooling. The wavelength was 520 nm. The absorbance OD value of each tube was measured and the corresponding total iron concentration was calculated. (2) Determination of total iron concentration in ovarian tissue: The removed ovaries were ground and crushed to form a tissue homogenate, which was divided into three assay tubes (blank, standard, and assay). The iron chromogenic agent was added to all three tubes, and then double distilled water, iron standard application solution, and samples to be tested were added. After mixing, the tubes were boiled for 5 min, cooled, and centrifuged at 3500 rpm for 10 min. The supernatant was collected at 0.5 cm optical diameter and 520 nm wavelength and zeroed with double distilled water. The absorbance OD value of each tube was measured, and the corresponding total iron concentration was calculated.

### 2.6. Determination of GPX4 in Ovarian Tissue

This assay was performed according to the instructions of a mouse phospholipid hydroperoxide glutathione peroxidase mitochondrial (GPX4) ELISA kit (Cusabio, CSB-EL009869MO). To prepare ovarian-tissue homogenate samples, we used a standard kit and added 100 *μ*L of standards and samples per well. The samples were covered with an adhesive strip and incubated at 37°C for 2 h. Subsequently, 100 *μ*L of biotin antibody (1×) was added per well, and it was covered with an adhesive strip and incubated at 37°C for 1 h. We aspirated and washed each well and repeated this process twice for a total of three washes. After the last wash, the remaining wash solution was removed. After adding 100 *μ*L of enzyme affinity (1×) to each well, the microtiter plate was covered with a new adhesive strip and incubated at 37°C for 1 h. The washing procedure was repeated, and 90 *μ*L of TMB substrate was added to each well, which was incubated at 37°C for 15–30 min and protected from light. After adding 50 *μ*L of termination solution, the plate was gently tapped to ensure adequate agitation. The OD value of each well was measured within 5 min using an enzyme marker set to 450 nm.

### 2.7. Determination of GSH/GSSG in Ovarian Tissue

This assay was performed according to the instructions of a total Glutathione-Oxidized Glutathione (T-GSH/GSSG) Test Kit (Nanjing Jiancheng Institute of Biological Engineering, A061-1-1). The standard tube was filled with GSH and GSSG standards, and the assay tube was filled with the sample to be tested. The rest of the treatment was the same for both tubes.

### 2.8. Detection of MDA in Ovarian Tissue

This assay was performed according to the instructions of a cellular MDA assay kit (Nanjing Jiancheng Institute of Biological Engineering, A003-4-1). Ovarian tissues were homogenized manually with a glass homogenizer to prepare suspensions for the assay tubes (a blank tube, a standard tube, and an assay tube). The standard tube was filled with standard sample and assay working solution, and the assay tube was filled with the sample to be tested and assay working solution. They were mixed with a vortex mixer assay and placed in a water bath at 95°C for 40 min, centrifuged at 4000 rpm/min for 10 min, and placed in an enzyme marker at 530 nm to determine the OD value of each well.

### 2.9. HE Staining of Ovarian Tissue

Ovarian paraffin sections were dewaxed to water, and the sections were sequentially placed in xylene I for 20 min, xylene II for 20 min, anhydrous ethanol I for 5 min, anhydrous ethanol II for 5 min, and 75% alcohol for 5 min before washing with tap water. The slices were dried, dip stained in hematoxylin for 3 min, and washed with water. Hydrochloric acid alcohol fractionation was performed for 2 s. After washing with water and drying, eosin staining was conducted by immersion for 6 s and washing with water for 3 s, followed by 100% alcohol dehydration for 2 s. The slices were dried and sealed with neutral gum. An optical microscope was used to observe the results and acquire images.

### 2.10. Western Blot

Tissue homogenates were prepared by grinding 20 mg of ovarian tissue into ≤1 mm^3^ pieces, adding 180 *μ*L of tissue protein lysis solution, and lysing on ice for 1 h. The homogenate was centrifuged for 15 min (4°C, 12500 rpm), and the supernatant was removed. The protein concentration of the supernatant was determined using the BCA method and denatured by adding 5× loading buffer and boiling at 99°C for 10 min. Protein samples were separated with 10% SDS polyacrylamide gel and then transferred onto PVDF membranes. The membranes were blocked with blocking buffer for 20 min and incubated with the primary antibodies of TP53 (Abcam, ab26), IL-6 (Abcam, ab259341), and GAPDH (Abcam, ab8245) at 4°C overnight. The membranes were washed with TBST thrice for 10 min each time and then incubated with the corresponding secondary antibodies at room temperature for 1 h. Proteins were visualized with an ECL developer and a ChampChemi professional and automatic multicolor fluorescence and chemometric gel-imaging system. We then analyzed the gray area. The relative expression of proteins was determined as target protein/GAPDH.

### 2.11. UHPLC-QE-MS Analysis and Network-Pharmacology Analysis Predicting the Potential Gene Targets

#### 2.11.1. UHPLC-QE-MS Analysis

This analysis was performed on an Agilent UPLC 1290 UPHLC instrument. About 100 mg of sample was added to 500 *μ*L of the extracted solution containing 1 *μ*g/mL internal standard. The samples were homogenized at 45 Hz for 4 min and sonicated for 1 h in an ice-water bath. After placing for 1 h at -20°C, the samples were centrifuged at 12000 rpm for 15 min at 4°C. Finally, 300 *μ*L of the supernatant was extracted from the sample at 45 Hz for 4 min and sonicated for 1 h in an ice-water bath. Finally, 300 *μ*L of the supernatant was carefully filtered through a 0.22 microporous membrane and placed in a fresh 2 mL tube for LC-MS/MS analysis. The analysis was performed on an Agilent UHPLC 1290 system with a Waters UPLC BEH C18 column (1.7 *μ*m 2.1 × 100 mm). The flow rate was set at 0.4 mL/min, and the sample injection volume was set at 5 *μ*L. The mobile phase comprised 0.1% formic acid in water (*A*) and 0.1% formic acid in acetonitrile (B). The multistep linear elution gradient program was as follows: 0–3.5 min, 95%–85% A; 3.5–6 min, 85%–70% A; 6–6.5, 70%–70% A; 6.5–12 min, 70%–30% A 12 min, 70%–30% A; 12–12.5 min, 30%–30% A; 12.5–18 min, 30%–0% A; 18–25 min, 0%–0% A; 25–26 min, 0%–95% A; and 26–30 min, 95%–95% A. A QE Focus MS system coupled with Xcalibur software was used to obtain the MS and MS/MS data based on the IDA acquisition mode. During each acquisition cycle, the mass range was from 100 to 1500, and the top three of every cycle were screened. The corresponding MS/MS data were as follows: sheath gas flow rate, 45 Arb; aux gas flow rate, 15 Arb; capillary temperature, 400°C; full MS resolution, 70000; MS/MS resolution, 17500; collision energy, 15/30/45 in NCE mode; and spray voltage, 4.0 kV (positive) or −3.6 kV (negative).

### 2.12. Composition in HYJDF

HYJDF's constituent drug compound-related data were derived from the Traditional Chinese Medicine Systems Pharmacology Database and Analysis Platform (TCMSP), a unique herbal systems pharmacology platform that captures the relationship among drugs, targets, and diseases. The database includes wide-ranging information on chemical substances, targets, and drug-target networks [19]. From the database, we obtained the corresponding ingredients by entering the names of herbal medicines and then selecting the active ingredients for further analysis based on the criteria of oral bioavailability (OB) ≥ 30% and drug-likeness (DL) ≥ 0.18. Potential gene targets for the major compounds in HYJDF were searched from the TCMSP and PubMed databases.

### 2.13. EM-Associated Target Genes

EM targets were derived from two databases. The GeneCards (https://www.genecards.org/, GeneCards Version 5.6) database provides detailed information on all currently annotated, predictable genes [20]. The OMIM (http://www.omim.org/, Updated November 8, 2021) database contains all known genetic diseases and over 15 000 genes, with a focus on elucidating associations between disease phenotypes and causative genes [21]. Searches were conducted in both databases using the keywords “endometriosis,” “EM,” and “EMT” to obtain disease targets. An online Venn diagram tool (https://bioinfogp.cnb.csic.es/tools/venny/) was used to intersect and match EM disease-associated genes with the corresponding gene targets of HYJDF to obtain potential targets of HYJDF for EM treatment.

### 2.14. Construction of Component-Target-Disease Network

The component-target-disease network of HYJDF was constructed using the network-visualization software Cystoscope 3.8 to identify the core components of HYJDF. The highest active ingredients were selected by the software.

### 2.15. Enrichment Analysis and Ferroptosis Pathway Analysis

To explain the potential role of HYJDF in gene function and signaling pathways, we performed KEGG and GO enrichment analyses of screening targets using DAVID (https://david.ncifcrf.gov) [22]. The results of the enrichment analysis were visualized using the OE Biotech online analysis platform. Ferroptosis pathway analysis was screened for further analysis, and all ferroptosis-related genes were screened from the FerrDb database (http://www.datjar.com:40013/bt2104/) and intersected with the potential targets of the screened HYJDF-treated EM to obtain HYJDF-EM-ferroptosis triple common genes. Protein-protein interaction analysis was performed on the three shared genes using STRING (https://string-db.org/, version 11.0) to obtain the core target, which may be the core pathway of the HYJDF-EM-iron-death pathway governing EM.

### 2.16. Statistical Method

All statistical analyses were performed using one-way ANOVA followed by Dunnett's multiple comparison test, and all bar plots were generated with Graph Pad Prism 8 software. Data are expressed as the mean ± standard division. Statistical significance was set at *P* < 0.05.

## 3. Results

### 3.1. Construction of the Iron-Overload EM Model

#### 3.1.1. Analysis of Indicators Related to Ectopic Lesions in Iron-Overloaded Mouse

**Figure 3:**
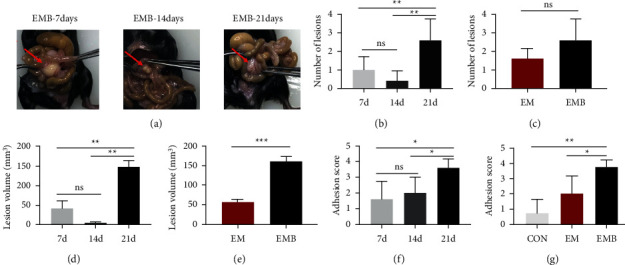
|Effectiveness of the iron-overload mouse model. (a) Morphology of lesions at different times in the EMB group. (b) Comparison of the number of ectopic lesions in the EMB group at different times. (c) Comparison of the number of ectopic lesions in each group. (d) Comparison of the volume of ectopic lesions in the EMB group at different times. (e) Comparison of the volume of ectopic lesions in each group. (f) Comparison of adhesion scores in the EMB group at different times. (g) Comparison of adhesion scores among groups. All data were expressed as mean ± standard division, n = 6 for each group. ^ns^*P* > 0.05,^*∗∗*^*P* < 0.01, and ^*∗*^*P* < 0.05.

Endometriotic foci formed in the EMB and EM groups 7 days after modeling with a 100% success rate. Most lesions grew on the mesentery and were occasionally observed near the subhepatic, sub splenic, abdominal wall, and uterine ovaries, forming adhesions with the surrounding tissue. The number and volume of mouse implants in the EMB group initially increased, then decreased, and increased again with prolonged modeling time compared with 7 and 14 days after modeling and 21 days after modeling. The number and volume of lesions increased significantly (*P* < 0.01) in the EMB group compared with the EM group at 7 and 14 days after modeling (Figures 3(b) and 3(c)). Compared with the EM group at 21 days after modeling, no significant change existed in the number of implants in the EMB group, but the volume of lesions increased significantly (*P* < 0.05) (Figures 3(d) and 3(e)). Mouse in the CON group showed mild adhesions, mouse in the EMB group showed moderate adhesions, and the degree of adhesions in the EMB group increased significantly (*P* < 0.01, *P* < 0.05) on day 21 after modeling compared with both groups (Figures 3(f) and 3(g)).

### 3.2. Total Iron Concentration in Peritoneal Lavage Fluid and Ovarian Tissue

**Figure 4:**
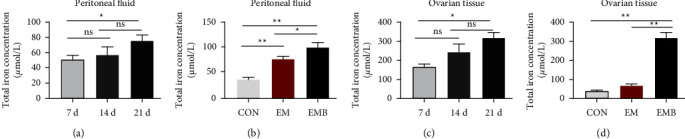
|The analysis of iron concentration in peritoneal fluid and ovarian tissue. (a) Comparison of total iron concentration in peritoneal fluid in the EMB group at different times. (b) Comparison of total iron concentration in the peritoneal fluid among groups. (c) Comparison of total iron concentration in ovarian tissue in the EMB group at different times. (d) Comparison of total iron concentration in ovarian tissue among groups. All data were expressed as mean ± SEM, n = 6 for each group. ^ns^*P* > 0.05,^*∗∗*^*P* < 0.01, and ^*∗*^*P* < 0.05.

The total iron concentration in the peritoneal lavage and ovarian tissues of the EMB group showed a gradual increase with prolonged modeling time, reaching the highest level at 21 days after modeling. The total iron concentration in the peritoneal lavage fluid and ovarian tissues of the EMB group increased significantly (*P* < 0.05) on day 21 compared with days 7 and 14 (Figures 4(a) and 4(b)). The total iron concentration in the peritoneal lavage fluid was significantly higher in the EM group at 21 days after modeling compared with the CON group at 21 days after modeling (*P* < 0.01), and the total iron concentrations in the peritoneal lavage fluid and ovarian tissues were also significantly higher in the EMB group at 21 days after modeling compared with the CON and EM groups (*P* < 0.05, *P* < 0.01) (Figures 4(c) and 4(d)).

### 3.3. Indicators Related to Ferroptosis in Ovarian Tissue

**Figure 5:**
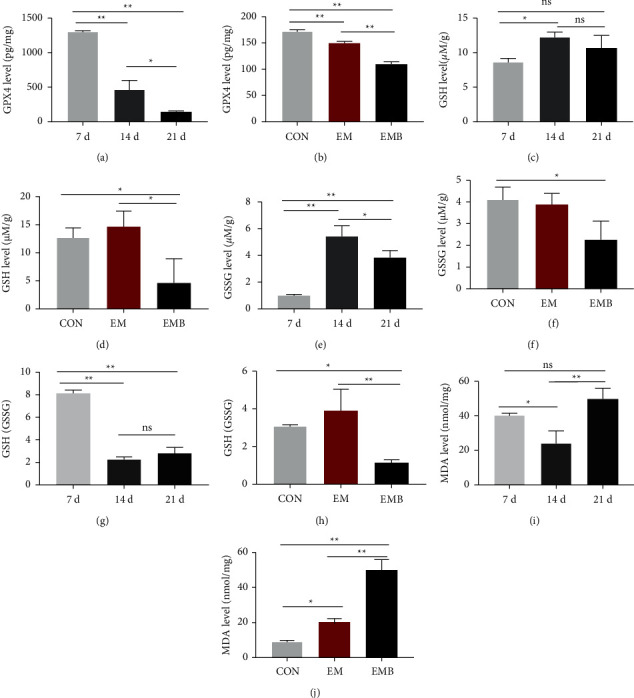
|Analysis of ferroptosis-related indicators. (a) Comparison of GPX4 levels in ovarian tissue in the EMB group at different times. (b) Comparison of GPX4 levels in ovarian tissue among groups. (c) Comparison of GSH levels in ovarian tissue in the EMB group at different times. (d) Comparison of GSH levels in ovarian tissue among groups. (e) Comparison of GSSG levels in ovarian tissue in the EMB group at different times. (f) Comparison of GSSG levels in ovarian tissue among groups. (g) Comparison of GSH/GSSG in ovarian tissue in the EMB group at different times. (h) Comparison of GSH/GSSG in ovarian tissue among groups. (i) Comparison of MDA levels in ovarian tissue in the EMB group at different times. (j) Comparison of MDA levels in ovarian tissue among groups. All data were expressed as mean ± SEM, n = 6 for each group. ^ns^*P* > 0.05,^*∗∗*^*P* < 0.01, and^*∗*^*P* < 0.05.

GPX4 expression in the ovarian tissues of EMB mice gradually decreased with prolonged modeling time. GPX4 expression significantly decreased at 14 and 21 days after modeling compared with 7 days after modeling (*P* < 0.05). GPX4 expression also significantly decreased at 21 days after modeling compared with 14 days after modeling (*P* < 0.05, *P* < 0.01) (Figure 5(a)). GPX4 expression in the ovarian tissues of a mouse in the EMB group was significantly lower than that in the CON and EM groups (*P* < 0.01) (Figure 5(b)). GSH concentration in the ovarian tissues of EMB mice initially increased and then decreased with prolonged modeling time. GSH concentration was significantly higher 14 days after modeling compared with 7 days after modeling (*P* < 0.05). GSH concentration in the ovaries of EMB mice showed the same trend as GSH concentration over the modeling time (*P* < 0.05, *P* < 0.01) (Figure 5(e)). Seven days after modeling, the GSH/GSSG ratio decreased (*P* < 0.01) and slightly increased 21 days after modeling compared with 14 days after modeling, but the difference was not statistically significant (Figure 5(g)). GSH concentration, GSSG concentration, and GSH/GSSG ratio were significantly lower in the EMB group compared with the CON and EM groups (*P* < 0.05, *P* < 0.01) (Figures 5(d), 5(f), and 5(g)). MDA concentration in the ovarian tissues of the mouse in the EMB group decreased significantly (*P* < 0.05) at 14 days after modeling compared with 7 days after modeling, and increased significantly (*P* < 0.01) at 21 days after modeling compared with 14 days after modeling (Figure 5(i)). MDA concentrations in ovarian tissues were significantly higher in the EMB group compared with the CON and EM groups (*P* < 0.05, *P* < 0.01) (Figure 5(j)).

### 3.4. *In Vivo* Validation of the Efficacy of HYJDF

#### 3.4.1. Analysis of Indicators Related to Ectopic Lesions

**Figure 6:**
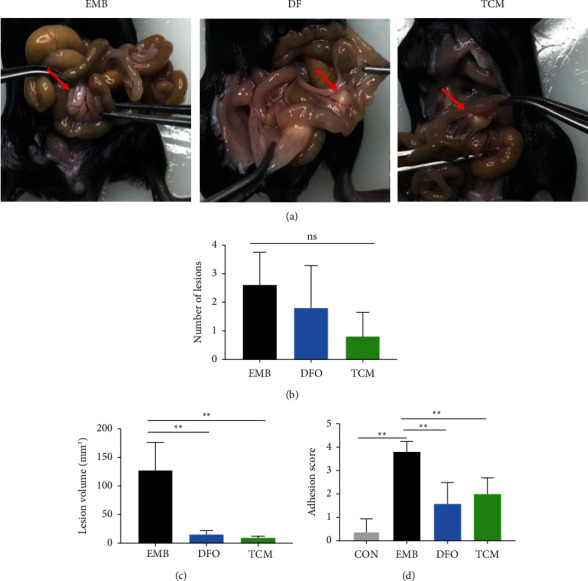
|Effectiveness of HYJDF treating EM. (a) Morphological indicators of ectopic lesions after treatment. (b) Comparison of the number of ectopic lesions in each group. (c) Comparison of the volume of ectopic lesions in each group. (d) Comparison of adhesion scores among groups. All data were expressed as mean ± SEM, n = 6 for each group. ^*∗∗*^*P* < 0.05.

At 21 days after modeling, the abdominal cavity of the mouse was opened and observed. Most endometriotic foci in the abdominal cavity of the mouse grew on the mesentery and were occasionally observed near the subhepatic, sub splenic, abdominal wall, and uterine ovaries, forming adhesions with the surrounding tissues. The number and volume of ectopic foci did not significantly differ between the model and treatment groups. The size of foci was significantly smaller in the DFO and TCM groups than in the EMB group (*P* < 0.01) (Figures 6(b) and 6(c)). Regarding the degree of adhesion, the adhesion score was significantly higher in the EMB group than in the CON group (*P* < 0.01), and adhesions were reduced in the DFO and TCM groups compared with the EMB group (*P* < 0.01) (Figure 6(d)).

### 3.5. Analysis of Iron Concentrations and Indicators Related to Iron Mortality

**Figure 7:**
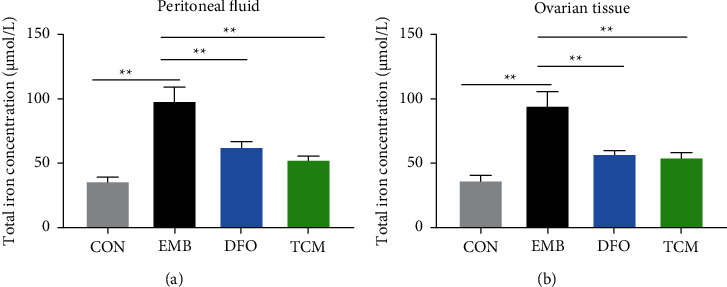
Analysis of ferroptosis-related indicators. (a) Comparison of GPX4 levels in ovarian tissue among groups. (b) Comparison of MDA levels in ovarian tissue among groups. (c) Comparison of GSH levels in ovarian tissue among groups. (d) Comparison of GSSG levels in ovarian tissue among groups. (e) Comparison of GSH/GSSG in ovarian tissue among groups. All data were expressed as mean ± SD, n = 6 for each group. ^*∗*^*P* < 0.05 and ^*∗∗*^*P* < 0.01

**Figure 8:**
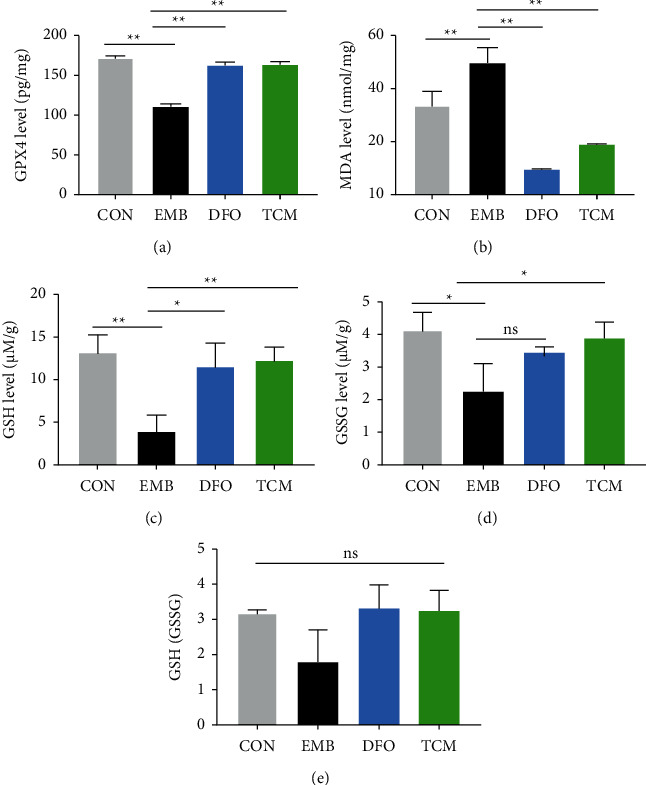
Analysis of ferroptosis-related indicators. (a) Comparison of total iron concentration in the peritoneal fluid among groups. (b) Comparison of total iron concentration in ovarian tissue among groups.

The total iron concentrations in the peritoneal lavage and ovarian tissues were significantly higher in the EMB group than in the CON group (*P* < 0.01). The total iron concentrations in the peritoneal lavage and ovarian tissues were significantly lower in the DFO and TCM groups than in the EMB group (*P* < 0.01). No significant differences were found between the DFO and TCM groups. After measuring GPX4 expression, GSH concentration, GSSG concentration, and MDA concentration in ovarian tissues, we found that GPX4 expression in ovarian tissues was significantly lower in the EMB group than in the CON group (*P* < 0.01). GPX4 expression was significantly higher in the DFO and TCM groups than in the EMB group (*P* < 0.01). GPX4 expression was significantly higher in the DFO and TCM groups than in the EMB group (*P* < 0.01). Compared with the CON group, the concentration of MDA in ovarian tissues of the mouse in the EMB group was significantly higher (*P* < 0.01), and compared with the EMB group, the concentration of MDA in ovarian tissues of the mouse in the DFO and TCM groups was significantly lower (*P* < 0.01) (Figure 7(b)). GSSG concentrations in the ovarian tissues of the mouse were significantly lower (*P* < 0.05, *P* < 0.01) in the EMB group than in the EMB group. GSH concentrations in the ovarian tissues of the mouse in the DFO and TCM groups were significantly higher (*P* < 0.05, *P* < 0.01), and the concentrations of GSSG in the ovarian tissues of the mouse in the TCM group were significantly higher (*P* < 0.05). Compared with the EMB group, the GSH/GSSG ratio in the ovarian tissues of the DFO and TCM groups increased, but the difference was not statistically significant (*P* > 0.05) (Figures 7(c)–7(e)). (see Figure 8)

### 3.6. Observation of Follicular Development

**Figure 9:**
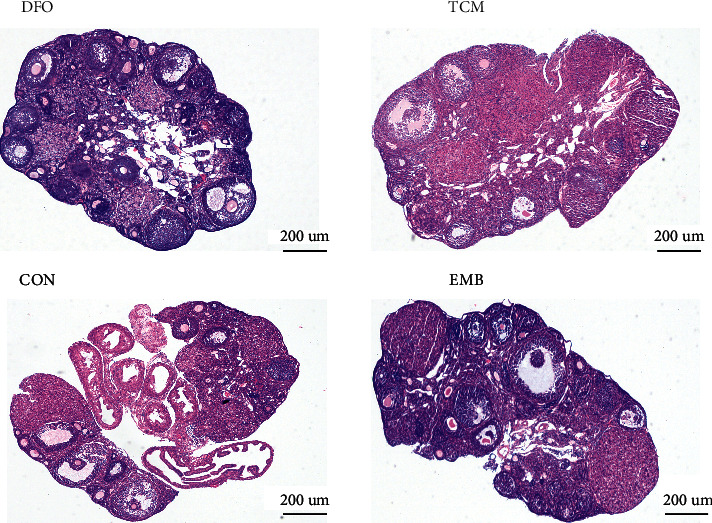
|Observation of follicle development in each group.

Sections with clear staining were selected to observe the various stages of ovarian follicular development and to count the number of primordial follicles, growing follicles, and luminal follicles (pre- and post-ovarian). The morphological criteria for each stage were as follows: primordial follicles, with a single layer of granulosa cells surrounding the oocyte; growing follicles, with the basement membrane separating the follicle cell stroma; early luminal follicles, with the appearance of a follicular cavity in the follicle; and late luminal follicles, with enlarged follicles, an enlarged follicular cavity containing follicular fluid, and granulosa cells surrounding the oocyte forming an oocyte mound (Figure 9) [23]. The percentage of follicles at each stage differed among groups, and the differences were statistically significant (*P*=0.043, <0.05) (Table 1). Compared with the CON group, the percentage of growing follicles was lower in the EMB group than in the CON group and higher in the DFO and TCM groups than in the EMB group (*P* < 0.05).

### 3.7. Screening of Bioactive Compounds by UHPLC-QE-MS Analysis

HYJDF components were analyzed using UHPLC-QE-MS analysis and tested for a combined score greater than 0.6 for inclusion in the analysis. Epimedium had the highest number of components, reaching 450 and 328 in positive and negative ion modes, respectively. *Herba* Epimedium followed, with 354 and 306 species in positive and negative ion modes, respectively. The number of positive and negative ions in the network-pharmacology screening was 309 and 259 for *Dahurian* vine, and 303 and 227 for *Phellodendron*, respectively (Supplementary Tables S1–S2). The core components of the network-pharmacology screening were all present in the assay, demonstrating the reliability of the network pharmacology analysis.

### 3.8. Herbal Compounds in HYJDF

A total of 240 HUJDF-related compounds were searched using the TCMSP database, among which 25 were from Dahaodeng, 33 were from Puhuang, 52 were from Sepia, and 130 were from Epimedium. A total of 45 chemical components were screened according to OB > 30% and DL > 0.18, 4 for hematoxylin, 6 for phellodendron, 12 for Herba Epimedium, and 23 for Epimedium. After eliminating the duplicates, 36 chemical components were finally obtained and included in further relevant target analyses (Supplementary Table S3).

### 3.9. Identification of HYJDF-Related Targets

**Figure 10:**
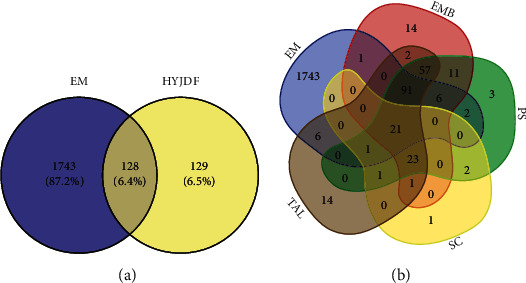
Overlaps between different gene sets. (a) Overlaps between EM diseases genes from HYJDF-related genes. (b) Overlaps between different gene sets from MI and 4 main herbs in HYJDF.

After removing duplicates, 1871 EM-related gene targets were obtained from the GeneCards and OMIM databases, including all genes currently identified or under study for EM. Genes shared by HYJDF and EM were considered as potential genes for the HYJDF treatment of EM, with a total of 128 genes (Supplementary Table S4). Figures 4(a) and 3(b) show the number of genes shared by HYJDF and EM and the number of shared genes contained in each herb (see Figure 10).

### 3.10. Construction of Component-Target-Disease Network and Core-Component Analysis

**Figure 11:**
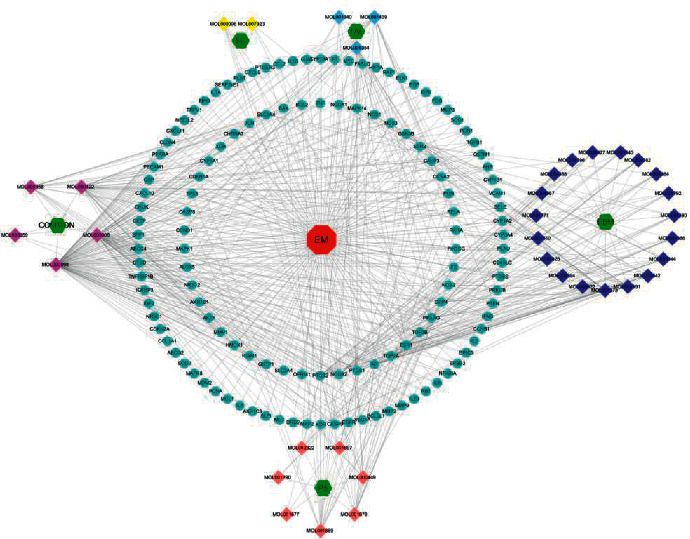
Potential compound-target-disease network of HYJDF in the treatment of EM. The network includes 1 disease, 5 herbal classifications, 36 compounds, and 128 predicted targets. The red square octagon represents the disease, the green square hexagon represents the herbal classification, the diamond represents the compounds (each color represents a different herbal medicine to which the compound belongs), and the light blue circle represents the potential target.

To explore the core components of therapeutic EM in HYJDF, a mesh construction of the above components and targets was performed using cystoscope to obtain a global view of the potential compound-target-disease network, including 170 nodes (1 disease, 5 herbal classifications, 36 compounds, and 128 predicted targets) and 600 edges (Figure 11). The network presented the correlation between the active ingredients of HYJDF and the target genes. All compound nodes in the network were analyzed for the degree, with higher degree values indicating a more significant role for the node in the network. Quercetin (degree = 98), shared by Icarium, Septoria, and Pueraria, as well as luteolin (degree = 46), shared by Icarium, Septoria, and Pueraria, had the highest degree values of all components, with Icarium, Septoria, and Pueraria in third place. The third highest value was for kaempferol (degree = 40), which was shared by Herba Epimedium and Herba Pulsatilla. Therefore, these three components were central to the therapeutic EM effects of HYJFD.

### 3.11. Enrichment Analysis and Ferroptosis Pathway Analysis

**Figure 12:**
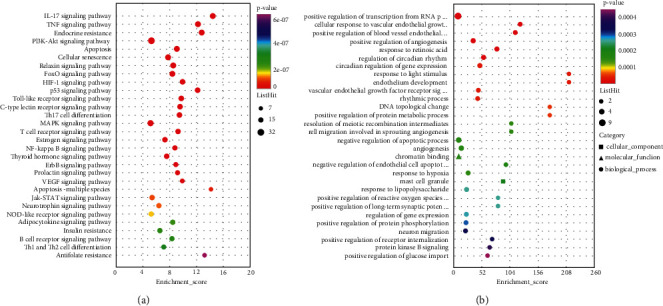
KEGG and GO enrichment analysis of the predicted targets of HYJDF for EM treatment. (a) Dots plot showing the first 30 KEGG pathways: the size of the dots corresponds to the enrichment score annotated in the entry, and the color of the dots corresponds to the corrected *P* value. (b) Dots plot showing the first 30 GO pathways: the size of the dots corresponds to the enrichment score annotated in the entry and the color of the dots corresponds to the corrected *P* value.

**Figure 13:**
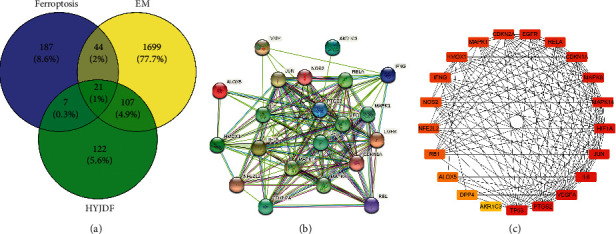
|Ferroptosis Venn diagram and ferroptosis core target diagram. (a) Overlaps between EM diseases genes, HYJDF-related genes, and ferroptosis-related genes. (b) String diagram of overlapping genes: more lines between genes means higher viability. (c) Hubba diagram of overlapping genes: the darker the color, the higher the degree value and the more significant the effect.

To explore the potential signaling pathways of HYJDF treatment of EM and the relevance of the iron-death pathway, the potential target genes obtained above were imported into the DAVID platform for KEGG versus GO analysis (Supplementary Tables S5 and S6). The top 30 GO and KEGG analyses are shown in Figures 12(a) and 12(b). Several EM-related pathways were involved in inflammation regulation, hormone regulation, angiogenesis, oocyte maturation, etc., such as TNF signaling pathway (path: hsa04668), estrogen signaling pathway (path: hsa04915), VEGF signaling pathway (path: hsa04370), and progesterone-mediated oocyte maturation (path: hsa04914). Further analysis revealed that ferroptosis (path: hsa04216) and response to iron-ion (GO:0010039) were present in the enrichment, confirming the association of HYJDF treatment of EM with the iron-death pathway. Conversely, multiple ferroptosis-related pathways such as the HIF-1 signaling pathway (path: hsa04066) were present in the enrichment (path: hsa04066). p53 signaling pathway (path: hsa04115) and Ras signaling pathway (path: hsa04014) were all identified as significant pathways in the enrichment analysis, further demonstrating the relevance of HYJDF to ferroptosis [24]. The three EM-HYJDF-ferroptosis genes were overlaid using the FerrDb database to obtain 21 targets (Figure 13(a)). A total of 21 targets were included in the string analysis, and the string relationship map was redrawn using Cystoscope to obtain 21 target-degree rankings (Figures 13(b) and 13(c)). The top four targets were TP53 (degree = 19), PTGS2 (degree = 19), VEGFA (degree = 18), and IL-6 (degree = 18).

### 3.12. Western Blot

**Figure 14:**
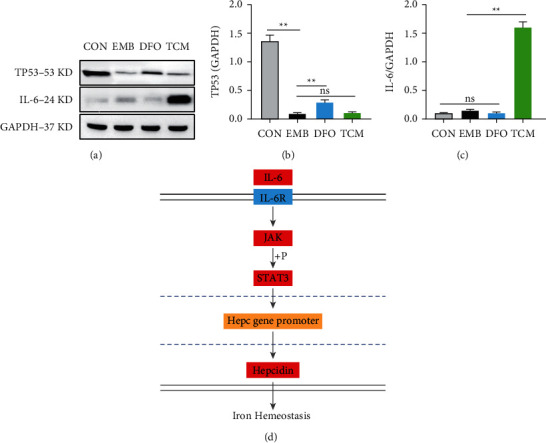
Western blot results. (a) Expressions of TP53 and IL-6 were determined by Western blot. (b) Quantitative analysis of TP53/GAPDH expression ratio. (c) Quantitative analysis of IL-6/GAPDH expression ratio. Data are shown as mean ± SD (n = 3). ^*∗*^*P* < 0.05, ^*∗*^*P* < 0.05 and ^*∗∗*^*P* < 0.01.

We subsequently investigated the effects of IL-6 and TP53 on the iron-death pathway. Results suggested that HYJDF did not regulate this pathway through the TP53 target, and no significant difference existed between the TCM and EMB groups. However, TP53 expression was significantly lower in the EMB group, whereas TP53 expression increased in the DFO group, suggesting that TP53 could inhibit ferroptosis in this model iron-death pathway (Figures 14(a) and 14(b)). WB results showed that HYJDF significantly increased IL-6 expression compared with other groups, suggesting that IL-6/hepcidin pathway signaling may be a potential mechanism for HYJDF to inhibit ferroptosis in EM and protect ovarian function, which requires further verification (Figures 14(a), 14(c), and 14(d)).

## 4. Discussion

EM is essentially an estrogen-dependent disease that may be triggered by the implantation of endothelial fragments caused by the retrograde flow of menstrual blood. The related pathological process is the periodic bleeding of ectopic lesions in response to estrogen and progesterone. The lysis of red blood cells in the blood, releasing hemoglobin and iron, leads to iron accumulation in the peritoneal tissue, as well as ectopic lesions and peritoneal fluid in EM patients, resulting in iron overload. This phenomenon affects macrophage clearance and thus promotes the growth of endometrial cells in the peritoneal cavity [25]. At the same time, excess iron can act as a catalyst to promote the production of a range of free-radical components through the Fenton reaction, mediating oxidative damage to normal tissue cells. In turn, the inflammatory environment brought about by iron overload and oxidative damage enhance the expression of divalent metal transporter-1 and others in the endometrium of EM patients, leading to the further development of EM [26]. The current study can be summarized in two ways. On one hand is the resistance of ectopic endometrial cells to nonapoptotic programmed cell death induced by iron overload, which allows endometrial cells that have reversed the flow of menstrual blood to survive, implant, and form ectopic lesions in the peritoneal cavity. On the other hand, is the contribution of dysregulated iron homeostasis to EM development, in which local iron overload and the consequent inflammation are associated with EM [27]. For example, iron overload may promote the migratory capacity of EM cells by upregulating the MMP expression of the ROS-NF*κ*B pathway [28]. Increased iron-ion concentration in the EM peritoneal environment decreases GPX4 expression and induces lipid peroxidation, which exerts toxic effects on the embryo and disrupts blastocyst formation.

Previous studies on iron overload in EM have focused on the effects of iron overload on EM disease, but few investigations have been conducted on the effects of iron overload on ovarian function. Moreover, most studies have used cellular models or EM mouse models to observe the effects of direct iron administration, which do not fully simulate the real EM disease state. Based on the above problems, we constructed a rodent iron-overload EM model based on the original rodent EM animal model by periodically injecting blood every 4 days according to the mean motility cycle time of the mouse to simulate the periodic bleeding state of EM lesions. The iron concentration in the peritoneal fluid and ovaries of this model significantly increased. Moreover, the number, size, and degree of adhesions of the lesions were significantly higher than those of mice in the EM modeling group, which was more similar to the actual pathophysiology of human EM. The iron-overload EM model was relatively simple and inexpensive to construct, and it can be replicated. Based on the successful construction of the model, we investigated the effect of iron overload on ovarian function in EM disease.

Chain radical reactions caused by iron overload can produce large amounts of toxic lipid peroxides that can inflict severe structural damage to cell membranes and other lipid membranes, resulting in cellular ferroptosis. ferroptosis is a new form of programmed death that occurs when excess free iron promotes the production of a series of ROS through the Fenton reaction. Excess ROS induces oxidative stress, leading to severe lipid peroxidation and eventual cell death. The biochemical features of ferroptosis are GSH depletion, GPX4 inactivation, and ultimately the iron-dependent accumulation of lipid peroxides [29, 30]. The current study found an imbalance of lipid peroxidation levels in the follicular fluid of EM patients, with weaker follicular resistance to lipid peroxidation and poorer follicular development [31]. Follicular-fluid transferrin levels in stage III/IV EM infertility significantly decreased, ferric ion levels increased, and iron overload existed. Meanwhile, follicular fluid in stage III/IV EM infertility caused a significant increase in ROS levels in mouse oocytes and a significant decrease in oocyte maturation rate, suggesting that TRF deficiency and iron overload in late-stage EM follicular fluid. This finding suggested that TRF insufficiency and iron overload in follicular fluid contributed to oocyte immaturity in advanced EMS FF, which may be a cause of EMS-related infertility [10]. We hypothesized that this phenomenon was due to the ovaries being affected by recurrent bleeding from pelvic and abdominal or ovarian ectopic lesions, resulting in elevated iron-ion levels in the ovarian and follicular fluid and inducing granulosa and oocyte ferroptosis.

This hypothesis was verified by our experiments, in which the ovaries of the EMB group showed a significant decrease in GPX4 and GSH levels, as well as a significant increase in MDA levels and a significant decrease in the number of growing follicles. Thus, iron overload may affect follicle development and growth through ferroptosis. After DFO and HYJDF treatment, the anti-lipid peroxidation capacity of the ovaries was enhanced, lipid-peroxidation response decreased, the percentage of growing follicles improved significantly, and fertility improved. These findings suggested that HYJDF and DFO could protect ovarian function and promote follicle development by inhibiting ferroptosis.

We further performed preliminary validation by identifying TP53 and IL-6 as the key targets of HYJDF for inhibiting ferroptosis through plasmapheresis and network pharmacology. Contrary to our initial assumptions, HYJDF did not strongly affect TP53 as a target, but TP53 gene affected the EM model mouse. TP53 expression was significantly inhibited in the EMB group compared with the CON group, and DFO improved this inhibition, suggesting that TP53 could improve ferroptosis in ovary-associated cells of EM iron-overloaded mice. Previous studies have shown that the regulation of ferroptosis by TP53 is complex, and studies on the reduction of ferroptosis sensitivity by TP53 have focused on p21 and DPP4. TP53 activates p21 in a transcription-dependent manner, and p21 acts on GSH, leading to increased intracellular GSH and increased GPX4 synthesis, resulting in decreased intracellular toxic lipid peroxides and reduced cellular sensitivity to ferroptosis. The p53–p21 pathway inhibits ferroptosis during metabolic stress [32]. TP53 limits ferroptosis by blocking DPP4 activity [33]. However, HYJDF has a stronger activating effect on IL-6 as IL-6 binds to IL-6 receptor *α* and activates Janus kinase to phosphorylate STAT3 protein, which enters the nucleus and binds directly to the corresponding site in the Hepc gene promoter to promote Hepc expression [34, 35]. Hepc is enhanced when the body is overloaded with iron, thereby increasing the synthesis and secretion of Hepc and accelerating the degradation of ferroprotein 1. It then closes the exit of iron to the blood, thereby maintaining iron homeostasis in the body [36]. We speculated that HYJDF may inhibit ferroptosis by reducing iron overload *in vivo* through the IL-6/hepcidin pathway, but we have conducted only preliminary studies, and more research on this pathway is needed. Notably, IL-6 in the follicular fluid can directly affect the oocyte–granulosa cell complex, which involves follicular oogenesis, oocyte maturation, oocyte quality, and ovulation [37]. This finding may explain, from another perspective, why HYJDF can promote follicular development.

The present study focused on constructing an EM iron-overload model to determine whether HYJDF could treat EM and improve ovarian function through the iron-death pathway. We conducted only a preliminary analysis of what genes HYJDF are regulated in the iron-death signaling pathway, and we will focus on this issue in subsequent studies. Herein, the model was constructed to better fit the physiological and pathological states, and this experiment used only animal models, not cellular validation, which will be supplemented by cellular experiments in future studies on specific mechanisms.

## 5. Conclusion

This study pioneered the method of periodic intraperitoneal blood injection to construct an iron-overload model for EM mice, which can better simulate the disease onset and progression state of EM. Based on the model, we found that in the iron-overload environment of EM, the ovarian function of the mouse was affected due to the occurrence of cellular ferroptosis. This process was reversed by HYJDF, which inhibited ferroptosis and improved ovarian function. Follow-up plasmatic and pharmacological analyses were conducted to analyze the effective monomers present in HYJDF and the key proteins that inhibited ferroptosis for preliminary validation. In the future, the specific mechanisms of iron-death regulation by the specific compound monomers identified in this experiment will be further investigated.

## 6. Disclosure

Jie Ding, Qianqian Zhao, and Zhihao Zhou share the first authorship.

## Figures and Tables

**Table 1:** Number of follicles at each stage in each group (*n* (%)).

Group	Ovaries number	Follicles total number	Primordial follicles	Growth follicles	Antral follicle	
					Early	Late
CON	6	54	13 (24.1)	26 (48.1)	9 (16.7)	6 (11.1)
EMB	6	41	17 (41.5)	6 (14.6)	11 (26.8)	7 (17.1)
DFO	6	39	14 (35.9)	17 (43.6)^*∗*^	2 (5.1)	6 (15.4)
TCM	6	50	13 (26.0)	19 (38.0)^*∗*^	11 (22.0)	7 (14.0)

*Note*.^*∗*^*P* < 0.05.

## Data Availability

The data that support the findings of this study are available from the corresponding author upon reasonable request.

## Supplementary Materials

Supplementary Materials
*Supplementary Table 1*. UHPLC-QE-MS analysis positive ions. *Supplementary Table 2.* UHPLC-QE-MS analysis negative ions. *Supplementary Table 3*. Compounds in HYJDF with OB > 30% and DL > 0.18. *Supplementary Table 4.* EM-related genes targeted by active compound of HYJDF. *Supplementary Table 5.* KEGG analysis. *Supplementary Table 6.* GO analysis.
