# Supplementary material for: Huayu Jiedu Fang Protects Ovarian Function in Mouse with Endometriosis Iron Overload by Inhibiting Ferroptosis
Source: Evid Based Complement Alternat Med. 2022 Aug 30;2022:1406820. doi: 10.1155/2022/1406820 (PMC9448539; doi:10.1155/2022/1406820)
Supplement: Supplementary Materials — Supplementary Table 1. UHPLC-QE-MS analysis positive ions. Supplementary Table 2. UHPLC-QE-MS analysis negative ions. Supplementary Table 3. Compounds in HYJDF with OB > 30% and DL > 0.18. Supplementary Table 4. EM-related genes targeted by active compound of HYJDF. Supplementary Table 5. KEGG analysis. Supplementary Table 6. GO analysis. [file 1406820.f1.pdf]

Supplement table 1. UHPLC-QE-MS Analysis Positive ions				
Compound name	yin yang huo	pu huang	da xue teng	pai jiang cao
:oxy-5-[(E)-3-(4-hydroxyphenyl)prop-2-enoyl]oxycy(	1	0	1	0
/droxy-6-methyl-2-(3,4,5-trihydroxyphenyl)-2,3-dihy	0	1	0	1
-7-propan-2-yl-3,4,10,10a-tetrahydro-2H-phenanthre:	0	0	0	0
5,7,7-trimethyl-4,5a,6,8,8a,9-hexahydro-1H-azuleno[	0	0	0	0
3-(2-Hydroxy-3,4-dimethoxyphenyl)-7-chromanol	1	0	1	1
5-Hydroxy-6,7-dimethoxyflavone	1	1	0	0
6,7,8-tetrahydroxy-2-(4-hydroxyphenyl)chromen-4-o:	1	1	0	0
:y-3-(3-hydroxy-4-methoxyphenyl)-6-methoxychrom(	0	0	0	0
7-Methoxycoumarin	0	1	0	0
oxy-1-methoxy-3-methylbut-3-enyl)-7-methoxychron	0	0	0	0
Aconitic Acid	0	0	0	0
Amphetamine	0	0	0	0
Apigenin	1	0	0	1
Bergaptol	0	0	0	0
Bergenin	1	0	0	1
Calycosin	0	0	0	0
cirsimaritin	1	0	0	0
D(-)-Salicin	0	1	0	1
Dihydromyricetin	1	0	0	1
Diosgenin	1	0	0	0
Fallacinol	1	1	0	0
Formononetin	0	0	0	0
Ginkgolic Acid C15:1	0	1	0	0
Gitogenin	1	0	0	0
Grosheimin	0	0	0	0
Hesperetin	1	0	1	0
Isolaserpitin	1	0	0	0
Isorhamnetin	1	1	0	0
Kaempferide	1	1	0	0
Kaempferol	1	1	1	1
kavain	0	0	0	0
L-Glutamate	0	0	0	1
methysticin	0	0	0	0
NEOSTIGMINE METHYL SULFATE	1	1	0	0
Norcimifugin	0	0	0	0
Osthole	0	0	0	0
Palmatine	1	0	0	0
Pectolinarigenin	0	0	1	0
Phloretin	0	0	0	0
picein	0	0	0	1
quercetin	1	0	1	0
Sinapoyl aldehyde	0	0	1	0
Swertiamarin	0	0	0	0
Threitol	1	0	0	1
Topotecan hydrochloride	1	0	0	0
Wikstromol	1	0	1	1
Nicotinic acid	0	0	0	1
Prunetin	0	1	0	0
4-Methylumbelliferone	1	1	0	0

3,4,5-trimethoxycinnamic acid	0	0	1	0
Rhamnetin	0	1	0	0
Scutellarein	1	1	1	1
Bergapten/Heraclin	1	0	1	0
Astragalin	1	1	0	0
phellopterin	0	0	1	0
-dihydroxy-1,4a-dimethyl-2-oxo-7-propan-2-yl-10,10	1	0	0	0
inermin	0	0	0	0
Bullatine A	0	0	0	0
Eupafolin	1	1	1	1
L-Proline	0	0	0	1
Naringenin	1	1	1	1
Maltol	0	0	0	0
Isoquercitrin	0	1	0	0
alpha-Cyperone	1	0	0	0
Arctigenin	0	0	0	1
Biochanin A	1	0	0	1
Morin	0	0	0	0
4S,5S,6R)-3,4,5-trihydroxy-6-(hydroxymethyl)oxan-2	1	0	0	1
Curdione	1	1	1	0
L-Ornithine	0	0	0	1
Atractylenolide II	1	0	1	1
Chrysin	1	0	0	0
Vitamin D3	1	1	0	1
ıydroxy-2-(4-hydroxyphenyl)-7-methoxy-2,3-dihydrc	1	0	0	0
Sauchinone	0	0	0	0
Demethoxycurcumin	1	0	1	1
Wilforlide A	0	0	1	0
Dehydrocostus lactone	1	0	0	0
Apiin	0	0	1	1
Musk ketone	0	0	0	1
L-Isoleucine	1	1	1	0
Flavanone base +2O, 1MeO	0	0	1	0
p-Coumaric acid	1	1	0	1
(+)-Nootkatone	1	1	1	1
Adenine	0	1	0	0
L-Valine	1	1	1	1
Cholic acid	1	1	0	1
alpha-Linolenic acid	1	1	1	0
Chlorogenic acid	0	0	0	0
Methionine	0	0	0	0
isoimperatorin	0	0	1	0
Myricetin	1	0	0	0
Isofraxidin	0	0	0	0
n-1(3H)-one, 4,4a,5,6,7,7a,8,9-octahydro-4,8-dihydrc	0	0	0	0
Tyrosine	1	1	1	1
Demethylwedelolactone	0	0	0	0
(2-hydroxypropan-2-yl)-7-methyl-2,3-dihydrofuro[3,2	0	0	0	0
Vitamin D2	1	1	0	1
Herbacetin	0	0	0	0
7,4'-DIMETHOXYISOFLAVONE	0	1	0	0

Ammothamnine	0	0	0	0
Oroxin B	1	0	0	1
etyloxy)methyl]-1-(beta-D-glucopyranosyloxy)-1,4a,	0	0	1	0
(+)-Magnoflorine	1	0	0	0
Isoalantolactone	0	0	0	0
Isoanhydroicaritin	1	0	0	1
4-Guanidinobutyric acid	1	0	1	1
GALACTITOL	1	1	1	0
Asiatic acid	0	0	1	0
Esculetin	1	1	0	0
Choline [M]+	1	1	1	1
5R)-12,15-dihydroxy-7-methyl-8-oxabicyclo[11.3.0]	1	1	1	1
Tectochrysin	0	0	0	1
4-Methylesculetin	1	0	0	1
Quercetin-3-O-glucoside	1	0	0	0
Linoleic acid	1	1	1	1
Isorhamnetin-3-O-nehesperidine	1	1	1	0
Emodin	0	0	0	0
beta-Elemonic acid	0	0	0	1
eudesmin	0	0	1	1
Pinocembrin	1	0	0	1
carbonyl-1,2,3,4a,5,6,7,8,9,10,12,12a,14,14a-tetrade	0	0	1	0
Benzamide	1	1	1	1
6-Hydroxyindole	0	0	0	1
alpha-Boswellic acid	0	0	1	0
Scoparone	0	0	0	0
cynaropicrin	0	0	1	0
H)-dione, 3a,7,7a,8,9,9a-hexahydro-5,8-dimethyl-3-n	0	0	0	0
Artemisinic acid	1	0	1	1
(5xi,9xi,10alpha)-Kaur-16-en-18-oic acid	0	0	1	0
Cinnamamide	0	0	0	0
Dehydroevodiamine	0	0	0	0
Kaempferol 3-glucorhamnoside	1	1	1	0
Pilocarpine	0	0	0	0
,6a,7a,8,9,9a-octahydrobisoxireno[4,5:8,9]cyclodeca	0	0	0	0
Cinnamaldehyde	0	0	1	0
Plumericin	0	0	0	0
Eriodictyol	1	0	1	1
Sophoricoside	0	0	0	1
Isosteviol	0	0	0	0
2-Hydroxyacetophenone	1	1	1	0
Lutl-6-C-Glc	1	1	0	1
Taxifolin	1	0	0	0
Afzelin	1	1	0	1
Glyceryl linolenate	0	1	1	1
Typhaneoside	1	1	0	1
S,5S,6R)-3,4,5-trihydroxy-6-(hydroxymethyl)oxan-2-	0	0	0	0
3-Pyridinemethanol	1	1	1	1
Daidzein	0	0	0	0
Caffeic acid	1	0	0	0
Hyperoside	1	1	1	1

Tectoridin	1	0	0	0
Hydroprotopine	1	0	0	1
Guanine	1	1	1	1
Delsoline	0	1	0	0
Isoflavone base + 3O	1	0	0	1
Clareolide	1	1	0	0
monoolein	0	1	0	0
Neostigmine bromide	0	1	0	0
Ferulic acid	0	1	0	0
7-trimethyl-3,4,4b,5,6,9,10,10a-octahydro-2H-phenan	0	1	0	0
Flemiphilippinin A	1	0	0	0
Acetyl-11-keto-beta-boswellic acid	0	0	0	0
Nicotinamide	1	0	0	1
3,4-Dimethoxybenzaldehyde	1	1	1	1
Coniferin	1	1	1	1
Grosvenorine	1	1	0	0
2''-O-beta-L-galactopyranosylorientin	1	1	0	1
Coumaric acid	1	1	1	0
Sterigmatocystin	1	0	0	0
Sissotrine	1	0	0	1
Rosarin	1	0	1	0
Isobergapten	1	0	0	0
judaicin (eudesmane naphthofuran)	1	0	0	0
Ursonic acid	0	1	0	0
aflatoxin B1	1	0	0	0
Arachidonic acid (not validated)	0	0	0	0
(+)-Cannabidiol	0	0	0	0
Cafestol	1	1	1	1
Pyocyanin	0	0	0	0
droxymethyl)-1,2,6a,6b,9,12a-hexamethyl-2,3,4,5,6,6	0	0	1	0
Ephedrine	0	0	0	0
kaempferol 7-neohesperidoside	1	1	1	1
7,8-Dihydroxyflavone	0	0	0	0
4,14a-tetradecahydropicen-3-yl]oxy]-5-[(2S,3R,4S,5R	1	1	0	1
Paeoniflorin	1	0	0	0
(3,4,5-trihydroxybenzoyl)oxyoxan-2-yl]methyl 3,4,5-	1	0	0	0
4-AMINOBUTANOIC ACID	0	1	0	1
ymethyl)-2,2,6a,6b,9,12a-hexamethyl-1,3,4,5,6,6a,7,8	1	0	0	0
Valine	0	0	0	1
ALLOMATRINE 1N-OXIDE	0	0	0	0
Praeruptorin A	0	0	1	0
2,3-dihydroxypropyl hexadecanoate	1	0	0	0
Ononin	1	0	0	0
Homoplantaginin	1	1	0	0
Oleanonic acid	1	0	0	0
Lindenenol	1	1	1	1
Glabrolide	0	0	0	0
,6,10-trimethyl-7,8,11,11a-tetrahydrocyclodeca[b]fur	0	0	0	0
Phenylacetaldehyde	1	1	1	0
Isatin	0	0	0	0
Flavone	1	0	0	0

licochalcone B	1	0	0	0
Fraxetin	0	0	0	0
Benzoic acid	0	0	1	0
)-2,3-dihydro-7h-furo[3,2-g]chromen-2-yl]propan-2-y	0	0	0	0
5-trimethoxyphenyl)-5a,6,8a,9-tetrahydro-5H-[2]ben:	1	0	0	0
3-Formylindole	1	1	1	1
Abietic acid	1	0	1	1
Leucoside	1	1	1	1
›SILON,NEPSILON,NEPSILON-TRIMETHYLLYS	0	1	0	1
Germacrone	0	1	1	0
Skimmin	0	0	1	0
Deoxyvasicinone	0	1	0	1
Anhydroicaritin	0	0	0	0
Kojic Acid	0	0	1	0
Corosolic acid	0	1	1	0
20(R)-Protopanaxdiol	0	0	0	0
N-Acetyl-DL-glutamic acid	1	0	0	1
-yl] (3aS,6Z,10E,11aR)-10-methyl-3-methylidene-2-(	0	0	0	1
Eicosapentaenoic acid	0	0	1	0
Deacetylasperulosidic acid	0	1	0	0
Shanzhiside methyl ester	0	1	0	0
Cinchonidine	0	0	0	0
18 beta-Glycyrrhetintic Acid	0	0	0	0
Linolenic acid ethyl ester	0	1	0	0
5b,9,9,12a-heptamethyl-2,3,4,5,6,6a,7,8,8a,10,11,12,1	1	0	1	0
yltetracyclo[11.2.1.0¹,¹⁰.0⁴,⁹]hexadecane-5,14-dicarb	1	1	1	1
Rutin	0	1	0	1
ydroxymethyl)-2,2,6a,6b,9,12a-hexamethyl-1,3,4,5,6,	1	1	1	1
Quercetin 3-O-beta-D-Glucuronide	1	0	0	0
(2E,4E)-N-(2-methylpropyl)deca-2,4-dienamide	1	1	0	0
(+)-Catechin hydrate	0	0	0	0
Lupenone	0	1	1	0
:y-2,2,6a,6b,9,9,12a-heptamethyl-1,3,4,5,6,6a,7,8,8a,	0	0	1	0
Oroxylin A-7-O-beta-D-glucuronide	0	0	0	0
Harmol	0	0	0	0
L-Tryptophan	1	1	1	1
Dihydroresveratrol	0	0	1	0
Isochlorogenic acid C	1	0	0	1
rhodioloside	1	0	1	0
Betulin	0	0	1	0
alpha-Hederin	0	1	0	0
Asperphenamate_120258	1	0	1	1
Docosahexaenoic acid	0	0	0	1
Pyridoxine	1	1	1	1
)ut-2-enyl)-7-[(2S,3R,4S,5S,6R)-3,4,5-trihydroxy-6-(	1	0	1	1
Coumaroyl tyramine	1	0	1	0
-1,2,6a,6b,9,9,12a-heptamethyl-2,3,4,5,6,6a,7,8,8a,1(	1	0	0	1
Diethyltoluamide	0	0	0	0
(5S)-5-hydroxy-1,7-diphenylheptan-3-one	0	0	0	1
Hyoscyamine	0	0	0	0
Phloroglucinol	0	0	0	0

droxy-6-(hydroxymethyl)oxan-2-yl]oxy-7-[(2S,3R,4I	1	1	0	1
Propranolol	0	1	0	0
ɔpropylidene-3,8-dimethyl-2,3,3a,4,5,8a-hexahydro-6	0	0	0	0
Racanisodamine	1	0	1	0
Curcumenol	1	1	0	0
Phthalic anhydride	1	1	1	1
·hydroxyphenyl)-6,8-bis(3-methylbut-2-enyl)-2,3-dih	1	0	0	0
trans-Zeatin-riboside	0	0	1	0
Diosgenin glucoside	1	0	0	0
Coniferyl aldehyde	1	0	1	0
Tauroursodeoxycholic acid	0	0	0	1
Cryptotanshinone	1	0	0	1
Cimifugin	1	0	0	0
Baohuoside I	1	0	0	0
Acetophenone	0	1	0	0
Flavonol base + 3O, O-Hex	0	0	0	0
L-Pyroglutamic acid	1	0	0	1
Arbutin	1	0	0	0
Securinine	0	0	0	1
Coumarin	0	1	1	1
Roburic acid	0	1	1	1
Phenethylacetate	0	0	0	0
,22R)-4,27-Dihydroxy-5,6:22,26-diepoxyergosta-2,2	0	1	0	0
Talatisamine	1	1	1	1
cuminyl alcohol	1	1	0	1
APIGENIN DIMETHYL ETHER	0	1	0	0
Methyl trans-cinnamic acid	1	1	0	1
ta-D-glucopyranosyloxy)-8-hydroxy-1,5-dimethyl-6-	0	1	1	0
3a-hydroxy-2,5,8,8,12-pentamethyl-2,3,3a,4,5,8,9,10	0	0	0	1
Higenamine	1	0	1	0
Vanillic acid (not validated)	1	0	1	0
Pyroglutamic acid (not validated, isomer of 88)	0	0	0	1
Ginkgolic acid C17-1	1	0	0	1
ADONITOL	0	0	0	0
Kaempferol 3-O-sophoroside	0	0	0	1
Thymol	1	0	0	0
·hexen-1-one, 4-hydroxy-4-(3-hydroxybutyl)-3,5,5-tri	0	0	0	1
Pimara-9(11),15-dien-18-oic acid	0	0	0	0
1-Monostearin	1	1	1	1
Camelliaside B	0	1	0	0
Isovanillic acid	0	1	1	1
OBLIQUIN	0	0	0	0
Byakangelicol	0	0	1	0
Phenylalanine	1	1	1	1
Tuberostemonine	0	1	0	1
Carveol	1	0	1	1
,8-dimethyl-5-propan-2-ylidene-1,2,3a,4,7,8a-hexahy	0	0	1	0
dihydroxyphenyl)prop-2-enoyl]oxy}-1,3,5-trihydrox	0	0	0	0
Isoleucine	0	1	0	0
Karakoline	0	0	0	0
Deoxynivalenol	0	0	0	0

Gamabufotalin	1	0	0	0
LYSINE	0	1	0	1
asperuloside	0	0	0	0
Camphor	0	0	0	0
Liquiritigenin	1	0	1	0
Paracetamol	1	0	1	0
osmanthuside H	1	1	1	0
,7-dimethoxy-2-methyl-3,4-dihydroisoquinolin-1-on	0	0	1	1
Methyl linoleate	0	0	0	0
7,8-Dihydroxycoumarin	0	0	0	0
Steviol	0	0	0	0
nethyl-7-(4-methyl-3-penten-1-yl)bicyclo[2.2.1]hept	1	1	0	0
xyoxan-2-yl]oxyoxan-2-yl]oxy-4,5-dihydroxyoxan-2	0	0	0	0
Brazilein	0	0	0	0
Quillaic acid	0	0	1	0
-[(E)-3-(4-hydroxy-3-methoxyphenyl)prop-2-enoyl]	1	1	1	0
Cianidanol	1	0	0	0
6-en-2-yl)-7,7,12,16-tetramethylpentacyclo[9.7.0.01,	0	0	0	1
Boldine	1	0	0	0
l]oxy-1,5-dihydroxy-4-[(E)-3-(4-hydroxy-3,5-dimeth	0	0	1	0
Bufalin	0	1	0	1
Hippuric acid	1	0	1	1
Geranic acid	0	0	0	1
Isoschaftoside	1	1	0	1
Progesterone	1	0	0	0
rimethyl-2-methylidene-3,4,4a,6,7,8-hexahydro-1H-n	1	0	0	0
Veratramine	0	0	0	0
Rosamultin	0	0	0	0
Tramadol	0	1	0	0
ıethoxy-3-methyl-5-[(1E)-1-propen-1-yl]-2,3-dihydro	1	1	1	1
ran-1(3H)-one, 4,4a,5,6,7,9-hexahydro-3,4-dihydrox	1	1	1	1
TAXIFOLIN-(+/-)	1	0	1	0
Cinnamic acid	0	1	0	0
ı_C12H15NO4_3,7-Benzofurandiol, 2,3-dihydro-2,2	0	1	0	1
DL-Pipecolinic acid	0	0	1	1
5-Hydroxymethylfurfural	0	0	1	0
Scopoletin	0	1	0	0
-trihydroxy-6-(hydroxymethyl)oxan-2-yl]oxy-1,4a,5,	0	0	0	0
Schizandrin A	0	0	1	0
Solasodine	1	1	0	1
Ethyl 4-methoxycinnamate	0	1	1	0
ıyl-1a,2,3,4a,5,6,7a,7b-octahydrocyclopropa[h]azule	0	1	0	0
Guanosine	1	1	1	1
Daidzin	0	0	0	0
yl-4,4a,5,6-tetrahydro-1H,3H-pyrano[3,4-c]pyran-6-y	1	0	1	0
Phenelzine	0	1	0	0
Vasicine	0	1	0	1
Isomeranzin	0	0	0	0
Kaempferol-3-O-alpha-L-arabinoside	1	1	0	0
oxo-7-propan-2-yl-2,3,4,4b,5,6,10,10a-octahydrophe	0	0	0	0
imethyltricyclo[2.2.1.0~2,6~]hept-3-yl)-2-methyl-2-	0	0	0	0

Resibufogenin	0	0	0	0
N-Methyl-2-pyrrolidone	1	1	1	1
4-Phenyl-3-buten-2-one	0	0	0	0
Flavone base + 3O, O-HexA	1	0	0	0
ethylidene-6,18-dioxatricyclo[14.2.1.0⁵,⁹]nonadeca-3	0	0	0	0
Kaempferol-3-O-glucoside-6"-p-coumaroyl	1	0	0	0
Procyanidin B1	1	0	1	0
1,3-Dicaffeoylquinic acid	0	1	0	0
Perillene	0	1	0	0
Mangiferin	0	0	0	0
Sulfuretin	1	0	1	0
Phenylpropanolamine	0	1	0	0
Isochlorogenic acid B	1	0	0	0
methoxyphenyl)methyl]-3-(hydroxymethyl)oxolan-2	0	0	1	1
Loliolide	1	1	0	0
Cytosine	1	0	0	0
Androsin	0	0	1	0
Adenosine monophosphate	0	1	0	1
Oxysophocarpine	0	0	0	0
o-Anisic acid	0	0	0	0
Piperlongumine	0	0	1	0
Spermine	0	0	0	0
imethylhept-3-en-2-yl]-6,10-dimethyl-16,17-dioxape	0	1	0	0
(5xi,9xi)-12-Hydroxyabieta-7,13-dien-18-oic acid	1	1	1	1
Antheraxanthin	1	0	0	0
Methyl vanillate	0	0	0	0
[(2R,3R)-2,3,6-trihydroxy-6-methylheptan-2-yl]-2,3,	1	0	0	1
Isophorone	1	0	0	0
4-Androstene-3,17-dione	0	0	0	0
Tyramine	0	1	1	1
[3,4,5-trihydroxy-6-[(3,4,5-trihydroxy-6-methyloxan	1	0	0	0
loganic acid	1	1	1	1
Demissidine	0	1	0	1
Psoralen	0	0	0	0
Meperidine	1	0	0	0
syringaresinol	1	1	1	1
p-Hydroxybenzaldehyde	0	0	0	0
Echinocystic acid	0	0	0	0
oyl]oxyethylidene]-6-[(2S,3R,4S,5S,6R)-3,4,5-trihyd	0	1	1	0
Foliosidine	0	0	0	1
ESTRADIOL	1	1	0	1
Esculentoside A	0	0	1	0
-dihydroxy-4-(hydroxymethyl)oxolan-2-yl]oxymethy	0	0	0	0
Dubinidine	1	0	1	1
Lupinine	0	1	0	1
Dihydroartemisinic acid	1	1	1	1
droxy-7,8-dimethoxy-2-phenyl-2,3-dihydrochromen-	1	0	1	0
Gentiopicroside	0	0	0	0
(-)-Huperzine A	0	0	0	0
2-enoyloxy)-2-oxo-9,10-dihydropyrano[2,3-f]chrome	1	0	1	0
Ruscogenin	0	0	0	0

6-methoxy-7-(3-methylbut-2-enoxy)chromen-2-one	0	0	0	1
Methyltestosterone	1	0	0	0
suberosin	1	0	1	1
Beta-Caryophyllene Alcohol	0	1	1	0
Protocatechualdehyde	1	1	1	1
Silibinin	0	0	0	0
-2-yl-2,3,6,7,8,8a-hexahydropyrrolo[1,2-a]pyrazine-1	0	1	0	1
Dihomo-γ-linolenic acid (DGLA; cis-20:3n-6)	0	0	0	0
Hydroxyvalerenic Acid	0	1	0	1
Ethylparaben	0	0	0	0
Hecogenin	0	0	0	1
Dibutylphthalate	1	0	1	1
Acetylcholine	0	1	0	0
nethyl-1a,3,6,10a-tetrahydrooxireno[4,5]cyclodeca[1	1	1	1	1
Isoastragaloside I	1	1	1	1
Epicatechin	1	1	1	0
hydroxy-9-(hydroxymethyl)-2,6a,6b,9,12a-pentametl	0	0	1	0
Petunidin-3-O-beta-glucopyranoside	1	1	1	1
d, 7-ethenyl-1,2,3,4,4a,4b,5,6,7,9,10,10a-dodecahydr	0	0	0	1
y-5-[(Z)-2-(4-hydroxyphenyl)ethenyl]phenoxy]-6-(hy	0	1	1	1
Desoxypeganine	0	0	0	0
-dihydroxy-16-methyl-2,4,14,19-tetraoxahexacyclo[8	1	0	0	0
Irinotecan hydrochloride	0	0	0	1
Sorbitol	0	1	1	0
-isopropylidene-3,8-dimethyl-2,3,3a,4,5,8a-hexahydro	0	1	0	0
1,7-bis(4-hydroxyphenyl)heptan-3-one	0	0	0	0
Polygalic acid	0	0	0	0
Auraptene	1	0	0	0
Lawsone	0	0	0	0
R,4S,5S,6R)-3,4,5-trihydroxy-6-(hydroxymethyl)oxa	1	0	1	0
Procyanidin B2	1	0	0	0
Quercetin-3-D-xyloside	0	1	0	0
lpropyl)-2,3,6,7,8,8a-hexahydropyrrolo[1,2-a]pyrazin	0	0	0	0
Acacetin-7-O-neohesperidoside	1	0	1	1
Lysionotin	0	0	0	1
Biochanin-7-O-glucoside	0	0	0	1
Taurocholic acid sodium salt hydrate	0	0	0	0
AGMATINE SULFATE	1	1	1	1
ASPARTATE	1	1	1	1
opan-2-yl)-1,4a-dimethyl-2,3,4,5,6,7,8,8a-octahydroi	0	0	0	1
Napelline	0	0	0	0
Norisoboldine	0	0	0	0
2'-Deoxyadenosine	1	1	0	1
Handelin	0	0	1	0
Ursolic acid	0	0	1	0
Scutellarin	1	0	0	0
Erucamide	1	0	1	1
Phenylethyl primeveroside	1	1	1	1
Valerophenone	1	0	0	1
Artemetin	0	0	0	1
Kaempferitrin	1	1	0	0

Ligustilide	1	0	0	0
delta-Dodecalactone	0	1	1	0
Stachydrine	1	1	0	1
Huperzine B	1	1	1	1
Cantharidin	1	1	1	1
Ecgonine	0	1	0	1
aucubin	0	0	0	0
beta-D-Glucopyranoside, 4-(hydroxymethyl)phenyl	1	1	0	0
Sarsasapogenin	1	0	0	1
2-hexyl-5-propyl-resorcinol	0	1	1	1
-8-methylene-2,3,6,7,7a,8,10a,10b-octahydrooxireno[	1	0	0	0
Morusin	1	0	0	0
5-O-Demethylnobiletin	0	0	0	0
N-(1-hydroxy-3-phenylpropan-2-yl)benzamide	1	0	0	1
Curcumol	1	0	0	0
Atractylenolide I	0	0	0	1
(+)-Pinoresinol	0	0	0	0
-6,14-dioxo-7,13-dioxatricyclo[10.2.1.0⁴,⁸]pentadeca-	1	0	0	1
Alisol B 23-acetate	0	0	1	0
Anthranilic acid	0	0	0	0
-furan]-2,5'(3H)-dione, octahydro-8-hydroxy-6,8-din	0	0	1	0
Dihydro-8-deoxy-lactucin	0	0	0	1
1,3,3a,4,6,6a-hexahydrofuro[3,4-c]furan-6-yl]-2-meth	0	0	0	0
Pinoresinol 4-O-glucoside	1	0	0	0
Vasicinone	0	1	0	1
Jervine	0	0	0	0
Neoruscogenin	1	0	0	0
Androstane-3,17-diol	0	0	0	0
Trigonelline HCl	1	1	0	1
Epibrassinolide	0	1	0	0
salviaflaside	0	0	0	0
Tomatidine	1	1	1	1
Bruceine D	0	0	1	0
ıydroxy-6-(hydroxymethyl)oxan-2-yl]oxy-1,5,6,7a-te	1	1	1	1
4,5-trimethoxyphenyl)-1,3,3a,4,6,6a-hexahydrofuro[3	1	1	1	1
Khellin	1	0	0	0
Arginine	0	0	1	0
GLUCOSAMINE	1	1	1	1
Flavokawain b	0	0	0	0
Tetramethylpyrazine	0	1	0	0
)-4,8-Dihydroxy-3,6,9-tris(methylene)decahydroazule	0	0	0	0
gamma-Linolenic acid	1	0	1	1
3,4-dihydroxyphenyl)-3,7-dihydroxy-2,3-dihydrochrc	1	1	1	1
Vitexin rhamnoside	0	1	0	0
Azelaic acid	0	0	0	0
-trihydroxy-6-[[(2R,3R,4R,5R,6S)-3,4,5-trihydroxy-6	1	1	0	1
16-Oxo-19-beyeranoic acid	1	0	1	0
Echinenone	1	1	0	1
Adenosine	1	1	1	1
(+/-)-Jasmonic acid	0	0	0	0
3-Hydroxybenzaldehyde	0	0	0	1

Nardosinone	0	0	1	0
Cearoin	0	0	0	0
D-Pantothenic acid	1	1	1	1
Tryptamine	1	1	0	1
P-Anisic acid	0	0	1	0
25-Tetrahydroxy-9,10,14-trimethyl-4,9-cyclo-9,10-se	0	0	0	1
Wedelolactone	1	1	0	0
Periplogenin	1	0	1	1
Apigenin-7-O-beta-D-glucoside	1	0	1	1
Trehalose	0	1	0	0
Zizyberanalic acid	0	1	0	0
HUMULENE (alpha)	0	0	0	0
Noreugenin	1	0	1	0
(-)-12-hydroxyjasmonic acid	1	1	0	1
roxy-5-[(E)-3-(4-hydroxyphenyl)prop-2-enoyl]oxycy	1	0	0	0
Linalool	1	0	0	0
geniposide	0	0	1	0
Methyl 3,4,5-trimethoxycinnamate	1	0	1	1
Calceolarioside B	1	0	1	1
,8-trimethyl-4a,5,6,7,7a,8,9,9a-octahydroazuleno[6,5-	0	0	0	0
O-(3-Hydroxy-7-drimen-11-yl)umbelliferone	1	0	0	0
Piperanine	0	0	0	0
Hinokitiol	0	0	1	0
Isovitexin 2''-O-arabinoside	1	1	0	1
Ginkgolide B	1	0	0	1
3,4-Dihydroxy-L-phenylalanine	1	1	0	1
Domoic acid	0	0	0	0
Ginkgolic acid (C13:0)	0	0	0	0
4,5-trihydroxy-6-(hydroxymethyl)oxan-2-yl]oxy-2,5a,	0	0	0	0
Dihydrokaempferol	0	0	1	1
Bassanolide	1	0	0	1
xy-2-(4-hydroxyphenyl)-8-(3-methylbut-2-enyl)-2,3-	1	0	0	0
methyl)-5,9-dimethyltetracyclo[11.2.1.0¹,¹⁰.0⁴,⁹]hexa	1	0	0	0
Toddalolactone	0	0	0	0
3-methoxyphenyl)-4-(hydroxymethyl)oxolan-3-yl]me	1	0	1	1
Oxypeucedanin hydrate	0	0	0	0
Oleamide	1	0	1	1
Betulinic Acid	1	0	1	0
oxyphenyl)-3,5-dihydroxy-3,4-dihydro-2H-chromen-	0	0	1	0
Ursodeoxycholic acid	0	0	0	0
linderalactone	0	1	0	0
LOVASTATIN	0	0	1	1
Ethyl ferulate	0	0	0	0
15-trihydroxy-10,14,17,17-tetramethyl-11-oxo-6-oxa	1	0	0	1
Alisol C 23-acetate	0	1	0	0
-29-methoxy-29-oxo-3-(pentopyranosyloxy)olean-12	0	1	0	0
oxan-2-yl]oxy-14-hydroxy-10,13-dimethyl-1,2,3,4,5,	0	0	0	0
4-[[(2E)-3-(4-hydroxy-3-methoxyphenyl)-1-oxo-2-pr	0	0	0	0
(-)-Sinoacutine	0	0	1	0
Kanzonol C	0	0	0	0
thyl)-6-[4-(4-hydroxy-2,6,6-trimethylcyclohexen-1-y	0	0	1	0

L-Phenylalanine	0	0	1	0
Poricoic acid A	1	0	0	1
Bixin	1	0	0	0
Aminopyrine	1	0	0	1
Glycocholic acid	0	0	0	0
Moluccanin	0	0	0	0
Maslinic acid	1	0	0	1
Methyl jasmonate	0	0	1	0
Testosterone	0	1	0	1
Acacetin	0	0	0	1
Phellodendrine	1	0	1	1
Bullatine G	0	0	0	0
Austricine	0	0	0	1
COLFORSIN	1	0	0	1
dehydroabietic acid	1	0	1	0
-4,7-dimethylspiro[5,6,7,8a-tetrahydro-1H-naphthale	0	0	0	0
Nuciferine	1	0	0	0
ıyl-7-propan-2-yl-2,3,4,9,10,10a-hexahydrophenanth	1	1	0	0
LPC 16:0	0	0	0	0
gamma-Undecalactone	0	1	0	1
›lan-2-yl]oxy-3,4,5-trihydroxyoxan-2-yl]methyl (E)-3	0	1	0	0
Dihydrocapsaicin	0	0	0	1
12-oxo-phytodienoic acid	1	0	0	1
Sesamoside	1	0	0	1
1,7-bis(3,4-dihydroxyphenyl)heptan-3-one	0	0	0	0
hydroxy-3-[(2S,3R,4S,5S,6R)-3,4,5-trihydroxy-6-(hy	1	0	0	0
ydroxy-5-(hydroxymethyl)oxolan-2-yl]oxymethyl]-6-	1	1	0	0
8-O-Acetylharpagide	0	0	0	0
Isoeugenol acetate	0	0	0	1
Rosmarinic acid	0	1	0	1
4-Hydroxybenzoic acid	1	1	1	0
4-Hydroxyisoleucine	0	0	1	0
4-methoxyphenyl)propan-2-yl]oxy-6-(hydroxymethyl	1	0	0	0
p-Coumaraldehyde	0	0	0	0
2-Hydroxy-4-methoxybenzaldehyde	1	0	0	1
ıyl)methyl]-4-methoxy-2-(methoxymethyl)butyl]-4-m	1	1	1	1
COSTUNOLIDE	0	1	0	0
nide, N-[2-(acetyloxy)-1-(phenylmethyl)ethyl]-alpha	1	1	1	1
Sakuranetin	1	1	0	1
Medicagenic acid	0	0	1	0
Phenmetrazine	1	1	0	1
Canthaxanthin (Euglenanone)	1	1	0	0
Carpachromene	1	0	0	0
1-methyl-2-undecylquinolin-4-one	0	0	0	1
Ailanthone	1	0	1	1
Bryodulcosigenin	0	0	0	0
Peiminine	1	1	1	1
Quinoline	0	0	0	0
Icarisid I	1	0	0	0
(-)-Gallocatechin	1	0	0	0
-hydroxy-3-methylbut-3-enyl)-7-methoxychromen-2-	0	0	0	0

gamma-Decalactone	0	0	0	0
SECOISOLARICIRESINOL	1	0	1	0
1-Caffeoylquinic acid	1	0	1	0
Sinapoylcholine	0	0	0	1
Isoferulic acid	0	0	0	1
1-Methoxy-3-carbaldehyde	0	0	0	1
Steviol-19-O-glucoside	0	0	0	0
Sinapoylhexoside	1	1	0	0
Epmedin C	1	0	0	0
Allethrin	1	0	0	0
Inosine	0	0	0	0
ESTRIOL	1	0	1	1
Androsta-1,4-Dien-3,17-Dione	0	1	0	1
Sarracenin	0	0	1	0
Pyrocatechol	0	0	0	0
uercetin-3-O-beta-D-glucose-7-O-beta-D-gentiobiosi	0	0	0	1
sclareol	0	0	0	0
bilobol	0	0	0	0
7-methoxy-9,10-dihydrophenanthrene-2,5-diol	0	0	0	0
delta-Decalactone	1	0	1	0
-Furyl)-3a,7-dimethyl-3a,4,5,6-tetrahydro-2-benzofu	0	0	0	0
Ingenol-5,20-acetonide-3-O-angelate	0	0	0	1
POTASSIUM SORBATE	1	0	0	0
Acetylvanillin	0	0	0	0
)-5,8a-dimethyl-2-methylidene-3,4,4a,6,7,8-hexahyd	0	0	1	0
14-Deoxyandrographolide	0	0	0	0
Dictamnine	0	0	1	1
droxy-4-(hydroxymethyl)oxolan-2-yl]oxy-2-(hydroxy	1	0	1	0
Rutaevin	1	0	0	0
Eurycomalactone	1	0	0	1
Icariin	1	0	0	0
Syringic acid	1	0	1	0
2S,3R,4S,5S,6R)-3,4,5-trihydroxy-6-(hydroxymethyl)	0	0	1	0
Indigo	0	0	0	0
Cytisine	0	0	0	0
hydroxy-6-(hydroxymethyl)oxan-2-yl]oxy-1,5,6,7a-te	0	0	0	0
Neoline/Bullatine B	0	0	0	1
Flavokawain C	0	0	1	0
Myricetin-3-Galactoside	1	0	0	0
y-6-[5-hydroxy-3-(4-hydroxyphenyl)-4-oxochromen-	1	0	0	1
beta-D-Glucopyranoside, (3Z)-3-hexen-1-yl	0	0	0	0
5-(hydroxymethyl)-5,8a-dimethyl-2-methylenedecah	0	0	1	0
R)-3,4,5-trihydroxy-6-(hydroxymethyl)oxan-2-yl]oxy	1	1	0	1
Perillartine	0	0	0	0
Apigenin-6-C-glucoside-7-O-glucoside	0	0	0	1
Eugenol	1	1	0	1
-2-one, 3,3a,4,7,8,8a-hexahydro-6-(3-hydroxybutyl)-	0	0	1	0
Histamine	0	0	1	1
ropan-2-yl)-8a-methyl-4-methylidene-1,2,3,4a,5,6,7,8	0	0	0	1
Atenolol	0	0	0	0
retronecine	0	0	0	0

Fusaproliferin_130137	1	1	1	0
Penicillic Acid	1	1	0	1
-Dihydroxyphenyl)-2-propenoyl]oxy}-1,3,5-trihydro:	0	0	1	0
Gardenin B	1	0	0	1
Daurisoline	1	0	0	1
3-Hydroxyflavone	0	0	0	0
alpha-Santonin	1	1	1	1
Scopolamine butylbromide	1	0	0	1
Capsaicin	0	1	0	0
xo-9,10-dihydropyrano[2,3-h]chromen-9-yl) (Z)-2-m	1	0	0	0
Isolicoflavonol	1	1	0	0
Totarol	0	1	0	1
monolinolein	1	1	0	1
5-deoxy-α-L-mannopyranosyl-(1->4)-[β-D-glucopyra	1	0	0	1
Salicylic acid	1	1	1	1
Quercetin-3-(6"-malonyl)-Glucoside	1	1	1	1
Cephalotaxine	1	0	0	0
Complanatuside	1	0	0	1
jaeschkeanadiol	1	0	1	1
CHOLESTEROL	1	0	1	0
3R,4S,5S,6R)-3,4,5-trihydroxy-6-(hydroxymethyl)ox:	0	0	0	0
(1,2,4-Trihydroxy-2,6,6-trimethylcyclohexyl)-3-bute:	0	0	0	0
-6-oxo-2,3-dihydropyran-3-yl] (2E,4E,6S)-8-hydroxy	1	0	0	0
-Trimethyl-11-oxabicyclo[8.2.1]trideca-1(13),4,7-trie	1	1	0	0
,5,8-trimethyl-11-oxatetracyclo[7.3.1.0~1,9~.0~3,7~	0	1	0	0
Oleanane -4H, + 2O	0	0	0	0
methoxyphenyl)prop-2-enoyl]oxy-2,5-bis(hydroxyme	0	0	0	1
Limonin	1	0	0	0
Puerarin	1	0	0	1
l)oxolan-2-yl]oxy-4,5-dihydroxy-6-(hydroxymethyl)(	0	0	0	0
Jatrorrhizine	0	0	0	0
Casticin	0	0	0	0
2',4'-Dihydroxychalcone	1	0	0	0
Arteannuin	1	0	0	1
Spermidine	0	0	0	0
thyl)-5,8a-dimethyl-2-methylidene-3,4,4a,6,7,8-hexal	0	0	0	0
Laetanine	1	0	1	1
Periplocymarin	1	1	1	1
Heptadecanoic acid	1	0	0	0
β-Gentiobiose	1	1	1	1
Homoorientin	1	1	1	1
catalposide	0	0	0	0
roxymethyl)oxolan-2-yl]oxy-3,4,5-trihydroxyoxan-2-	1	0	0	1
Desoxyrhaponticin	1	0	1	0
SINAPIC ACID	0	1	0	0
Sudan III	1	0	0	1
matairesinol	1	0	0	0
Aleuretic Acid	0	1	0	0
Glabrone	1	0	0	0
Ethyl gallate	0	0	1	0
R)-3,4,5-trihydroxy-6-(hydroxymethyl)oxan-2-yl]oxy	1	0	0	0

HYDROCORTISONE	1	1	0	1
3,19-Dihydroxyurs-12-ene-23,28-dioic acid	0	0	1	0
plumieride	0	0	0	1
Laudanoside	1	0	0	0
Sparteine	0	1	0	0
Rubiadin	1	0	0	0
3-Hydroxyurs-12-en-23-oic acid	0	1	1	0
ıranone, 5,6,7,7a-tetrahydro-6-hydroxy-4,4,7a-trimetl	0	1	0	0
Dopamine	1	0	0	1
ADENOSINE 3',5'-CYCLIC MONOPHOSPHATE	1	1	0	1
Glutamyltyrosine	1	1	1	1
Anisic aldehyde	0	1	1	0
Methyl pyrazine 2-carboxylate	0	1	0	1
-4-hydroxy-3,7-dimethylocta-2,6-dienoxy]-6-(hydrox	1	0	1	0
Homoserine	1	1	0	1
ıydroxy-6-(5-hydroxy-4-oxo-2-phenylchromen-7-yl)c	0	0	0	0
ydroxy-5-methylphenyl)methyl]-3,5-dihydroxy-4,4-d	0	1	0	0
4-(3-hydroxybutyl)-3,5,5-trimethylcyclohex-3-en-1-o	0	0	1	0
L-Anserine nitrate salt	1	1	1	0
Flavone base + 3O, C-Hex, C-Pen	1	0	0	1
Rhodinyl Acetate	1	1	0	0
Columbin	1	0	1	1
gamma-Dodecalactone	0	1	0	0
Corylin	0	0	0	1
Goniothalenol	0	0	1	0
Sophocarpine	0	1	0	0
(-)-Epicatechin gallate	1	0	0	0
ɔxy-6-[[(2S,3R,4S,5R)-3,4,5-trihydroxyoxan-2-yl]oxy	1	0	0	0
Harmaline	0	1	0	0
ɔxy-5-(hydroxymethyl)oxolan-2-yl]oxymethyl]-6-[(2I	1	0	0	0
3,3',4,4'-tetrahydroxylignan	1	0	0	0
3R,4R,5R,6S)-3,4,5-trihydroxy-6-methyloxan-2-yl]ox	0	0	0	1
Epigallocatechin	1	0	0	0
l (2R,3S)-3-(benzoylamino)-2-hydroxy-3-phenylprop	1	0	0	0
Glutamylphenylalanine	1	1	0	1
Hydroquinone	1	0	0	0
Tangeritin	1	0	0	0
Lobeline A	0	0	0	1
Curcurbitacin IIa	0	0	1	0
ıydroxy-3-(4-hydroxyphenyl)-3,4-dihydroisochromen	0	0	0	0
-(4-hydroxyphenyl)-6,8-bis(3,4,5-trihydroxyoxan-2-y	1	0	0	1
l-5-yl)propan-2-yl]-4,5-dimethoxy-2-prop-2-enylcycl	0	0	0	0
Alisol B	0	0	0	0
trifolirhizin	0	0	0	0
Lobaric Acid	1	0	0	1
Retrorsine	1	0	0	0
pyranosyl-(1->2)-[beta-D-xylopyranosyl-(1->6)]-beta	1	0	1	0
5S,6R)-3,4,5-trihydroxy-6-methyloxan-2-yl]oxy-2,3,4	0	0	1	0
3,9-Dimethoxypterocarpan	1	0	1	1
Neosperidin dihydrochalcone	1	0	0	0
Caudatin	0	1	0	0

Aconine	0	1	0	1
7-Methoxy-4-methylcoumarin	0	0	0	0
,3-Dihydroxy-3-methylbutoxy)furo(3,2-g)chromen-7-	0	0	0	0
3-(3,4-dihydroxyphenyl)-8-hydroxyisochromen-1-one	1	0	0	0
Jasminoside B	1	0	1	0
(-)-Syringaresinol di-O-glucoside	0	0	1	1
Methylparaben	0	0	1	0
xyphenyl)-6-[3,4,5-trihydroxy-6-(hydroxymethyl)oxa	1	1	1	1
4-O-Methylphloracetophenone	0	0	1	1
-tetramethyl-13-[(2-methylbutanoyl)oxy]-5-oxotetrac	1	0	0	0
Senkyunolide A	0	1	0	0
Neoandrographolide	0	1	0	0
hydroxyphenyl)-3,5,7-trihydroxy-6-methyl-2,3-dihyd	1	1	0	0
Pesticide3_Propoxur_C11H15NO3_Baygon	1	1	0	1
ydroxy-6-(hydroxymethyl)oxan-2-yl] 3,4,5-trimethox	1	0	1	1
5-O-Methylvisammioside	0	0	0	0
Levistilide A	0	1	1	1
Neriifolin	0	1	0	0
2,6-dihydroxy-4-methoxyphenyl)-3-phenylpropan-1-	0	0	0	0
zucapsaicin	0	0	1	0
Bruceine A	1	0	0	0
7,8-dihydromethysticin	0	0	1	0
3-deoxysappanchalcone	1	0	0	0
EPINEPHRINE	1	0	0	1
Kaempferol-7-O-rhamnoside	0	0	0	0
ydroxy-2-(2-hydroxy-4-methylphenyl)-6-methylhept-	0	1	0	0
3-Acetylaconitine	0	0	0	0
Procyanidin A2	0	0	1	0
Kainic Acid	0	0	0	0
Peimine	0	0	0	0
,6S)-6-(4-ethenylphenoxy)-3,4,5-trihydroxyoxan-2-yl	1	1	1	1
neoeriocitrin	0	0	0	0
4-Aminophenol	0	1	1	0
Tsugaric acid A	0	0	0	0
Rotenone	1	0	0	0
hoxycarbonyl-2-[(2S,3R,4S,5S,6R)-3,4,5-trihydroxy-	1	1	0	0
Nobiletin	1	1	1	1
Linderane	1	1	1	1
ostruthin	0	0	1	0
Berberine	1	0	0	1
Hydroxygenkwanin	1	0	0	1
Orcinol glucoside	0	0	0	0
2,6-di-tert-Butylphenol	0	1	0	0
Semilicoisoflavone B	1	0	0	0
Pseudoprotodioscin	1	0	0	0
xy-3-methoxyphenyl)-3,4-dimethyloxolan-2-yl]-2-m	0	0	1	0
Diosmetin-7-O-neohesperidoside	0	1	0	0
Bilobalide	0	0	0	0
aschantin	0	0	0	1
Kahweol	0	0	0	0
Tazettine	0	1	0	1

y-6-(hydroxymethyl)oxan-2-yl]oxyphenyl]-1,3,3a,4,6	1	0	1	0
ızyl 6-O-beta-D-glucopyranosyl-beta-D-glucopyranos	0	0	1	0
Gallic acid	1	0	0	0
Piperine	1	0	0	0
Indole-3-acetic acid	1	1	0	0
Tamoxifen citrate54965-24-1	0	0	0	0
Androsterone	1	1	1	1
Ginkgolide K	1	1	1	1
tetramethyl-1,4,4a,6,7,8-hexahydronaphthalen-1-yl)n	1	0	0	0
3,4,5-trimethoxycinnamic aldehyde	0	0	0	0
(+)-beta-Hydrastine	0	0	0	1
Methyl gallate	1	0	0	0
Sulfanilic acid	0	1	0	1
Biotin	0	1	0	0
maltotriose	0	0	1	0
CREATINE	1	0	0	1
Bicuculline	1	0	1	0
Coniine	1	1	1	0
3-Furfuryl 2-pyrrolecarboxylate	1	1	1	1
Dimethylcurcumin	1	0	1	1
Ethynylestradiol	1	0	1	0
4-Hydroxybenzoylcholine	1	0	0	1
Echimidine N-oxide	0	0	0	1
Methylxanthoxylin	1	0	0	0
abscisic acid	0	1	0	1
Epmedin B	1	0	0	1
genipin 1-gentiobioside	0	0	0	0
Methylnissolin-3-O-glucoside	0	0	1	0
3R,4S,5S,6R)-3,4,5-trihydroxy-6-(hydroxymethyl)ox	1	1	0	1
6,7,8-trimethoxychromen-2-one	0	0	0	0
Isosafrole	1	1	1	1
2,17-tetrahydroxy-16-methyl-2,4,14,19-tetraoxahexa	1	0	1	1
Isobavachin	1	0	1	0
Serine	0	1	0	1
3',4',7-Trihydroxyflavanone	1	1	1	1
y-3-methoxyphenyl)methyl]-3-(hydroxymethyl)oxola	1	0	1	1
Isopropyl 4-Hydroxybenzoate	1	1	0	1
icyclo[2.2.1]heptanyl) (E)-3-(4-hydroxy-3-methoxypl	0	0	0	0
Andrographolide	0	0	0	0
2,5-dihydroxy benzoic acid	0	0	0	1
5-en-2-yl)-4,4,8,10,14-pentamethyl-2,3,5,6,7,9,11,12,	0	0	0	1
5-Hydroxy-1-tetralone	0	1	0	0
Vinpocetine	0	0	1	0
xymethyl)oxan-2-yl]oxy-2,5a,6,6a-tetrahydro-1bH-ox	1	0	0	0
Xanyhyletin	0	0	1	0
,5-dimethoxyphenyl)-1,3,3a,4,6,6a-hexahydrofuro[3,4	0	0	1	0
acanthoside B	1	1	1	1
Cortodoxone	1	0	0	1
Lup-20(29)-en-3-ol, (3alpha)-	1	1	1	1
Celastrol	0	1	0	0
Delsoline509-18-2	0	1	1	0

Ranaconitine	0	0	0	0
DEOXYCARNITINE	0	1	0	1
Aurantio-obtusin	0	0	0	0
!-dimethyl-10-(2-methylbut-3-en-2-yl)pyrano[3,2-g]c	0	0	0	1
Diethyl phthalate	0	0	1	0
Pilosanone B	0	0	0	0
Carnitine (Dl)	1	0	1	1
Papaverine HCl	1	1	0	0
lomatin	0	0	0	0
(+/-)-Synephrine	1	1	0	1
Euphorbia factor L1	0	1	0	1
Chrysin Dimethyl Ether	0	1	0	1
Ganoderic acid G	0	0	0	0
6-Demethoxytangeretin	0	0	0	1
Murrayone	0	0	1	0
Sudan I	0	0	0	0
Flavanone base + 6O	1	0	0	0
Cimigenol-3-O-beta-D-xylpyranoside	0	1	0	0
lroxy-2,5-bis(hydroxymethyl)oxolan-2-yl]oxy-3,4,5-t	0	0	0	0
ɔan-2-yl)-1,4a-dimethyl-2,3,4,9,10,10a-hexahydrophe	1	0	0	1
5-Methoxyflavanone	1	1	0	0
9-(2-methoxy-2-oxoethyl)-4,8,10,12-tetramethyl-7-o	0	0	0	1
4-Nitrophenol	1	1	0	1
Nerylacetate	1	0	0	0
apogenol B base + O-HexA-Hex-Pen, O-C6H7O3(D	1	0	0	0
Isosakuranin	0	0	0	0
6-Shogaol	0	0	0	0
Prim-O-glucosylcimifugin	0	0	1	0
Heliotrine	0	1	0	1
Quebrachitol	0	0	1	0
Pantothenic acid (not validated)	1	0	1	1
2,6-Dimethoxyquinone	0	0	0	0
beta-Asarone	1	0	0	0
bakuchiol	0	0	0	1
Coniferaldehyde	1	0	1	1
Gallic acid trimethyl ether	1	1	1	1
Pramoxine	0	1	0	1
Grandisin	1	0	0	1
xo-8,9-dihydrofuro[2,3-h]chromen-8-yl)propan-2-yl a	1	1	1	1
Aloin	0	0	1	0
L-2,3-DIAMINOPROPIONIC ACID	1	1	1	1
syringin	1	0	1	1
Vanillic acid	0	0	1	0
Agarotetrol	1	0	1	1
Euphorbiasteroid	0	0	0	1
Epimedin A1	0	0	0	0
ɔxymethyl)-5,6-dimethyl-3,4,4a,5,6,7,8,8a-octahydro-	1	0	0	0
3-O-Acetyldiosgenin	0	1	1	1
ɣdroxy-6-(hydroxymethyl)oxan-2-yl]oxy})-4,9,15,20-	0	0	0	0
Aleuritic acid	0	0	0	0
Licoflavone A	0	1	1	1

Tulipinolide	1	1	1	1
Sabinene	1	1	1	1
ıoxyphenyl)-1,3,3a,4,6,6a-hexahydrofuro[3,4-c]furan-	1	0	0	1
(2S,3R,4S,5S,6R)-3,4,5-trihydroxy-6-(hydroxymethy	0	0	1	0
Papaverine	1	0	0	0
Bavachin	0	0	0	1
ıydroxymethyl)-6-[(E)-3-(4-hydroxyphenyl)prop-2-eı	1	0	0	0
9-Aminocamptothecin	0	0	0	0
ıyl-2-[(3E,7E)-4,8,12-trimethyl-3,7,11-tridecatrien-1-	0	1	0	0
Epimedin A	1	0	0	0
xanthohumol	0	0	0	0
Leucodin	0	0	0	1
AMINOADIPATE	0	1	0	0
Fexofenadine hydrochloride	0	1	0	1
ethyl-3-methylene-2-oxododecahydronaphtho[1,2-b]f	0	0	0	0
d, 5-[2-(2,5-dihydro-2-oxo-3-furanyl)ethyl]decahydrc	0	0	0	0
Hexylamine	1	1	1	1
IYDROXY-1-METHYLETHYL)-2,3-DIHYDRO-7F	0	0	0	0
Araloside A	0	0	1	0
ıethyl)-6-[4-(hydroxymethyl)-1-propan-2-ylcyclohex-	0	0	0	0
4-Hydroxycoumarin	1	0	1	0
Cyclo-Ile-Pro-diketopiperazine	0	1	0	1
Urocortisone	0	0	1	0
(-)-Cotinine	1	0	0	1
Dehydroandrographolide	0	0	0	0
decahydro-5-[(3Z)-5-hydroxy-3-methyl-3-penten-1-y	1	0	0	0
Vindoline	0	1	1	1
Carnosol	0	0	0	0
3-Deoxycaryoptinol	0	1	0	0
KOBUSONE	1	0	0	1
ʼl-2-oxo-9,10-dihydropyrano[2,3-f]chromen-10-yl) (Z	1	0	0	0
S-(-)-Carbidopa	1	0	1	0
Phloridzin	1	0	0	1
Lamiide	0	0	0	0
20-Hydroxyecdysone 20,22-acetonide	0	1	1	0
O-(6-deoxy-alpha-L-mannopyranosyl)-beta-D-glucop	1	0	1	0
alpinetin	1	0	0	0
Phenethylamine	1	1	1	1
isosakuranetin	0	0	0	0
decanedioic acid	0	1	1	0
Grayanotoxin I	1	0	0	0
Rubescensin A	0	0	0	0
Ginsenoside compound K	0	0	0	0
Poststerone	1	0	1	1
(-)-Asarinin	1	0	0	0
Solanidine (not validated)	0	0	0	0
ко-3-hexen-1-yl]-2-(beta-D-glucopyranosyloxy)-16-h	0	0	1	0
·oxy-4-{[(2E)-3-(4-hydroxyphenyl)-2-propenoyl]oxy	0	0	0	0
lroxy-3-methoxyphenyl)-6-(3-methylbut-2-enyl)-2,3-	1	0	0	1
3-(4-HYDROXYPHENYL)PYRUVATE	0	0	1	0
Pristimerin	0	0	0	0

17,17-pentamethyl-8-(prop-1-en-2-yl)pentacyclo[11.[illegible]	0	0	0	0
[illegible],3R,4S,5S,6R)-3,4,5-trihydroxy-6-(hydroxymethyl)o[illegible]	1	1	0	1
Fraxinol	1	0	0	0
Carnitine (Dl) Hydrochloride	0	0	0	0
Ginkgolide A	0	0	0	0
[illegible]l-2-[(2S,3R,4S,5S,6R)-3,4,5-trihydroxy-6-(hydroxym[illegible]	1	0	1	0
[illegible]ydroxymethyl)-2,2,6a,6b,9,12a-hexamethyl-1,3,4,5,6,[illegible]	0	0	1	0
Flavanone base + 4O, 2Prenyl	1	0	0	0
echinacoside	0	0	0	0
Ormosanine	1	1	1	1
Veratric acid	0	1	1	0
Picrotoxinin	1	0	0	1
Viridiflorine	0	0	0	1
1-[4-hydroxy-3-(3-methylbut-2-enyl)phenyl]ethanone	1	1	0	1
2-Chloro-DL-Phenylalanine	0	0	1	0

1 means that the ingredient was detected in the corresponding herb, 0 means that the ingredient was not detected in the corresponding herb

Supplement table 2. UHPLC-QE-MS Analysis negative ions

Compound name	yin yang huo	pu huang	da xue teng	pai jiang cao
2-amino-3-prop-2-enylsulfinylpropanoic acid	1	1	0	0
2,3-Dihydroxybenzoic acid	1	1	1	0
3-[(Carboxycarbonyl)amino]-L-alanine	0	0	1	0
3-Hydroxybenzaldehyde	0	0	0	0
Adenine	1	1	0	1
Apigenin	0	0	0	0
Benzoic acid	1	1	1	1
Chrysin	0	0	0	0
Coniferylaldehyde	1	0	0	0
Demethoxycurcumin	1	0	0	0
Deoxyadenosine	1	0	0	0
Ecgonine	0	0	0	1
Erythritol	0	1	0	0
Fumaric acid	0	0	0	1
HIPPURATE	0	1	0	0
Hydroxygenkwanin	0	0	0	0
Isobutyl 4-hydroxybenzoate	1	1	1	1
kojic acid	1	1	1	1
PHENYLACETIC ACID	1	1	1	1
Picroside III	0	0	0	0
Proline	1	1	0	1
PYRIDOXINE	1	1	0	1
Quassin	1	0	0	0
Spiculisporic acid	0	0	1	0
Threonic acid	1	0	0	0
Aspartate	0	1	1	1
Tectorigenin	1	0	0	1
Thymol	0	0	0	0
Jaceosidin	0	1	0	0
Glucose	0	0	0	0
Maleic acid	1	1	1	1
Quinic acid	1	0	1	1
[illegible]l)-5,9-dimethyltetracyclo[11.2.1.0^{1,10}.0^{4,9}]hex[illegible]	0	0	0	0
5,7-Dihydroxychromone	1	1	1	0
Demethylwedelolactone	0	0	0	0
Genistin	0	0	0	0
ISOPALMITIC ACID	0	0	1	0
arachidic acid	1	0	0	1
geniposide	0	0	0	1
Tridecanoic acid	0	0	0	0
Gentisic acid	1	1	1	1
Oleic acid	0	0	0	0
Isoleucine	0	0	0	0
4-HYDROXYBENZOATE	1	1	1	1
Sumaresinolic acid	0	0	1	0
Lauryl sulfate	0	0	0	0
Corosolic acid	1	0	1	1
Bergaptol	0	0	0	0
(+)-Epicatechin	1	1	1	1

Cholic acid	0	0	0	1
Rosmarinic acid	0	1	0	1
Carpachromene	1	0	0	0
Kaempferol	0	0	0	0
Arbutin	0	0	0	0
4-Nitrophenol	1	1	1	1
Palmitic acid	0	1	1	1
,11R,12aR)-1,10,11-trihydroxy-1,2,6a,6b,9,9	0	0	1	0
Lysionotin	0	0	0	0
aR)-1,10,11-trihydroxy-9-(hydroxymethyl)-2,	0	0	0	0
Erythronolactone	0	0	0	0
Chlorogenic acid	1	1	1	1
Lithocholic acid	0	0	0	0
Isorhamnetin	1	1	0	1
Galactose	1	0	1	0
Dihydromyricetin	1	0	0	0
syringaresinol	1	0	1	0
D-GLYCERIC ACID	1	1	1	1
Asiatic acid	0	0	1	0
p-Hydroxybenzaldehyde	0	0	0	0
Citric acid	1	1	1	1
Methylgallate	1	0	0	0
Methylsuccinic acid	1	1	1	1
Pinocembrin	1	1	0	1
Methyl eugenol	1	0	1	1
Sodium Deoxycholate	0	0	0	0
)-3,4,5-trihydroxy-6-(hydroxymethyl)oxan-2-	0	0	0	0
oxyethylidene]-6-[(2S,3R,4S,5S,6R)-3,4,5-tri	0	0	0	0
rihydroxy-6-(hydroxymethyl)oxan-2-yl]oxy-2	0	0	1	1
Phloridzin	1	0	1	1
Genistein	1	0	0	0
,5R)-2,3,6-trihydroxy-5,6-dimethylheptan-2-	0	0	1	0
Mannitol	0	0	0	0
isoimperatorin	1	0	0	0
Azelaic acid	0	1	0	0
13-HOTrE	1	1	0	0
sweroside	0	0	1	0
3-Methoxycinnamic acid	0	0	1	0
Methyl hexadecanoate	1	1	1	1
Betulinic acid	1	0	0	1
Succinic acid	1	1	1	1
2R,3R,4R,5R,6S)-3,4,5-trihydroxy-6-methyl	0	0	1	1
4-Aminophenol	0	0	0	1
Artesunate	0	1	0	0
trans-4-Coumaric acid	0	0	0	0
Curcumenol	1	1	1	1
Ginkgolic acid (C13:0)	0	0	0	1
Taxifolin	1	1	1	0
Lamiide	1	0	0	0
Roccellic Acid	1	1	0	0
Indolylmethyl glucosinolate + 1MeO	0	0	0	1

aR)-1,10,11-trihydroxy-9-(hydroxymethyl)-2,	0	0	0	1
Pimelic acid	1	0	0	0
Tauroursodeoxycholic acid	0	0	0	1
Homoorientin	0	0	0	0
Daidzein	1	0	0	0
Decanoic acid	1	0	0	0
-6-(4-ethenylphenoxy)-3,4,5-trihydroxyoxan-	1	0	0	0
7-Hydroxycoumarin	0	0	0	0
16-Hydroxyhexadecanoic acid	1	1	1	1
Eicosenoic acid	0	0	1	1
Abietic acid	0	1	0	0
Caffeic acid	1	1	1	1
4-Methylcatechol	0	0	0	0
Linoleic acid	1	1	1	1
6,7-Dihydroxycoumarin	1	1	0	1
Heptadecanoic acid	1	0	1	0
Hydroxyvalerenic Acid	1	1	1	1
Chenodeoxycholic acid	0	0	0	0
Ferulic Acid	1	0	1	1
Kaempferol 3-O-arabinoside	1	1	0	0
Naringenin chalcone	1	1	1	1
Hederagenin	1	0	1	1
5-Hydroxy-6,7-dimethoxyflavone	1	0	0	0
Anthranilic acid	0	0	1	0
,9,12a-heptamethyl-2,3,4,5,6,6a,7,8,8a,10,11,	0	0	0	1
osmanthuside H	0	0	1	1
Inermin	0	0	0	0
Gallocatechin	1	1	0	0
Quercetin-3-O-galactoside	0	0	0	1
3-phenyllactic acid	1	0	0	0
Fusidine	0	0	1	0
Aesculin	1	0	1	1
Phloretin-2'-O-glucoside	0	1	0	1
Phenylpyruvic acid	1	1	1	1
plumieride	1	0	0	0
Piceatannol	0	0	0	0
rhodioloside	1	1	1	1
Ginkgolic acid C17-1	0	0	0	1
Luteolin	1	0	0	1
Purpurin	0	0	0	0
FA 18:1+3O	1	1	1	1
Bayogenin	0	0	0	0
Myristic acid	0	0	0	0
3-O-Acetyl-16alpha-hydroxytrametenolic acic	0	0	1	0
5-Ethoxy-10-gingerol	0	0	0	0
Stearic acid	0	0	0	0
Psicose	0	0	0	0
hyl)-5,9-dimethyltetracyclo[11.2.1.$0^{1,10}.0^{4,9}$]h	1	1	1	1
(hydroxymethyl)oxan-2-yl]oxyphenyl]-1,3,3a	0	0	1	0
p-Coumaraldehyde	1	0	0	1
naringenin-7-O-glucoside	1	1	0	1

Procyanidin B1	1	0	1	0
Protocatechuic acid	1	1	1	1
rutamarin	0	0	1	1
coniferin	1	1	1	1
Luteolin-4'-O-glucoside	1	0	0	1
Tectochrysin	0	0	0	0
cirsimarin	0	0	0	1
Apigenin 7-O-glucuronide	0	0	0	0
Arabitol	1	0	0	0
Diosmetin	1	1	1	1
4-Hydroxyphenyllactic acid	1	1	0	1
(4-hydroxy-3-methoxyphenyl)methyl]butane-	0	0	0	0
Quercetin 3-O-beta-D-Glucuronide	1	1	0	0
syringin	0	0	0	1
Linolenic acid	0	0	0	0
Icariin	1	0	0	0
Pyrogallol	1	1	1	0
Dioscin	1	0	0	0
rhein	0	0	0	0
Honokiol	1	0	0	0
-(hydroxymethyl)oxan-2-yl]oxy-1,4a,5,6,7,7	1	1	0	1
picein	0	0	1	1
Tetrahydroxyxanthone	0	0	1	0
L-ARABITOL	0	0	0	0
Catechol	0	1	0	1
Quercetin	1	1	1	1
13-HODE	1	1	1	1
cordycepin	0	1	1	1
,4,5-trihydroxy-6-(hydroxymethyl)oxan-2-yl]	1	1	1	1
Eriodictyol	1	1	1	1
Ellagic acid	1	0	0	1
Piperonylic Acid	1	1	1	1
arctiin	0	0	0	0
MUCIC ACID	1	0	0	0
Medicagenic acid	0	0	1	0
Danshensu	1	1	0	1
Atractylenolide III	0	0	0	0
beta-Elemonic acid	0	0	0	0
Glomeratose A	0	1	1	0
L-Pyroglutamic acid	0	1	0	1
hoxyphenyl)prop-2-enoyl]oxy-2,5-bis(hydrox	1	0	0	1
FA 18:1+1O	1	1	1	1
Baicalein	0	0	0	0
Kaempferide	0	0	0	1
Uridine	0	1	1	1
Shikimic acid	1	0	1	0
thyl-4-[2-(5-oxo-2H-furan-3-yl)ethyl]-2,3,5,6	0	1	0	0
Adipic acid	1	1	1	1
Neohesperidin	1	1	0	1
Topotecan	1	0	0	0
D(-)-Salicin	1	1	1	1

Morusin	0	0	0	0
Schaftoside	1	0	0	1
1-O-b-D-glucopyranosyl sinapate	0	0	1	1
l-3-O-beta-glucopyranosyl-7-O-alpha-rhamnc	1	1	0	1
₹,3R)-2,3,6-trihydroxy-6-methylheptan-2-yl]-:	1	1	1	1
swertiamarin	0	0	1	0
1-Hydroxyanthraquinone	0	0	0	0
Eupafolin	1	0	0	1
Eriodictyol-7-O-glucoside	1	1	1	1
Diosmin	1	0	1	1
Nardosinone	0	0	0	0
Deoxycholic acid	0	0	0	0
Coumaroyl quinic acid	1	1	0	1
Jasmonic acid	1	1	1	0
Pedunculoside	0	0	1	1
Aconitic Acid	0	1	1	1
‹ymethyl)-1,2,6a,6b,9,12a-hexamethyl-2,3,4,5	0	0	1	0
Gallic acid	1	1	1	1
Antrapurol	0	0	0	0
4-hydroxyphenylacetic acid	0	1	0	1
Gentiopicroside	0	0	0	0
Sebacic acid	1	1	1	1
1,2-Dihydroxy anthraquinone	0	0	0	0
Malic acid	0	1	1	1
Benzene-1,2,4-triol	1	0	1	1
Phenylalanine	0	1	0	1
1,7-bis(4-hydroxyphenyl)heptane-3,5-diol	1	0	0	0
Madecassic acid	0	0	1	0
-dioxo-9H-pyrano[2,3-f]chromen-9-yl) (Z)-2-ı	0	0	0	1
Vicenin III	0	0	0	1
D-Gluconic acid	1	0	0	0
Acetosyringone	0	0	1	0
(-)-Catechin gallate	1	0	0	0
Dihydrokaempferol	1	0	1	0
;-trihydroxy-6-(hydroxymethyl)oxan-2-yl]oxy	0	1	1	1
suberosin	0	0	0	0
Acacetin-7-O-rutinoside	1	1	0	1
Diosmetin-7-O-beta-D-glucopyranoside	1	0	0	1
3-(2-HYDROXYPHENYL)PROPANOATE	0	0	0	1
ethoxyphenyl)-3,4,6,6a-tetrahydro-1H-furo[3,	0	1	0	1
Isomangiferin	0	0	1	0
Heneicosanoic acid	0	0	1	1
-2-yl]oxy-3,4,5-trihydroxyoxan-2-yl]methyl (	0	0	0	1
Norcimifugin	1	0	0	0
FA 18:2+1O	1	1	0	1
Isosakuranin	1	1	1	0
ıenyl)-6,8-bis[3,4,5-trihydroxy-6-(hydroxyme	0	0	0	1
Kaempferol-3-O-rhamnoside-7-O-rhamnoside	1	0	0	0
Enoxolone	0	0	1	0
Pelargonidin-3-O-glucoside	0	1	1	0
Salicylic acid	0	0	0	0

Hesperetin	1	1	0	0
Trilobatin	1	0	1	0
Isofraxidin	1	0	1	1
Sucrose	0	1	1	1
9-hydroxy-10,12-octadecadienoic acid	1	1	1	1
Bruceine A	1	0	1	0
Apigenin-7-O-glucoside	0	0	1	1
Sinigrin	0	0	0	0
6-Gingerol	0	1	1	1
3-O-Feruloylquinic acid	1	0	1	1
methyl palmitoleate	1	1	0	0
:oxymethyl)-6-[(E)-3-(4-hydroxyphenyl)prop-	0	1	0	0
licochalcone B	0	0	0	1
Orsellinic acid	0	0	0	0
Benzoic acid + 1O, O-Hex	0	1	0	1
Taurochenodeoxycholic Acid	0	0	0	1
yl)-6-[4-(hydroxymethyl)-1-propan-2-ylcyclo	0	0	0	0
Sinapoyl aldehyde	1	0	1	1
Phellodendrine chloride	1	0	0	0
3-Methylglutaric acid	1	1	0	1
Cinobufagin	0	0	0	0
Higenamine	1	0	0	0
Vaccenic acid	0	0	0	0
Tectoridin	1	0	0	0
Gluconapin (3-butenylglucosinolate)	0	0	0	1
),12a-heptamethyl-1,3,4,5,6,6a,7,8,8a,10,11,1	1	0	1	1
2-METHYLMALEATE	1	1	1	1
Isoscopoletin	0	0	0	0
-2-oxo-9,10-dihydropyrano[2,3-f]chromen-9-y	0	0	0	1
Hexose + C13H17O3	0	0	0	0
Trans-Cinnamic acid	1	0	0	0
Suberic acid	1	1	1	1
;,4S,5R,6R)-3,4,5-trihydroxy-6-methyloxan-2-	0	0	0	0
Jujuboside B	0	0	0	0
D-PANTOTHENIC ACID	1	1	1	1
Shanzhiside	1	1	1	1
Safflomin A	0	0	0	1
N-Acetyl-DL-glutamic acid	1	1	1	1
vanillic acid	1	1	1	1
'phenyl)-1,3,3a,4,6,6a-hexahydrofuro[3,4-c]fu	0	0	1	0
E)-3-(4-hydroxy-3-methoxyphenyl)prop-2-eno	1	0	1	1
Ajugol	0	0	0	1
Poricoic acid A	0	1	0	1
loganic acid	0	0	1	0
Linarin	0	1	1	1
Oenin	1	1	0	1
Phenyl-beta-glucopyranoside	0	1	0	0
Ginkgolic Acid C15:1	0	0	0	0
9-HODE	1	1	0	1
Shanzhiside methyl ester	1	0	0	0
Glabrolide	1	1	0	1

ıethyl)-1,2,6a,6b,9,12a-hexamethyl-2,3,4,5,6,6	0	0	1	1
Typhaneoside	1	1	0	0
Oleanoic Acid	0	0	0	1
Asperulosidic acid	1	1	1	1
Lignoceric acid	0	0	1	1
cirsimaritin	1	0	0	0
Mulberrin	1	0	0	0
Hyperoside	1	0	0	0
Prostaglandin E1	0	1	0	1
S,5S,6R)-3,4,5-trihydroxy-6-(hydroxymethyl)	0	0	0	0
Chaulmoogric Acid	1	1	1	1
Maltopentaose	0	0	0	0
Limonin	1	0	1	0
Harpagoside	0	0	1	0
ı-2-yl)-4,4,8,10,14-pentamethyl-2,3,5,6,7,9,11	0	0	0	0
Naringin	1	1	1	0
Flavonol base + 3O, O-Hex	1	1	1	1
Emodin	1	0	0	1
kaempferol 7-neohesperidoside	1	1	0	1
-5-[(E)-3-(4-hydroxyphenyl)prop-2-enoyl]ox	1	0	1	1
1a,2,3,4a,5,6,7a,7b-octahydrocyclopropa[h]az	0	0	0	0
matairesinol	1	0	0	1
aucubin	1	0	1	0
Echinocystic acid	0	0	1	0
Acetyl-11-keto-beta-boswellic acid	0	0	0	0
(EZ)-sinapic acid	0	0	1	1
Propyl gallate	1	0	1	0
Eleutheroside E	1	0	1	1
Polygalic acid	0	0	1	0
LPC 16:0	0	0	0	0
Tricosanoic acid	0	0	1	1
Glutamyltyrosine	1	1	1	1
Manninotriose	0	1	0	1
MGMG 18:2	0	1	0	0
Glutamylphenylalanine	0	0	0	0
oleuropein	0	0	0	0
Helicid	1	0	1	0
Piscidic Acid	1	1	1	1
Baohuoside I	1	0	0	0
Luteolin 7-glucuronide	1	1	0	0
18-Beta-glycyrrhetinic acid	1	0	1	0
3,4,5-trihydroxy-6-[[(2S,3R,4S,5S)-3,4,5-trih	0	1	0	1
ihydroxy-6-(hydroxymethyl)oxan-2-yl]oxybu	0	0	1	0
Petunidin-3-O-beta-glucopyranoside	1	0	0	0
oxy-6-(5-hydroxy-4-oxo-2-phenylchromen-7-	1	0	0	0
Dihydroartemisinin	0	0	0	0
y-3-methoxyphenyl)-6-(3-methylbut-2-enyl)-	1	0	0	1
Orcinol gentiobioside	1	0	1	1
y-6-[[(E)-3-(4-hydroxy-3,5-dimethoxyphenyl	0	0	1	0
Palmitoleic acid	1	0	0	1
Nicotinic acid	1	1	0	1

trans-Zeatin-riboside	1	0	0	1
Glabranine	1	0	1	0
Phytolaccagenin	0	0	0	0
Aloenin	0	0	0	0
7-Hydroxy-4-methylcoumarin	0	1	0	0
6-[[(2S,3R,4S,5R)-3,4,5-trihydroxyoxan-2-yl]	1	0	1	0
Ginsenoside Ro	0	0	1	0
dehydroabietic acid	1	0	0	0
Pinoresinol 4-O-glucoside	0	0	1	0
5-(4-hydroxyphenoxy)oxan-2-yl]methyl (E)-3	0	1	0	1
xanthohumol	1	1	1	1
D-sorbosonic acid	0	0	0	0
3,4,5-trimethoxycinnamic acid	1	0	0	0
Desmethylxanthohumol	1	1	1	1
y-2,5-bis(hydroxymethyl)oxolan-2-yl]oxy-3,4	0	1	0	0
2-Methoxy-4-vinylphenol	1	0	0	1
Lactose	0	0	0	0
Melibiose	0	0	1	0
Santonin	0	0	0	0
Ethyl ferulate	1	0	0	0
ydroxy-4-(hydroxymethyl)oxolan-2-yl]oxyme	1	0	1	0
columbianetin	0	0	0	0
DGMG 18:3	1	1	1	1
Nystose	0	0	0	0
roxyphenyl)-3,5,7-trihydroxy-6-methyl-2,3-di	0	1	0	0
ARTEMISIN	0	0	0	1
Raffinose	1	0	1	0
Bufalin	0	0	0	0
Isoschaftoside	0	0	0	0
Pectolinarigenin	0	0	0	0
2,6a,6b,9,9,12a-heptamethyl-2,3,4,5,6,6a,7,8,8	1	0	1	1
Benzoic acid + 2O, O-Hex	0	1	0	1
Brazilin	1	0	0	1
Harpagide	1	0	1	0
17,17-pentamethyl-3,6,16-trioxapentacyclo[9.	0	0	1	0
Atractyloside A	0	0	0	0
Caudatin	0	1	0	0
Aurantio-obtusin beta-D-glucoside	1	0	0	1
3,4-Dihydroxyphenylethanol	1	0	1	0
Isoliquiritigenin	0	0	0	0
Arachidonic acid	0	0	0	0
Vicenin II	1	0	0	0
Isopeonol	1	0	1	0
L-Glutamic acid	0	1	0	0
Kaempferitrin	1	1	1	0
Blinin	0	1	0	0
Rhamnetin	1	0	0	0
Casticin	0	0	0	1
Grosvenorine	1	1	0	1
Methyl vanillate	1	0	0	1
Apigenin-8-C-glucoside	0	0	0	0

Digoxin	0	0	1	0
.3R,4S,5S,6R)-3,4,5-trihydroxy-6-(hydroxyme	0	0	1	0
Cryptochlorogenic acid	1	0	1	1
R,4S,5S,6R)-3,4,5-trihydroxy-6-(hydroxymet	0	0	0	0
Androsin	0	1	0	0
5-pentahydroxy-2,6,14,17-tetramethyl-10-oxa	0	1	0	1
Dihydrocucurbitacin F	0	0	1	0
Irigenin	0	1	0	1
Nervonic acid	0	0	1	1
ıydroxyphenyl)-6,8-bis(3,4,5-trihydroxyoxan-	1	0	0	1
Tenuifoliside B	0	0	1	0
Flavanomarein	1	1	1	0
ESTRIOL	1	0	1	1
Benzyl alcohol + Hex-Pen	1	1	1	0
DIGOXIGENIN	0	0	0	0
›xymethyl)-2,2,6a,6b,9,12a-hexamethyl-1,3,4,	0	0	0	0
:etin-3-O-beta-D-glucose-7-O-beta-D-gentiob	0	0	0	1
bilobol	0	0	0	0
-3-yloxy]-6-[[(2S,3R,4R)-3,4-dihydroxy-4-(hy	1	0	1	1
›xymethyl)-2,2,6a,6b,9,12a-hexamethyl-1,3,4,	0	0	1	0
11b-hexahydro-1H-benzo[a]quinolizin-2-yl]m	1	1	1	1
Chrysanthemic Acid	0	0	0	1
Cichoric acid	0	0	0	1
xo-2,3-dihydropyran-3-yl] (2E,4E,6S)-8-hydr	0	1	0	0
Bruceine D	1	0	0	1
Naringenin	1	0	0	0
Lobetyolin	0	0	0	0
Ailanthone	0	0	0	0
Glycocholic acid	0	0	0	0
Catalpol	1	1	1	1
/-6-(hydroxymethyl)oxan-2-yl]oxyoxan-2-yl](	0	0	1	1
Morroniside	0	0	1	0
3-Hydroxykynurenine	1	1	0	0
Phenylacetic acid + 2O, O-Hex	1	0	1	1
Scopolin	1	0	0	1
Cianidanol	1	0	0	0
Complanatuside	0	0	1	1
eudesmin	1	0	0	0
9-Hydroxy-10,12,15-octadecatrienoic acid	1	1	1	1
Pygenic acid A b	0	0	1	0
Tsugaric acid A	1	0	1	0
Xanthyletin	0	0	0	0
isosakuranetin-7-O-neohesperidoside	0	0	1	0
Chrysophanol 8-O-beta-D-glucoside	0	0	0	1
Benzyl glucosinolate	0	0	0	1
2-(4-hydroxyphenyl)-8-(3-methylbut-2-enyl)-2	1	0	0	0
Tyrosine	1	1	0	1
Pentose-Hexose + C10H17	0	0	1	0
Deoxyloganic acid	0	0	0	0
Triptophenolide	0	0	0	0
/methyl)oxolan-2-yl]oxy-3,4,5-trihydroxyoxa	1	0	0	1

Isocitrate	1	0	1	0
Eurycomalactone	0	1	0	1
ethoxyphenyl)propan-2-yl]oxy-6-(hydroxyme	1	1	0	1
phellopterin	1	1	0	0
Bayogenin base + O-Hex-Hex	0	0	1	0
1,3-Dicaffeoylquinic acid	1	0	0	0
),9,13,20-tetramethyl-4,8,15,18-tetraoxahexac	0	0	0	0
Gallic acid hexoside	1	1	1	0
Verbascoside	1	0	0	1
Di-n-butyl phthalate	1	1	1	1
oxy-2-(4-hydroxyphenyl)-7-methoxy-2,3-dih	0	0	1	1
xy-6-methyl-2-(3,4,5-trihydroxyphenyl)-2,3-c	1	1	1	0
P-Anisic acid	1	1	1	1
,2,6a,6b,9,9,12a-heptamethyl-1,3,4,5,6,6a,7,8,	0	0	0	0
Strophanthidin	0	1	0	0
Genipin 1-O-beta-D-gentiobioside	1	1	0	0
Agaric Acid	0	1	0	0
Rutin	1	1	1	1
ıydroxy-6-(hydroxymethyl)oxan-2-yl]-8-[(2S,	0	0	0	1
Grandisin	0	0	0	0
Quillaic acid	1	0	0	0
rihydroxy-6-[[(2S,3R,4S,5R)-3,4,5-trihydroxy	0	0	0	0
(E)-3-(4-hydroxy-3-methoxyphenyl)prop-2-en	0	0	0	0
Paeonolide	1	0	1	1
Tiliroside	1	0	0	1
Methylophiopogonanone A	1	0	0	0
Citraconic acid	0	0	0	0
18alpha-glycyrrhetinic acid	0	0	1	0
Coixol	0	1	0	1
8-tetramethyl-4-oxo-2,3-dihydropyrano[2,3-h]	0	0	0	0
asperuloside	1	0	0	0
Pseudolaric acid C	0	1	0	1
Protolichesterinic acid	0	1	1	1
Thioctic acid	1	1	1	1
Orientin	1	1	0	1
ydroxyphenyl)prop-2-enoyl]oxy}-1,3,5-trihyd	0	0	0	0
Amygdalin	1	0	0	1
Pechueloic Acid	0	0	0	0
Corilagin	0	0	0	1
ASCORBIC ACID	1	0	0	0
Isorhynchophylline	0	1	1	0
LEVODOPA	1	1	0	1
L-Tryptophan	1	1	0	1
,4S)-2,2,4-trimethyl-3-oxabicyclo[2.2.2]octan	0	0	0	1
Phloretin	1	1	0	0
Gentiopicrin	0	0	1	1
Dihydroartemisinic acid	1	1	1	1
-trimethyl-4,5a,6,8,8a,9-hexahydro-1H-azuler	0	1	1	0
FA 9:1+1O	0	1	1	0
Dehydrotrametenolic acid	1	0	1	1
7,8-Dihydroxycoumarin	1	0	0	0

Spinosin	1	0	0	0
Dihydrocoumaroyl Hexoside	1	1	1	1
Potassium 7-hydroxy-1-naphthalenesulfonate	0	0	0	0
Resibufogenin	0	0	0	0
5,9,9,13,19,20-heptamethyl-24-oxahexacyclo	0	1	0	1
Kawain	0	0	0	0
:y-4-(hydroxymethyl)oxolan-2-yl]oxy-2-(hydı	0	0	0	0
Forsythoside E	1	0	1	0
Biotin	0	0	0	0
12:4+3O fatty acyl hexoside	1	0	0	0
6"-O-Acetylglycitin	1	0	1	0
Afzelin	1	0	0	1
Embelin	0	0	0	0
Epigallocatechin	1	0	0	0
trifolirhizin	0	0	0	0
Loganin	0	0	0	0
Icaritin	1	0	0	0
Syringic Acid	0	0	1	1
5Alpha-Cholestanol	0	0	0	0
Mesaconic acid	1	1	1	1
nethyl-10-(2-methylbut-3-en-2-yl)pyrano[3,2-	1	0	0	0
5-Hydroxytryptophan	0	1	0	1
Noreugenin	0	0	0	1
1-O-trans-cinnamoyl-beta-D-glucopyranose	0	0	1	1
Paracetamol	0	0	1	0
ıethyl)oxan-2-yl]oxy-2,5a,6,6a-tetrahydro-1bI	0	0	0	0
3-(4-Hydroxyphenyl)-1-propanol	0	1	1	0
Usnic acid	1	0	1	1
Diffractic acid	0	0	1	1
Diethyl-phthalate	1	1	0	0
Pectolinarin	0	0	0	0
Leucoside	1	1	1	0
Saikosaponin D	1	0	0	0
Narcissoside	0	1	0	0
Geranic acid	0	0	0	0
DIMETHYLCAFFEIC ACID	0	0	0	0
4-Methoxysalicylic acid	1	1	1	1
Geniposidic acid	1	0	1	1
:hylhept-3-en-2-yl]-6,10-dimethyl-16,17-diox:	0	0	0	0
Ginkgolide A	0	0	0	0
Fraxetin	0	0	0	0
Alisol A 24-acetate	0	0	0	1
-7-propan-2-yl-2,3,4,4b,5,6,10,10a-octahydro	0	0	0	1
4S,5S,6R)-3,4,5-trihydroxy-6-(hydroxymethy	0	0	1	0
l-dihydroxyphenyl)prop-2-enoyl]oxy-1,4,5-tri	1	0	1	1
ALPHA-AMINOADIPATE	0	1	0	0
Podophyllin Acetate	1	0	0	0
ethoxyphenyl)-4-(hydroxymethyl)oxolan-3-yl	0	1	1	1
dihydroxyphenyl)-3,7-dihydroxy-2,3-dihydro	1	0	1	0
xy-4-prop-2-enylphenoxy)-1-hydroxypropyl]-	1	0	1	1
ydroxy-1,4a-dimethyl-2-oxo-7-propan-2-yl-1(	0	1	0	1

catalposide	0	0	0	1
Picrotin	0	0	0	0
Acacetin-7-O-neohesperidoside	0	0	1	0
Verbenalin	1	1	1	1
oxymethyl)-2,2,6a,6b,9,12a-hexamethyl-1,3,4,	0	0	0	0
3,4-Dicaffeoylquinic acids	1	0	1	1
Aleuritic acid	0	1	0	0
Maltotriose	0	0	0	0
Orcinol glucoside	0	0	1	1
8a-dimethyl-2-methylidene-3,4,4a,6,7,8-hexal	1	1	0	1
,4S,5S,6R)-3,4,5-trihydroxy-6-(hydroxymethy	0	0	0	0
oxy]-3,5-bis({[(2E)-3-(3,4-dihydroxyphenyl)p	0	0	0	0
H)-one, 4,4a,5,6,7,7a,8,9-octahydro-3,4,8-tril	0	1	0	1
Iristectorigenin B	1	0	0	1
Kainic acid	1	0	0	0
Soybean saponin fraction B1	1	0	0	1
2-enyl)-7-[(2S,3R,4S,5S,6R)-3,4,5-trihydroxy	1	0	0	0
Coumaroyl Hexoside	0	0	0	0
Asperosaponin VI	0	0	0	0
(+)-Cannabidiol	0	0	0	0
Isovitexin	1	0	0	1
isorhapontigenin	0	0	0	0
Inositol	1	1	1	1
Alisol A	0	0	0	0
Rhizonic acid	1	1	0	0
Sinapoylhexoside	1	0	1	1
4-dihydro-3-(2-hydroxyethylidene)-5-(methox	1	0	1	1
a-tetradecahydropicen-3-yl]oxy]-5-[(2S,3R,4S	0	1	0	0
Epmedin C	1	0	0	0
Aleuretic Acid	0	1	0	0
(3aS,6Z,10E,11aR)-10-methyl-3-methylidene	0	0	0	1
oxymethyl)-6-[4-[(E)-3-hydroxyprop-1-enyl]]	0	1	0	1
Isoanhydroicaritin	1	0	0	1
Oxypaeoniflorin	1	0	0	0
Sibiricose A5	0	1	0	1
Austricine	0	1	0	1
3-Deoxycaryoptinol	1	0	0	1
Behenic acid	1	0	0	0
Sec-O-Glucosylhamaudol	1	0	0	0
17-pentamethyl-8-(prop-1-en-2-yl)pentacyclo[	0	0	1	1
-3,4,5-trihydroxy-6-(hydroxymethyl)oxan-2-y	0	0	1	0
L(+)-Arginine	0	1	1	1
Eupatilin	0	0	0	1
Isoguanosine	1	1	1	1
Disinapoyl Hexoside	1	0	0	1
neoeriocitrin	1	0	1	1
3-Hydroxybutyric acid	0	0	0	0
Carnosol	0	0	0	0
Vanillin	0	0	0	1
calceolarioside A	0	1	1	0
Glutathione	1	0	0	0

,3R,4R)-3,4-dihydroxy-4-(hydroxymethyl)tetr	0	0	0	1
8-Desoxygartanin	1	1	1	1
Paeoniflorin	1	0	1	1
(-)-Syringaresinol di-O-glucoside	1	0	0	0
Wedelolactone	1	0	0	1
Hematoxylin	0	0	0	0
Methyl 3,4,5-trimethoxycinnamate	0	0	0	1
)-5,8a-dimethyl-2-methylidene-3,4,4a,6,7,8-h	0	0	1	0
Dihydrocapsaicin	1	1	1	1
Pentadecanoic acid	0	0	0	1
10-Deacetylbaccatin III	0	1	0	0
Bilobalide	0	0	0	0
Quercetin-3-Arabinoside	1	1	0	1
3,4-di-O-caffeoylquinic acid	1	0	1	0
phenyl)-3,5-dihydroxy-3,4-dihydro-2H-chrom	0	0	0	0
Dihydroresveratrol	1	1	0	1
Flemiphilippinin A	1	0	0	0
Ingenol	1	0	0	1
alternariol monomethyl ether	0	0	0	0
Forskolin	1	0	0	1
Procyanidin B2	0	0	0	0
Astringin	0	0	1	1
Taxifolin 7-rhamnoside	0	0	0	0
Stachyose	0	0	1	0
3-coumaric acid	1	0	1	1
Didymin	0	0	1	1
Rotenone	1	1	1	1
Valine	0	0	0	0
Isoliquiritin	0	0	0	0
Tagatose	0	0	0	0
Docosahexanoic acid	0	0	0	0
Prulaurasin	1	1	1	1
3,4-Dihydroxyphenylalanine	0	1	0	0
Coumarin base + 1O, 1MeO, O-Hex	1	1	1	1
Oxyresveratrol	0	0	0	0
(+/-)-Jasmonic acid	0	0	0	1
9(S)-HOTrE	1	1	1	1
S,5S,6R)-3,4,5-trihydroxy-6-(hydroxymethyl)	1	0	1	0
Cefalexin	1	1	1	0
Quercetin-3-O-glucose-6"-acetate	1	1	1	1
Isopropyl 4-Hydroxybenzoate	1	1	0	0
Isorhamnetin-3-O-nehesperidine	1	1	0	1
Camelliaside B	0	1	0	0
xymethyl)-5,6-dimethyl-3,4,4a,6,7,8-hexahyc	1	0	0	1
Kushenol F	1	0	1	0
Gossypetin-8-C-glucoside	0	0	0	0
Azadirachtin	1	0	0	0
Procyanidin A2	0	1	1	0
Octyl gallate	0	0	0	0
gamma-mangostin	0	1	1	0
9-HOTrE	0	1	0	0

Cyasterone	0	1	0	0
Peucedanol	0	0	0	1
⁴R,5R,6S)-3,4,5-trihydroxy-6-methyloxan-2-y	0	0	0	1
D(-)-Gulono-gamma-lactone	1	1	1	1
17-Hydroxyisolathyrol	1	0	0	0
Gedunin	1	0	0	0
hydroxyphenyl)ethoxy]-3,5-dihydroxy-6-(hyd	1	0	1	1
13-alpha-(21)-Epoxyeurycomanone	1	1	0	0
Pristimerin	0	1	0	0
abscisic acid	0	0	1	1
PAEDEROSIDE	1	0	0	0
Morellic acid	0	1	0	1
Chikusetsusaponin IV	1	0	0	0
Gibberellic acid	0	0	1	1
Capsaicin	1	1	0	1
hirsutanone	1	0	0	0
Farrerol	1	0	0	0
Momordin Ic	0	0	1	0
Epicatechin	1	0	0	0
methoxyphenyl)methyl]-3-(hydroxymethyl)o	1	0	1	1
Kukoamine B	0	0	0	0
VERATRIC ACID	1	0	0	0
Tenuifoliside A	0	0	1	0
nethyl-10-(3,4,5-trihydroxyoxan-2-yl)oxy-1,3,	1	0	1	1
Xylose	0	1	0	0
dihydroferulic acid	1	1	1	1
Rhaponticin	0	0	0	0
Phenethyl caffeate	0	0	0	1
oxy-α-L-mannopyranosyl-(1->4)-[β-D-glucop	0	1	0	0
-(beta-D-glucopyranosyloxy)-3,4-dihydro-, al	0	1	0	1
Hispidulin	0	0	0	0
Syringetin-3-O-galactoside	1	1	0	0
Puerarin	0	0	0	0
Hastatoside	1	0	0	0
2-Chloro-DL-Phenylalanine	1	1	1	1

1 means that the ingredient was detected in the corresponding herb, 0 means that the ingredient was not detected in the corresponding herb

Supplement table 3. Compounds in HYJDF with OB > 30% and DL > 0.18.

No	MOL ID	Herbal Compound	OB	DL	Herb
1	MOL000098	quercetin	46.43	0.28	EBM/PS/TAL
2	MOL000422	kaempferol	41.88	0.24	EBM/PS/TAL
3	MOL000359	sitosterol	36.91	0.75	EBM/PS/SC
4	MOL000358	beta-sitosterol	36.91	0.75	PS/SC/TAL
5	MOL000006	luteolin	36.16	0.25	EBM/PS
6	MOL001510	24-epicampesterol	37.58	0.71	EBM
7	MOL001645	Linoleyl acetate	42.1	0.2	EBM
8	MOL001771	poriferast-5-en-3beta-ol	36.91	0.75	EBM
9	MOL001792	DFV	32.76	0.18	EBM
10	MOL003044	Chryseriol	35.85	0.27	EBM
11	MOL003542	8-Isopentenyl-kaempferol	38.04	0.39	EBM
12	MOL004367	olivil	62.23	0.41	EBM
13	MOL004373	Anhydroicaritin	45.41	0.44	EBM
14	MOL004380	C-Homoerythrinan, 1,6-didehydro-3,15,16-trimethoxy-, (3.beta.)-	39.14	0.49	EBM
15	MOL004382	Yinyanghuo A	56.96	0.77	EBM
16	MOL004384	Yinyanghuo C	45.67	0.5	EBM
17	MOL004386	Yinyanghuo E	51.63	0.55	EBM
18	MOL004388	6-hydroxy-11,12-dimethoxy-2,2-dimethyl-1,8-dioxo-2,3,4,8-tetrahydro-1H-isochromeno[3,4-h]isoquinolin-2-ium	60.64	0.66	EBM
19	MOL004391	8-(3-methylbut-2-enyl)-2-TALenyl-chromone	48.54	0.25	EBM
20	MOL004394	Anhydroicaritin-3-O-alTALa-L-rhamnoside	41.58	0.61	EBM
21	MOL004396	1,2-bis(4-hydroxy-3-methoxyTALenyl)propan-1,3-diol	52.31	0.22	EBM
22	MOL004425	Icariin	41.58	0.61	EBM
23	MOL004427	Icariside A7	31.91	0.86	EBM
24	MOL000622	Magnograndiolide	63.71	0.19	EBM
25	MOL001677	asperglaucide	58.02	0.52	PS
26	MOL001678	bolusanthol B	39.94	0.41	PS
27	MOL001790	Linarin	39.84	0.71	PS
28	MOL001689	acacetin	34.97	0.24	PS
29	MOL002322	isovitexin	31.29	0.72	PS
30	MOL001697	Sinoacutine	63.39	0.53	PS
31	MOL000449	Stigmasterol	43.83	0.76	PS
32	MOL007923	2-(4-hydroxyTALenyl)ethyl (E)-3-(4-hydroxyTALenyl)prop-2-enoate	93.36	0.21	SC
33	MOL000096	(-)-catechin	49.68	0.24	SC
34	MOL001040	(2R)-5,7-dihydroxy-2-(4-hydroxyTALenyl)chroman-4-one	42.36	0.21	TAL
35	MOL001439	arachidonic acid	45.57	0.2	TAL
36	MOL000354	isorhamnetin	49.6	0.31	TAL

Supplement table 4. EM related genes targeted by active compound of HYJDF.

No.	UniProtKB	Gene Symbol	Protein Names	Status
1	O15439	ABCC4	ATP-binding cassette sub-family C member 4	reviewed
2	Q9UNQ0	ABCG2	ad substrate specificity ATP-binding cassette transporter ABC	reviewed
3	P35869	AHR	Aryl hydrocarbon receptor	reviewed
4	P15121	AKR1B1	Aldo-keto reductase family 1 member B1	reviewed
5	P42330	AKR1C3	Aldo-keto reductase family 1 member C3	reviewed
6	P31749	AKT1	RAC-alpha serine/threonine-protein kinase	reviewed
7	P09917	ALOX5	Polyunsaturated fatty acid 5-lipoxygenase	reviewed
8	P10275	AR	Androgen receptor	reviewed
9	Q07812	BAX	Apoptosis regulator BAX	reviewed
10	P10415	BCL2	Apoptosis regulator Bcl-2	reviewed
11	Q07817	BCL2L1	Bcl-2-like protein 1	reviewed
12	O15392	BIRC5	Baculoviral IAP repeat-containing protein 5	reviewed
13	P42574	CASP3	Caspase-3	reviewed
14	Q14790	CASP8	Caspase-8	reviewed
15	P55211	CASP9	Caspase-9	reviewed
16	P13500	CCL2	C-C motif chemokine 2	reviewed
17	P20248	CCNA2	Cyclin-A2	reviewed
18	P14635	CCNB1	G2/mitotic-specific cyclin-B1	reviewed
19	P24385	CCND1	G1/S-specific cyclin-D1	reviewed
20	P29965	CD40LG	CD40 ligand	reviewed
21	P38936	CDKN1A	Cyclin-dependent kinase inhibitor 1	reviewed
22	P42771	CDKN2A	Cyclin-dependent kinase inhibitor 2A	reviewed
23	P11597	CETP	Cholesteryl ester transfer protein	reviewed
24	P36544	CHRNA7	Neuronal acetylcholine receptor subunit alpha-7	reviewed
25	O15111	CHUK	Inhibitor of nuclear factor kappa-B kinase subunit alpha	reviewed
26	O14493	CLDN4	Claudin-4	reviewed
27	P02452	COL1A1	Collagen alpha-1	reviewed
28	P02741	CRP	C-reactive protein [Cleaved into: C-reactive protein	reviewed
29	P07339	CTSD	Cathepsin D	reviewed
30	P02778	CXCL10	C-X-C motif chemokine 10	reviewed
31	O14625	CXCL11	C-X-C motif chemokine 11	reviewed
32	P10145	CXCL8	Interleukin-8	reviewed
33	P11511	CYP19A1	Aromatase	reviewed
34	P04798	CYP1A1	Cytochrome P450 1A1	reviewed

35	P05177	CYP1A2	Cytochrome P450 1A2	reviewed
36	Q16678	CYP1B1	Cytochrome P450 1B1	reviewed
37	P08684	CYP3A4	Cytochrome P450 3A4	reviewed
38	P27487	DPP4	Dipeptidyl peptidase 4	reviewed
39	P14416	DRD2	Dopamine receptor D2	reviewed
40	P01133	EGF	Pro-epidermal growth factor	reviewed
41	P00533	EGFR	Epidermal growth factor receptor	reviewed
42	P56537	EIF6	Eukaryotic translation initiation factor 6	reviewed
43	P19419	ELK1	ETS domain-containing protein Elk-1	reviewed
44	P04626	ERBB2	Receptor tyrosine-protein kinase erbB-2	reviewed
45	P03372	ESR1	Estrogen receptor	reviewed
46	Q92731	ESR2	Estrogen receptor beta	reviewed
47	P00734	F2	Prothrombin	reviewed
48	P13726	F3	Tissue factor	reviewed
49	P48023	FASLG	Tumor necrosis factor ligand superfamily member 6	reviewed
50	P01100	FOS	Proto-oncogene c-Fos	reviewed
51	P17302	GJA1	Gap junction alpha-1 protein	reviewed
52	P49841	GSK3B	Glycogen synthase kinase-3 beta	reviewed
53	P09488	GSTM1	Glutathione S-transferase Mu 1	reviewed
54	P09211	GSTP1	Glutathione S-transferase P	reviewed
55	Q16665	HIF1A	Hypoxia-inducible factor 1-alpha	reviewed
56	P09601	HMOX1	Heme oxygenase 1	reviewed
57	P05362	ICAM1	Intercellular adhesion molecule 1	reviewed
58	P01579	IFNG	Interferon gamma	reviewed
59	P01344	IGF2	Insulin-like growth factor II	reviewed
60	P17936	IGFBP3	Insulin-like growth factor-binding protein 3	reviewed
61	P22301	IL10	Interleukin-10	reviewed
62	P01583	IL1A	Interleukin-1 alpha	reviewed
63	P01584	IL1B	Interleukin-1 beta	reviewed
64	P60568	IL2	Interleukin-2	reviewed
65	P05112	IL4	Interleukin-4	reviewed
66	P05231	IL6	Interleukin-6	reviewed
67	P05412	JUN	Transcription factor AP-1	reviewed
68	P35968	KDR	Vascular endothelial growth factor receptor 2	reviewed
69	P11137	MAP2	Microtubule-associated protein 2	reviewed
70	P28482	MAPK1	Mitogen-activated protein kinase 1	reviewed

71	Q16539	MAPK14	Mitogen-activated protein kinase 14	reviewed
72	P45983	MAPK8	Mitogen-activated protein kinase 8	reviewed
73	Q07820	MCL1	Induced myeloid leukemia cell differentiation protein Mcl-1	reviewed
74	Q00987	MDM2	E3 ubiquitin-protein ligase Mdm2	reviewed
75	P08581	MET	Hepatocyte growth factor receptor	reviewed
76	P03956	MMP1	Interstitial collagenase	reviewed
77	P08253	MMP2	72 kDa type IV collagenase	reviewed
78	P08254	MMP3	Stromelysin-1	reviewed
79	P14780	MMP9	Matrix metalloproteinase-9	reviewed
80	P05164	MPO	Myeloperoxidase	reviewed
81	P01106	MYC	Myc proto-oncogene protein	reviewed
82	Q15788	NCOA1	Nuclear receptor coactivator 1	reviewed
83	Q15596	NCOA2	Nuclear receptor coactivator 2	reviewed
84	Q16236	NFE2L2	Nuclear factor erythroid 2-related factor 2	reviewed
85	P25963	NFKBIA	NF-kappa-B inhibitor alpha	reviewed
86	P29475	NOS1	Nitric oxide synthase, brain	reviewed
87	P35228	NOS2	Nitric oxide synthase, inducible	reviewed
88	P29474	NOS3	Nitric oxide synthase, endothelial	reviewed
89	P04150	NR3C1	Glucocorticoid receptor	reviewed
90	P08235	NR3C2	Mineralocorticoid receptor	reviewed
91	P35372	OPRM1	Mu-type opioid receptor	reviewed
92	P12004	PCNA	Proliferating cell nuclear antigen	reviewed
93	P16284	PECAM1	Platelet endothelial cell adhesion molecule	reviewed
94	P06401	PGR	Progesterone receptor	reviewed
95	P48736	PIK3CG	dylinositol 4,5-bisphosphate 3-kinase catalytic subunit gamm	reviewed
96	P00750	PLAT	Tissue-type plasminogen activator	reviewed
97	P00749	PLAU	Urokinase-type plasminogen activator	reviewed
98	P27169	PON1	Serum paraoxonase/arylesterase 1	reviewed
99	Q07869	PPARA	Peroxisome proliferator-activated receptor alpha	reviewed
100	P37231	PPARG	Peroxisome proliferator-activated receptor gamma	reviewed
101	P05771	PRKCB	Protein kinase C beta type	reviewed
102	P60484	PTEN	3,4,5-trisphosphate 3-phosphatase and dual-specificity protei	reviewed
103	P43115	PTGER3	Prostaglandin E2 receptor EP3 subtype	reviewed
104	O14684	PTGES	Prostaglandin E synthase	reviewed
105	P23219	PTGS1	Prostaglandin G/H synthase 1	reviewed
106	P35354	PTGS2	Prostaglandin G/H synthase 2	reviewed

107	P04049	RAF1	RAF proto-oncogene serine/threonine-protein kinase	reviewed
108	P06400	RB1	Retinoblastoma-associated protein	reviewed
109	Q04206	RELA	Transcription factor p65	reviewed
110	P19793	RXRA	Retinoic acid receptor RXR-alpha	reviewed
111	P16581	SELE	E-selectin	reviewed
112	P05121	SERPINE1	Plasminogen activator inhibitor 1	reviewed
113	P14672	SLC2A4	olute carrier family 2, facilitated glucose transporter member	reviewed
114	P31645	SLC6A4	Sodium-dependent serotonin transporter	reviewed
115	P03973	SLPI	Antileukoproteinase	reviewed
116	P00441	SOD1	Superoxide dismutase [Cu-Zn]	reviewed
117	P04179	SOD2	Superoxide dismutase [Mn], mitochondrial	reviewed
118	Q9BX95	SPP1	Sphingosine-1-phosphate phosphatase 1	reviewed
119	P01137	TGFB1	growth factor beta-1 proprotein [Cleaved into: Latency-assoc	reviewed
120	P01375	TNF	Tumor necrosis factor	reviewed
121	P20333	TNFRSF1B	Tumor necrosis factor receptor superfamily member 1B	reviewed
122	P11388	TOP2A	DNA topoisomerase 2-alpha	reviewed
123	Q02880	TOP2B	DNA topoisomerase 2-beta	reviewed
124	P04637	TP53	Cellular tumor antigen p53	reviewed
125	Q8NER1	TRPV1	ansient receptor potential cation channel subfamily V membe	reviewed
126	P19320	VCAM1	Vascular cell adhesion protein 1	reviewed
127	P15692	VEGFA	Vascular endothelial growth factor A	reviewed
128	P47989	XDH	thine dehydrogenase/oxidase [Includes: Xanthine dehydroger	reviewed

Supplement table 5. KEGG Analysis

Term_ID	Term_description	ListHit	ListTotal	PopHit	PopTotal	GeneRatio	BgRatio	Enrichment_scor	GeneIds	GeneSymbol	p-value	FDR_bh	Classification_level1
path:hsa04657	IL-17 signaling pathway	23	124	93	7246	0.185483871	0.012834667	14.45178633	627;4318;43	XCL10;MM	3.95E-21	1.49E-19	Organismal Systems
path:hsa04668	TNF signaling pathway	23	124	110	7246	0.185483871	0.015180789	12.21832845	3627;4318;4	CL10;MMI	2.45E-19	6.15E-18	Environmental Information Processing
path:hsa01522	Endocrine resistance	21	124	96	7246	0.169354839	0.013248689	12.7827621	3725;5894;2	;JUN;RAF1	3.78E-18	6.58E-17	Human Diseases
path:hsa04151	PI3K-Akt signaling pathway	32	124	353	7246	0.258064516	0.048716533	5.29726766	064;842;59	B2;CASP9;	1.99E-15	2.37E-14	Environmental Information Processing
path:hsa04210	Apoptosis	21	124	135	7246	0.169354839	0.018630969	9.089964158	;5894;4170;	;MCL1;CA	5.84E-15	6.00E-14	Cellular Processes
path:hsa04218	Cellular senescence	21	124	158	7246	0.169354839	0.021805134	7.766741527	5970;890;35	12;RELA;C	1.48E-13	1.24E-12	Cellular Processes
path:hsa04926	Relaxin signaling pathway	19	124	130	7246	0.153225806	0.017940933	8.54057072	3725;5894;4	UN;RAF1;	4.15E-13	2.93E-12	Organismal Systems
path:hsa04068	FoxO signaling pathway	19	124	132	7246	0.153225806	0.018216947	8.411168133	586;595;102	1;IL10;CCN	5.51E-13	3.78E-12	Environmental Information Processing
path:hsa04066	HIF-1 signaling pathway	17	124	100	7246	0.137096774	0.013800718	9.934032258	1956;3458;1	R;IFNG;EGI	6.16E-13	3.97E-12	Environmental Information Processing
path:hsa04115	p53 signaling pathway	15	124	72	7246	0.120967742	0.009936517	12.17405914	157;842;102	SP9;CDKN	6.81E-13	4.27E-12	Cellular Processes
path:hsa04620	Toll-like receptor signaling pathway	17	124	102	7246	0.137096774	0.014076732	9.739247312	725;5599;36	APK8;CXCI	8.63E-13	5.27E-12	Organismal Systems
path:hsa04625	C-type lectin receptor signaling pathway	17	124	104	7246	0.137096774	0.014352746	9.551954094	124;3725;58	NF;JUN;RA	1.20E-12	7.15E-12	Organismal Systems
path:hsa04659	Th17 cell differentiation	17	124	105	7246	0.137096774	0.014490754	9.460983103	5599;3458;1	PK8;IFNG;	1.41E-12	8.19E-12	Organismal Systems
path:hsa04010	MAPK signaling pathway	26	124	295	7246	0.209677419	0.040712117	5.150246036	40;7124;372	GFB1;TNF	2.61E-12	1.47E-11	Environmental Information Processing
path:hsa04660	T cell receptor signaling pathway	16	124	101	7246	0.129032258	0.013938725	9.257106356	4;3565;3586	F1;IL4;IL10	9.57E-12	4.92E-11	Organismal Systems
path:hsa04915	Estrogen signaling pathway	17	124	136	7246	0.137096774	0.018768976	7.304435484	318;1956;20	;EGFR;ESF	1.02E-10	4.73E-10	Organismal Systems
path:hsa04064	NF-kappa B signaling pathway	14	124	93	7246	0.112903226	0.012834667	8.796739507	;3383;3576;	AM1;CXCI	4.17E-10	1.75E-09	Environmental Information Processing
path:hsa04919	Thyroid hormone signaling pathway	15	124	116	7246	0.120967742	0.016008832	7.55631257	609;2932;10	;GSK3B;NC	8.64E-10	3.42E-09	Organismal Systems
path:hsa04012	ErbB signaling pathway	13	124	85	7246	0.10483871	0.01173061	8.937191651	956;4609;1	;MYC;EGF	1.49E-09	5.82E-09	Environmental Information Processing
path:hsa04917	Prolactin signaling pathway	11	124	70	7246	0.088709677	0.009660502	9.182718894	2932;2099;5	SK3B;ESR1	2.17E-08	7.56E-08	Organismal Systems
path:hsa04370	VEGF signaling pathway	10	124	59	7246	0.080645161	0.008142423	9.9043193	4;842;5743;	SP9;PTGS2	4.63E-08	1.56E-07	Environmental Information Processing
path:hsa04215	Apoptosis - multiple species	8	124	33	7246	0.064516129	0.004554237	14.16617791	1;5599;842;	APK8;CAS	5.69E-08	1.89E-07	Cellular Processes
path:hsa04630	Jak-STAT signaling pathway	15	124	162	7246	0.120967742	0.022357163	5.410692951	1950;3458;3	GF;IFNG;IL	8.83E-08	2.85E-07	Environmental Information Processing
path:hsa04722	Neurotrophin signaling pathway	13	124	119	7246	0.10483871	0.016422854	6.383708322	5894;5599;2	APK8;GSK	9.67E-08	3.08E-07	Organismal Systems
path:hsa04621	NOD-like receptor signaling pathway	15	124	167	7246	0.120967742	0.023047198	5.248696156	70;7124;37	LA;TNF;JU	1.32E-07	4.10E-07	Organismal Systems
path:hsa04920	Adipocytokine signaling pathway	10	124	69	7246	0.080645161	0.009522495	8.468910706	4;5599;207;	K8;AKT1;T	2.17E-07	6.45E-07	Organismal Systems
path:hsa04931	Insulin resistance	12	124	107	7246	0.096774194	0.014766768	6.55351221	6;5599;2932	PK8;GSK3E	2.31E-07	6.79E-07	Human Diseases
path:hsa04662	B cell receptor signaling pathway	10	124	70	7246	0.080645161	0.009660502	8.347926267	4;2932;557	K3B;PRKCE	2.49E-07	7.22E-07	Organismal Systems
path:hsa04658	Th1 and Th2 cell differentiation	11	124	90	7246	0.088709677	0.012420646	7.142114695	599;4792;23	APK8;NFK	3.13E-07	8.84E-07	Organismal Systems
path:hsa01523	Antifolate resistance	7	124	31	7246	0.056451613	0.004278222	13.19510926	69;5970;102	;RELA;ABC	6.78E-07	1.87E-06	Human Diseases
path:hsa04110	Cell cycle	12	124	124	7246	0.096774194	0.01711289	5.655046826	;4609;2932;	YC;GSK3B;	1.16E-06	3.17E-06	Cellular Processes
path:hsa05020	Prion diseases	7	124	34	7246	0.056451613	0.004692244	12.03083491	569;581;20	;BAX;ELK	1.33E-06	3.58E-06	Human Diseases
path:hsa05330	Allograft rejection	7	124	35	7246	0.056451613	0.004830251	11.68709677	586;3458;35	IL10;IFNG;	1.64E-06	4.36E-06	Human Diseases
path:hsa04014	Ras signaling pathway	16	124	232	7246	0.129032258	0.032017665	4.03003337	3;2002;1956	ELK1;EGFF	1.82E-06	4.78E-06	Environmental Information Processing
path:hsa05211	Renal cell carcinoma	9	124	69	7246	0.072580645	0.009522495	7.622019635	026;7040;3	;TGFB1;HII	2.25E-06	5.83E-06	Human Diseases
path:hsa05332	Graft-versus-host disease	7	124	37	7246	0.056451613	0.005106266	11.05536181	552;3458;3	A;IFNG;FA	2.44E-06	6.26E-06	Human Diseases
path:hsa04622	RIG-I-like receptor signaling pathway	9	124	70	7246	0.072580645	0.009660502	7.513133641	70;5599;36	A;MAPK8;	2.54E-06	6.45E-06	Organismal Systems
path:hsa05231	Choline metabolism in cancer	10	124	99	7246	0.080645161	0.01366271	5.902574128	6;1950;557	EGF;PRKCE	6.44E-06	1.62E-05	Human Diseases
path:hsa05203	Viral carcinogenesis	14	124	200	7246	0.112903226	0.027601435	4.090483871	5;890;1026;	;CDKN1A;	7.11E-06	1.77E-05	Human Diseases
path:hsa04921	Oxytocin signaling pathway	12	124	152	7246	0.096774194	0.020977091	4.613327674	6;5743;557	2;PRKCB;C	9.91E-06	2.44E-05	Organismal Systems
path:hsa05221	Acute myeloid leukemia	8	124	66	7246	0.064516129	0.009108474	7.083088954	4;4353;460	MPO;MYC;	1.47E-05	3.57E-05	Human Diseases
path:hsa04664	Fc epsilon RI signaling pathway	8	124	67	7246	0.064516129	0.009246481	6.977371208	;5894;5599	;RAF1;MAI	1.64E-05	3.95E-05	Organismal Systems
path:hsa04062	Chemokine signaling pathway	13	124	187	7246	0.10483871	0.025807342	4.062359841	347;2932;6	GSK3B;CX	1.66E-05	3.95E-05	Organismal Systems
path:hsa04371	Apelin signaling pathway	11	124	137	7246	0.088709677	0.018906983	4.691900165	595;5054;20	SERPINE1;	2.02E-05	4.76E-05	Environmental Information Processing
path:hsa04912	GnRH signaling pathway	9	124	93	7246	0.072580645	0.012834667	5.655046826	599;2002;19	K8;ELK1;E	2.70E-05	6.29E-05	Organismal Systems
path:hsa04060	Cytokine-cytokine receptor interaction	16	124	292	7246	0.129032258	0.040298096	3.201944322	7;3552;3586	IL1A;IL10;	3.40E-05	7.83E-05	Environmental Information Processing
path:hsa05204	Chemical carcinogenesis	8	124	80	7246	0.064516129	0.011040574	5.843548387	;1545;5743;	YP1B1;PTG	6.06E-05	0.00013845	Human Diseases
path:hsa04940	Type I diabetes mellitus	6	124	41	7246	0.048387097	0.005658294	8.551534225	2;3458;356;	IFNG;FASI	6.13E-05	0.000138457	Human Diseases
path:hsa05131	Shigellosis	7	124	65	7246	0.056451613	0.008970466	6.293052109	970;5599;35	;MAPK8;C	0.000110874	0.000248094	Human Diseases
path:hsa04672	Intestinal immune network for IgA production	6	124	46	7246	0.048387097	0.00634833	7.622019635	;3586;7040;	6;IL10;TGI	0.000119015	0.000263701	Organismal Systems
path:hsa04024	cAMP signaling pathway	12	124	199	7246	0.096774194	0.027463428	3.523747771	;1813;2353	RD2;FOS;A	0.000141907	0.000311368	Environmental Information Processing
path:hsa04913	Ovarian steroidogenesis	6	124	49	7246	0.048387097	0.006762352	7.155365372	;1545;5743;	P1B1;PTGS	0.000170394	0.000370279	Organismal Systems
path:hsa04914	Progesterone-mediated oocyte maturation	8	124	99	7246	0.064516129	0.01366271	4.722059303	1;5894;5599	AF1;MAPK	0.0002732	0.000588029	Organismal Systems
path:hsa04140	Autophagy - animal	9	124	128	7246	0.072580645	0.017664919	4.10874496	894;5599;20	1;MAPK8;	0.000323889	0.000690556	Cellular Processes

path:hsa04928	Parathyroid hormone synthesis, secretion and action	8	124	106	7246	0.064516129	0.014628761	4.410225198	6;5579;2353	KCB;FOS;	0.00043561	0.000911553	Organismal Systems
path:hsa00140	Steroid hormone biosynthesis	6	124	60	7246	0.048387097	0.008280431	5.843548387	;1545;1588;	P1B1;CYP1	0.000523343	0.001085096	Metabolism
path:hsa04623	Cytosolic DNA-sensing pathway	6	124	62	7246	0.048387097	0.008556445	5.655046826	;3569;5970;	6;RELA;CX	0.000624988	0.001283972	Organismal Systems
path:hsa04670	Leukocyte transendothelial migration	8	124	112	7246	0.064516129	0.015456804	4.173963134	;4318;5175;	MP9;PECA	0.000630623	0.001283972	Organismal Systems
path:hsa04726	Serotonergic synapse	8	124	113	7246	0.064516129	0.015594811	4.137025407	;5742;5743;	TGS1;PTGS	0.000669089	0.001350125	Organismal Systems
path:hsa04540	Gap junction	7	124	88	7246	0.056451613	0.012144632	4.648277126	956;1950;18	R;EGF;DRD	0.000730767	0.001461534	Cellular Processes
path:hsa04015	Rap1 signaling pathway	11	124	206	7246	0.088709677	0.028429478	3.120341372	956;1950;1	R;EGF;DRD	0.000763346	0.001513299	Environmental Information Processing
path:hsa04137	Mitophagy - animal	6	124	65	7246	0.048387097	0.008970466	5.394044665	;5970;3725;	RELA;JUN;	0.000805442	0.001582868	Cellular Processes
path:hsa05310	Asthma	4	124	28	7246	0.032258065	0.003864201	8.347926267	;7124;3586;	LG;TNF;IL	0.001217053	0.002371155	Human Diseases
path:hsa00980	Metabolism of xenobiotics by cytochrome P450	6	124	72	7246	0.048387097	0.009936517	4.869623656	;1545;1544;	P1B1;CYP1	0.00138238	0.002670238	Metabolism
path:hsa04650	Natural killer cell mediated cytotoxicity	8	124	127	7246	0.064516129	0.017526911	3.680975362	3;3458;557	G;PRKCB;	0.001436461	0.002751187	Organismal Systems
path:hsa05320	Autoimmune thyroid disease	5	124	50	7246	0.040322581	0.006900359	5.843548387	586;356;355;	IL10;FASL	0.001549262	0.002942296	Human Diseases
path:hsa04217	Necroptosis	9	124	162	7246	0.072580645	0.022357163	3.246415771	599;3552;34	PK8;IL1A;I	0.001764533	0.003323204	Cellular Processes
path:hsa00232	Caffeine metabolism	2	124	5	7246	0.016129032	0.000690036	23.37419355	1544;7498	YP1A2;XD	0.002808655	0.005245918	Metabolism
path:hsa04725	Cholinergic synapse	7	124	112	7246	0.056451613	0.015456804	3.652217742	2353;207;59	S;AKT1;B	0.00297603	0.005512974	Organismal Systems
path:hsa04211	Longevity regulating pathway	6	124	89	7246	0.048387097	0.012282639	3.939470823	;5970;6648;	ELA;SOD2;	0.004063158	0.007465639	Organismal Systems
path:hsa04150	mTOR signaling pathway	8	124	151	7246	0.064516129	0.020839084	3.095919675	4;5894;2932	F1;GSK3B;	0.004234255	0.007717272	Environmental Information Processing
path:hsa00590	Arachidonic acid metabolism	5	124	63	7246	0.040322581	0.008694452	4.637736815	44;5742;574	C3;PTGS1;	0.004308491	0.007789752	Metabolism
path:hsa04934	Cushing syndrome	8	124	154	7246	0.064516129	0.021253105	3.035609552	;595;1029;1	D1;CDKN2	0.00476786	0.008551875	Human Diseases
path:hsa04611	Platelet activation	7	124	123	7246	0.056451613	0.016974883	3.325596643	846;5742;20	S3;PTGS1;	0.005001203	0.008899778	Organismal Systems
path:hsa00220	Arginine biosynthesis	3	124	21	7246	0.024193548	0.002898151	8.347926267	46;4843;48	S3;NOS2;N	0.005193879	0.009170442	Metabolism
path:hsa05010	Alzheimer disease	8	124	164	7246	0.064516129	0.022633177	2.850511408	4;842;4842;	SP9;NOS1;	0.006926634	0.012135033	Human Diseases
path:hsa04728	Dopaminergic synapse	7	124	131	7246	0.056451613	0.01807894	3.122506772	813;2932;5	RD2;GSK3	0.007026516	0.012215328	Organismal Systems
path:hsa04930	Type II diabetes mellitus	4	124	46	7246	0.032258065	0.00634833	5.081346424	;7124;5599;	NF;MAPK	0.007657262	0.013210238	Human Diseases
path:hsa04910	Insulin signaling pathway	7	124	137	7246	0.056451613	0.018906983	2.98575465	599;2002;2	K8;ELK1;G	0.008905336	0.015247014	Organismal Systems
path:hsa00983	Drug metabolism - other enzymes	5	124	77	7246	0.040322581	0.010626553	3.79451194	950;4353;74	TP1;MPO;	0.010025276	0.017035431	Metabolism
path:hsa04610	Complement and coagulation cascades	5	124	79	7246	0.040322581	0.010902567	3.698448346	147;5054;21	;SERPINE1	0.011132006	0.018774875	Organismal Systems
path:hsa04072	Phospholipase D signaling pathway	7	124	145	7246	0.056451613	0.020011041	2.821023359	576;1950;2	8;EGF;AK	0.011954515	0.020012744	Environmental Information Processing
path:hsa04310	Wnt signaling pathway	7	124	146	7246	0.056451613	0.020149048	2.801701281	599;4609;2	;MYC;GSK	0.012382865	0.020577407	Environmental Information Processing
path:hsa04020	Calcium signaling pathway	8	124	182	7246	0.064516129	0.025117306	2.568592698	;5733;2064;	TGER3;ER	0.012580662	0.020753501	Environmental Information Processing
path:hsa04923	Regulation of lipolysis in adipocytes	4	124	54	7246	0.032258065	0.007452388	4.328554361	2;5733;5743	TGER3;PT	0.013369021	0.021894194	Organismal Systems
path:hsa04350	TGF-beta signaling pathway	5	124	84	7246	0.040322581	0.011592603	3.478302611	040;3458;55	B1;IFNG;M	0.014257655	0.02318151	Environmental Information Processing
path:hsa05016	Huntington disease	8	124	187	7246	0.064516129	0.025807342	2.499913748	7;581;842;6	AX;CASP9	0.014626948	0.023612073	Human Diseases
path:hsa04730	Long-term depression	4	124	60	7246	0.032258065	0.008280431	3.895698925	;5579;4842;	KCB;NOS1	0.019067828	0.030562617	Organismal Systems
path:hsa04640	Hematopoietic cell lineage	5	124	94	7246	0.040322581	0.012972675	3.108270419	569;3552;35	IL6;IL1A;IL	0.02217148	0.035287003	Organismal Systems
path:hsa05217	Basal cell carcinoma	4	124	63	7246	0.032258065	0.008694452	3.710189452	7;2932;1026	K3B;CDKN	0.022403785	0.03540738	Human Diseases
path:hsa04960	Aldosterone-regulated sodium reabsorption	3	124	37	7246	0.024193548	0.005106266	4.738012206	06;5579;55	2;PRKCB;M	0.02489908	0.039077723	Organismal Systems
path:hsa04750	Inflammatory mediator regulation of TRP channels	5	124	99	7246	0.040322581	0.01366271	2.951287064	432;7442;55	4;TRPV1;M	0.027023216	0.042118944	Organismal Systems
path:hsa00982	Drug metabolism - cytochrome P450	4	124	68	7246	0.032258065	0.009384488	3.437381404	;2950;1576;	STP1;CYP3	0.028712421	0.044445255	Metabolism
path:hsa00380	Tryptophan metabolism	3	124	40	7246	0.024193548	0.005520287	4.38266129	545;1544;15	1;CYP1A2;	0.030517254	0.046917683	Metabolism
path:hsa04550	ignaling pathways regulating pluripotency of stem cell	6	124	139	7246	0.048387097	0.019182998	2.522394987	4;4609;2932	MYC;GSK3	0.031624376	0.048291276	Cellular Processes
path:hsa04976	Bile secretion	4	124	71	7246	0.032258065	0.00979851	3.292139936	7;9429;1576	BCG2;CYP	0.032956089	0.049987089	Organismal Systems
path:hsa04520	Adherens junction	4	124	72	7246	0.032258065	0.009936517	3.246415771	;2064;4233;	RBB2;MET	0.034447959	0.051771358	Cellular Processes
path:hsa04514	Cell adhesion molecules (CAMs)	6	124	142	7246	0.048387097	0.019597019	2.469104952	;1364;6401;	LDN4;SEL	0.034590597	0.051771358	Environmental Information Processing
path:hsa03320	PPAR signaling pathway	4	124	74	7246	0.032258065	0.010212531	3.158674804	;4312;5465;	MMP1;PPA	0.037548243	0.055828308	Organismal Systems
path:hsa04340	Hedgehog signaling pathway	3	124	47	7246	0.024193548	0.006486337	3.729924502	596;2932;59	;GSK3B;C	0.045982828	0.067922348	Environmental Information Processing
path:hsa04390	Hippo signaling pathway	6	124	154	7246	0.048387097	0.021253105	2.276707164	;2932;595;5	3B;CCND1;	0.048229738	0.070778707	Environmental Information Processing
path:hsa00330	Arginine and proline metabolism	3	124	49	7246	0.024193548	0.006762352	3.577682686	46;4843;48	S3;NOS2;N	0.050992015	0.073873047	Metabolism
path:hsa05030	Cocaine addiction	3	124	49	7246	0.024193548	0.006762352	3.577682686	970;3725;18	LA;JUN;DR	0.050992015	0.073873047	Human Diseases
path:hsa04146	Peroxisome	4	124	83	7246	0.032258065	0.011454596	2.816167897	;4843;7498;	NOS2;XDH	0.053427626	0.076908558	Cellular Processes
path:hsa04152	AMPK signaling pathway	5	124	120	7246	0.040322581	0.016560861	2.434811828	890;595;207	A2;CCND1;	0.054481933	0.077929853	Environmental Information Processing
path:hsa04666	Fc gamma R-mediated phagocytosis	4	124	90	7246	0.032258065	0.012420646	2.597132616	4;5579;207;	KCB;AKT1	0.067940525	0.096569551	Organismal Systems
path:hsa04530	Tight junction	6	124	170	7246	0.048387097	0.02346122	2.062428843	9;2064;1364	RBB2;CLDN	0.07098474	0.100265945	Cellular Processes
path:hsa00790	Folate biosynthesis	2	124	26	7246	0.016129032	0.003588187	4.495037221	231;8644	R1B1;AKR	0.072312615	0.101507149	Metabolism
path:hsa04713	Circadian entrainment	4	124	96	7246	0.032258065	0.013248689	2.434811828	;4842;2353;	NOS1;FOS;	0.081842754	0.114175694	Organismal Systems
path:hsa00591	Linoleic acid metabolism	2	124	29	7246	0.016129032	0.004002208	4.03003337	1544;1576	P1A2;CYP3	0.08742772	0.121218802	Metabolism

path:hsa04723	Retrograde endocannabinoid signaling	5	124	141	7246	0.040322581	0.019459012	2.072180279	599;5743;55	K8;PTGS2;I	0.093767771	0.128433432	Organismal Systems
path:hsa04916	Melanogenesis	4	124	101	7246	0.032258065	0.013938725	2.314276589	;2932;5579;	K3B;PRKCI	0.094418907	0.128546223	Organismal Systems
path:hsa04261	Adrenergic signaling in cardiomyocytes	5	124	144	7246	0.040322581	0.019873033	2.029009857	207;596;529	T1;BCL2;PII	0.100322992	0.135766444	Organismal Systems
path:hsa00830	Retinol metabolism	3	124	67	7246	0.024193548	0.009246481	2.616514203	544;1576;15	2;CYP3A4;(	0.106739814	0.142740817	Metabolism
path:hsa04720	Long-term potentiation	3	124	67	7246	0.024193548	0.009246481	2.616514203	394;5579;55	;PRKCB;M	0.106739814	0.142740817	Organismal Systems
path:hsa05031	Amphetamine addiction	3	124	68	7246	0.024193548	0.009384488	2.578036053	725;5579;23	N;PRKCB;F	0.110341078	0.146688728	Human Diseases
path:hsa04022	cGMP-PKG signaling pathway	5	124	163	7246	0.040322581	0.02249517	1.792499505	894;207;529	;AKT1;PIK	0.146865297	0.194102673	Environmental Information Processing
path:hsa04216	Ferroptosis	2	124	40	7246	0.016129032	0.005520287	2.921774194	3162;7157	IMOX1;TP5	0.149163565	0.195993987	Cellular Processes
path:hsa04114	Oocyte meiosis	4	124	124	7246	0.032258065	0.01711289	1.885015609	;5241;891;5	R;CCNB1;N	0.162589742	0.212400472	Cellular Processes
path:hsa04973	Carbohydrate digestion and absorption	2	124	44	7246	0.016129032	0.006072316	2.656158358	5579;207	RKCB;AKT	0.173350542	0.223869842	Organismal Systems
path:hsa02010	ABC transporters	2	124	44	7246	0.016129032	0.006072316	2.656158358	10257;9429	BCC4;ABC(	0.173350542	0.223869842	Environmental Information Processing
path:hsa05410	Hypertrophic cardiomyopathy (HCM)	3	124	85	7246	0.024193548	0.01173061	2.062428843	569;7040;71	6;TGFB1;TI	0.177840473	0.228363335	Human Diseases
path:hsa05322	Systemic lupus erythematosus	4	124	130	7246	0.032258065	0.017940933	1.798014888	;7124;3586;	G;TNF;IL1(	0.182742489	0.233332218	Human Diseases
path:hsa04961	ndocrine and other factor-regulated calcium reabsorptic	2	124	47	7246	0.016129032	0.006486337	2.486616335	2099;5579	SR1;PRKCI	0.191882255	0.243625784	Organismal Systems
path:hsa05150	Staphylococcus aureus infection	2	124	52	7246	0.016129032	0.007176373	2.24751861	3586;3383	IL10;ICAM1	0.223283342	0.281910811	Human Diseases
path:hsa00480	Glutathione metabolism	2	124	53	7246	0.016129032	0.00731438	2.205112599	2950;2944	STP1;GSTN	0.229617337	0.288297323	Metabolism
path:hsa04080	Neuroactive ligand-receptor interaction	7	124	305	7246	0.056451613	0.042092189	1.341142253	733;1813;21	GER3;DRD	0.266847967	0.333191384	Environmental Information Processing
path:hsa04810	Regulation of actin cytoskeleton	5	124	213	7246	0.040322581	0.029395529	1.371724973	956;1950;21	GFR;EGF;F2	0.300502062	0.373150912	Cellular Processes
path:hsa04141	Protein processing in endoplasmic reticulum	4	124	164	7246	0.032258065	0.022633177	1.425255704	;5599;4780;	APK8;NFE2	0.308578863	0.381086464	Genetic Information Processing
path:hsa03430	Mismatch repair	1	124	23	7246	0.008064516	0.003174165	2.540673212	5111	PCNA	0.32807756	0.402964829	Genetic Information Processing
path:hsa04612	Antigen processing and presentation	2	124	69	7246	0.016129032	0.009522495	1.693782141	7124;3458	TNF;IFNG	0.331263879	0.404679117	Organismal Systems
path:hsa04360	Axon guidance	4	124	175	7246	0.032258065	0.024151256	1.335668203	;2932;4233;	SK3B;MET	0.351504398	0.427096741	Organismal Systems
path:hsa00562	Inositol phosphate metabolism	2	124	74	7246	0.016129032	0.010212531	1.579337402	5294;5728	IK3CG;PTE	0.362487692	0.438086729	Metabolism
path:hsa05012	Parkinson disease	3	124	129	7246	0.024193548	0.017802926	1.358964741	1813;842;83	2;CASP9;C	0.380247645	0.457106212	Human Diseases
path:hsa04270	Vascular smooth muscle contraction	3	124	131	7246	0.024193548	0.01807894	1.338217188	394;5579;55	;PRKCB;M	0.389570925	0.465836132	Organismal Systems
path:hsa04512	ECM-receptor interaction	2	124	82	7246	0.016129032	0.011316588	1.425255704	6696;1277	PP1;COL1A	0.411254319	0.48917619	Environmental Information Processing
path:hsa00052	Galactose metabolism	1	124	31	7246	0.008064516	0.004278222	1.885015609	231	AKR1B1	0.415039948	0.491094388	Metabolism
path:hsa03410	Base excision repair	1	124	33	7246	0.008064516	0.004554237	1.770772239	5111	PCNA	0.434975271	0.509349281	Genetic Information Processing
path:hsa00051	Fructose and mannose metabolism	1	124	33	7246	0.008064516	0.004554237	1.770772239	231	AKR1B1	0.434975271	0.509349281	Metabolism
path:hsa00040	Pentose and glucuronate interconversions	1	124	34	7246	0.008064516	0.004692244	1.718690702	231	AKR1B1	0.444688714	0.51803943	Metabolism
path:hsa05414	Dilated cardiomyopathy (DCM)	2	124	89	7246	0.016129032	0.012282639	1.313156941	7040;7124	TGFB1;TNI	0.452405132	0.524168393	Human Diseases
path:hsa04974	Protein digestion and absorption	2	124	90	7246	0.016129032	0.012420646	1.298566308	1803;1277	PP4;COL1A	0.458152738	0.524168393	Organismal Systems
path:hsa04970	Salivary secretion	2	124	90	7246	0.016129032	0.012420646	1.298566308	5579;4842	RKCB;NOS	0.458152738	0.524168393	Organismal Systems
path:hsa05340	Primary immunodeficiency	1	124	36	7246	0.008064516	0.004968258	1.623207885	959	CD40LG	0.463621418	0.524168393	Human Diseases
path:hsa03030	DNA replication	1	124	36	7246	0.008064516	0.004968258	1.623207885	5111	PCNA	0.463621418	0.524168393	Genetic Information Processing
path:hsa05032	Morphine addiction	2	124	91	7246	0.016129032	0.012558653	1.284296349	4988;5579	PRM1;PRK(	0.463865834	0.524168393	Human Diseases
path:hsa05033	Nicotine addiction	1	124	40	7246	0.008064516	0.005520287	1.460887097	1139	CHRNA7	0.499586941	0.56172462	Human Diseases
path:hsa04070	Phosphatidylinositol signaling system	2	124	99	7246	0.016129032	0.01366271	1.180514826	5579;5728	RKCB;PTE	0.508272835	0.568661687	Environmental Information Processing
path:hsa00860	Porphyrin and chlorophyll metabolism	1	124	42	7246	0.008064516	0.005796301	1.391321045	3162	HMOX1	0.516662036	0.575200099	Metabolism
path:hsa04922	Glucagon signaling pathway	2	124	103	7246	0.016129032	0.014214739	1.13466959	5465;207	PARA;AKT	0.5295721	0.586682816	Organismal Systems
path:hsa03050	Proteasome	1	124	45	7246	0.008064516	0.006210323	1.298566308	3458	IFNG	0.54119684	0.596636516	Genetic Information Processing
path:hsa03420	Nucleotide excision repair	1	124	46	7246	0.008064516	0.00634833	1.270336606	5111	PCNA	0.549097352	0.602407775	Genetic Information Processing
path:hsa04979	Cholesterol metabolism	1	124	50	7246	0.008064516	0.006900359	1.168709677	1071	CETP	0.579372413	0.632551524	Organismal Systems
path:hsa04724	Glutamatergic synapse	2	124	114	7246	0.016129032	0.015732818	1.025183928	5579;5594	KCB;MAPI	0.58488014	0.634336131	Organismal Systems
path:hsa04978	Mineral absorption	1	124	51	7246	0.008064516	0.007038366	1.145793801	3162	HMOX1	0.586620582	0.634336131	Organismal Systems
path:hsa05034	Alcoholism	3	124	180	7246	0.024193548	0.024841292	0.973924731	313;5894;55	2;RAF1;MA	0.599536666	0.64521565	Human Diseases
path:hsa00561	Glycerolipid metabolism	1	124	61	7246	0.008064516	0.008418438	0.957958752	231	AKR1B1	0.652622597	0.699017568	Metabolism
path:hsa05412	rhythmogenic right ventricular cardiomyopathy (ARV	1	124	72	7246	0.008064516	0.009936517	0.811603943	2697	GJA1	0.713201448	0.760299657	Human Diseases
path:hsa05100	Bacterial invasion of epithelial cells	1	124	74	7246	0.008064516	0.010212531	0.789668701	4233	MET	0.723030862	0.761514856	Human Diseases
path:hsa04918	Thyroid hormone synthesis	1	124	74	7246	0.008064516	0.010212531	0.789668701	5579	PRKCB	0.723030862	0.761514856	Organismal Systems
path:hsa04145	Phagosome	2	124	148	7246	0.016129032	0.020425062	0.789668701	4353;4842	MPO;NOS1	0.725167022	0.761514856	Cellular Processes
path:hsa04971	Gastric acid secretion	1	124	75	7246	0.008064516	0.010350538	0.779139785	5579	PRKCB	0.727819509	0.761514856	Organismal Systems
path:hsa03008	Ribosome biogenesis in eukaryotes	1	124	81	7246	0.008064516	0.011178581	0.721425727	3692	EIF6	0.754874612	0.786182776	Genetic Information Processing
path:hsa04911	Insulin secretion	1	124	85	7246	0.008064516	0.01173061	0.687476281	5579	PRKCB	0.771411443	0.79972012	Organismal Systems
path:hsa04727	GABAergic synapse	1	124	88	7246	0.008064516	0.012144632	0.664039589	5579	PRKCB	0.783083339	0.8081134	Organismal Systems
path:hsa04925	Aldosterone synthesis and secretion	1	124	96	7246	0.008064516	0.013248689	0.608702957	5579	PRKCB	0.811397128	0.833526141	Organismal Systems

path:hsa04142	Lysosome	1	124	123	7246	0.008064516	0.016974883	0.475085235	1509	CTSD	0.882502699	0.898403649	Cellular Processes
path:hsa04714	Thermogenesis	2	124	216	7246	0.016129032	0.02980955	0.541069295	5468;1432	ARG;MAPK	0.889265227	0.901228436	Organismal Systems
path:hsa04120	Ubiquitin mediated proteolysis	1	124	136	7246	0.008064516	0.018768976	0.429672676	4193	MDM2	0.906503969	0.914597755	Genetic Information Processing
path:hsa04144	Endocytosis	2	124	244	7246	0.016129032	0.033673751	0.478979376	4193;1956	MDM2;EGF	0.925613818	0.929727657	Cellular Processes
path:hsa00230	Purine metabolism	1	124	174	7246	0.008064516	0.024013249	0.335836114	7498	XDH	0.952173398	0.952173398	Metabolism

Supplement table 6. GO Analysis

Term_ID	Term_description	ListHit	ListTotal	PopHit	PopTotal	GeneRatio	BgRatio	Enrichment_score	GeneIds	GeneSymbol	p-value	FDR_bh	Category
GO:0045944	regulation of transcription from RNA polymerase II p	9	25	588	13332	0.36	0.04410441	8.16244898	6;1813;104	;DRD2;N	6.44E-07	0.000232314	biological_process
GO:0035924	lar response to vascular endothelial growth factor stir	3	25	13	13332	0.12	0.000975098	123.0646154	32;7422;37	14;VEGF	1.65E-06	0.000251687	biological_process
GO:0043536	itive regulation of blood vessel endothelial cell migra	3	25	14	13332	0.12	0.001050105	114.2742857	91;7422;47	VEGFA;N	2.09E-06	0.000251687	biological_process
GO:0045766	positive regulation of angiogenesis	4	25	59	13332	0.16	0.004425443	36.15457627	;1139;4780	NA7;NFE2	4.08E-06	0.000368434	biological_process
GO:0032526	response to retinoic acid	3	25	20	13332	0.12	0.00150015	79.992	68;2697;95	G;GJA1;P	6.50E-06	0.00046945	biological_process
GO:0042752	regulation of circadian rhythm	3	25	29	13332	0.12	0.002175218	55.16689655	68;7153;55	;TOP2A;	2.06E-05	0.001240032	biological_process
GO:0032922	circadian regulation of gene expression	3	25	33	13332	0.12	0.002475248	48.48	6;1813;104	;DRD2;N	3.06E-05	0.001310261	biological_process
GO:0003158	endothelium development	2	25	5	13332	0.08	0.000375038	213.312	3791;2697	KDR;GJA1	3.36E-05	0.001310261	biological_process
GO:0009416	response to light stimulus	2	25	5	13332	0.08	0.000375038	213.312	1813;2002	RD2;ELK	3.36E-05	0.001310261	biological_process
GO:0048010	ular endothelial growth factor receptor signaling path	3	25	35	13332	0.12	0.002625263	45.70971429	32;7422;37	14;VEGF	3.66E-05	0.001310261	biological_process
GO:0048511	rhythmic process	3	25	36	13332	0.12	0.00270027	44.44	68;7153;55	;TOP2A;	3.99E-05	0.001310261	biological_process
GO:0006265	DNA topological change	2	25	6	13332	0.08	0.000450045	177.76	7155;7153	P2B;TOP	5.04E-05	0.001399746	biological_process
GO:0051247	positive regulation of protein metabolic process	2	25	6	13332	0.08	0.000450045	177.76	1139;5599	RNA7;MA	5.04E-05	0.001399746	biological_process
GO:0000712	resolution of meiotic recombination intermediates	2	25	10	13332	0.08	0.000750075	106.656	7155;7153	P2B;TOP	0.000150525	0.003440277	biological_process
GO:0002042	cell migration involved in sprouting angiogenesis	2	25	10	13332	0.08	0.000750075	106.656	3791;7422	DR;VEGF	0.000150525	0.003440277	biological_process
GO:0043066	negative regulation of apoptotic process	5	25	284	13332	0.2	0.02130213	9.388732394	31;5599;33	;MAPK8;	0.000158564	0.003440277	biological_process
GO:0001525	angiogenesis	4	25	150	13332	0.16	0.011251125	14.2208	;3791;5294	DR;PIK3	0.000162008	0.003440277	biological_process
GO:0003682	chromatin binding	5	25	266	12391	0.2	0.021467194	9.316541353	2;7153;10	;TOP2A;N	0.000164035	0.017475883	molecular_function
GO:2000352	egative regulation of endothelial cell apoptotic proce	2	25	11	13332	0.08	0.000825083	96.96	3791;4780	DR;NFE2	0.000183763	0.003529243	biological_process
GO:0001666	response to hypoxia	3	25	60	13332	0.12	0.00450045	26.664	13;1139;74	CHRNA7;	0.00018575	0.003529243	biological_process
GO:0042629	mast cell granule	2	25	13	14857	0.08	0.000875008	91.42769231	231;5294	R1B1;PIK	0.000209643	0.017190758	cellular_component
GO:0032496	response to lipopolysaccharide	3	25	67	13332	0.12	0.005025503	23.87820896	97;6590;95	1;SLPI;PT	0.000257791	0.004227722	biological_process
GO:2000379	ve regulation of reactive oxygen species metabolic pr	2	25	13	13332	0.08	0.000975098	82.04307692	1432;4780	PK14;NFE	0.000260011	0.004227722	biological_process
GO:1900273	positive regulation of long-term synaptic potentiatior	2	25	13	13332	0.08	0.000975098	82.04307692	1813;1139	D2;CHRN	0.000260011	0.004227722	biological_process
GO:0010468	regulation of gene expression	3	25	68	13332	0.12	0.00510051	23.52705882	6;1432;55	MAPK14;	0.000269356	0.004227722	biological_process
GO:0001934	positive regulation of protein phosphorylation	3	25	70	13332	0.12	0.005250525	22.85485714	91;1139;74	CHRNA7;	0.000293482	0.004414456	biological_process
GO:0001764	neuron migration	3	25	73	13332	0.12	0.005475548	21.91561644	55;2697;55	B;GJA1;M	0.000332221	0.004797278	biological_process
GO:0002092	positive regulation of receptor internalization	2	25	15	13332	0.08	0.001125113	71.104	1813;7422	RD2;VEGF	0.000349211	0.00484866	biological_process
GO:0043491	protein kinase B signaling	2	25	16	13332	0.08	0.00120012	66.66	3791;5728	DR;PTEN	0.00039864	0.00532996	biological_process
GO:0046326	positive regulation of glucose import	2	25	17	13332	0.08	0.001275128	62.73882353	1432;4780	PK14;NFE	0.000451273	0.005818193	biological_process
GO:0042826	histone deacetylase binding	3	25	77	12391	0.12	0.006214188	19.31064935	53;7155;55	;TOP2B;	0.000480934	0.017475883	molecular_function
GO:0051894	positive regulation of focal adhesion assembly	2	25	18	13332	0.08	0.001350135	59.25333333	3791;7422	DR;VEGF	0.000507098	0.006312502	biological_process
GO:0051898	negative regulation of protein kinase B signaling	2	25	19	13332	0.08	0.001425143	56.13473684	1813;5728	RD2;PTE	0.000566106	0.006592397	biological_process
GO:0035094	response to nicotine	2	25	19	13332	0.08	0.001425143	56.13473684	1813;1139	D2;CHRN	0.000566106	0.006592397	biological_process
GO:0046982	protein heterodimerization activity	5	25	357	12391	0.2	0.028811234	6.941736695	6;1813;71	;DRD2;T	0.000637377	0.017475883	molecular_function
GO:0019899	enzyme binding	4	25	203	12391	0.16	0.016382859	9.766305419	;7155;5599	OP2B;MA	0.000675386	0.017475883	molecular_function
GO:0070374	positive regulation of ERK1 and ERK2 cascade	3	25	95	13332	0.12	0.007125713	16.84042105	91;1139;18	CHRNA7;	0.00071951	0.008116969	biological_process
GO:0004879	nuclear receptor activity	2	25	21	12391	0.08	0.001694778	47.20380952	5468;196	PARG;AH	0.000801646	0.017475883	molecular_function
GO:0010595	positive regulation of endothelial cell migration	2	25	24	13332	0.08	0.00180018	44.44	3791;7422	DR;VEGF	0.000908481	0.009938229	biological_process
GO:0051879	Hsp90 protein binding	2	25	24	12391	0.08	0.00193689	41.30333333	196;3791	AHR;KDR	0.001049692	0.019069409	molecular_function
GO:0070301	cellular response to hydrogen peroxide	2	25	28	13332	0.08	0.00210021	38.09142857	5599;4780	PK8;NFE	0.001238521	0.013150175	biological_process
GO:0001938	positive regulation of endothelial cell proliferation	2	25	30	13332	0.08	0.002250225	35.552	3791;7422	DR;VEGF	0.001422012	0.014259623	biological_process
GO:0043406	positive regulation of MAP kinase activity	2	25	30	13332	0.08	0.002250225	35.552	7422;5294	GFA;PIK3	0.001422012	0.014259623	biological_process
GO:0016328	lateral plasma membrane	2	25	35	14857	0.08	0.002355792	33.95885714	1813;2697	RD2;GJA	0.001563339	0.05033177	cellular_component
GO:0043679	axon terminus	2	25	38	14857	0.08	0.002557717	31.27789474	1813;2002	RD2;ELK	0.001841406	0.05033177	cellular_component
GO:0009636	response to toxic substance	2	25	35	13332	0.08	0.002625263	30.47314286	196;1813	AHR;DRD	0.00193392	0.018372237	biological_process
GO:0032147	activation of protein kinase activity	2	25	35	13332	0.08	0.002625263	30.47314286	1813;7422	RD2;VEGF	0.00193392	0.018372237	biological_process
GO:0005080	protein kinase C binding	2	25	35	12391	0.08	0.002824631	28.32228571	7155;7153	P2B;TOP	0.002232384	0.02859172	molecular_function

GO:0003707	steroid hormone receptor activity	2	25	36	12391	0.08	0.002905335	27.53555556	5468;6256	ARG;RXF	0.002360784	0.02859172	molecular_function
GO:0008144	drug binding	2	25	36	12391	0.08	0.002905335	27.53555556	1813;7153	RD2;TOP2	0.002360784	0.02859172	molecular_function
GO:0044212	transcription regulatory region DNA binding	3	25	143	12391	0.12	0.011540634	10.39804196	468;196;47	G;AHR;NI	0.002873887	0.028820408	molecular_function
GO:0030374	dependent nuclear receptor transcription coactivator a	2	25	40	12391	0.08	0.003228149	24.782	5468;10499	ARG;NCO	0.002908481	0.028820408	molecular_function
GO:0006915	apoptotic process	4	25	336	13332	0.16	0.02520252	6.348571429	1432;2697;	APK14;GJ	0.003297613	0.029979837	biological_process
GO:0006874	cellular calcium ion homeostasis	2	25	46	13332	0.08	0.003450345	23.18608696	1813;1139	D2;CHRN	0.003321866	0.029979837	biological_process
GO:0005737	cytoplasm	12	25	3289	14857	0.48	0.221377129	2.168245667	);332;196;	IRC5;AHR	0.003789997	0.07769494	cellular_component
GO:0010628	positive regulation of gene expression	3	25	176	13332	0.12	0.01320132	9.09	97;5599;47	MAPK8;N	0.004201212	0.036833704	biological_process
GO:0071356	cellular response to tumor necrosis factor	2	25	53	13332	0.08	0.003975398	20.12377358	1432;4780	PK14;NFE	0.004387394	0.036833704	biological_process
GO:0032091	negative regulation of protein binding	2	25	53	13332	0.08	0.003975398	20.12377358	5599;6590	IAPK8;SLI	0.004387394	0.036833704	biological_process
GO:0016301	kinase activity	2	25	50	12391	0.08	0.004035187	19.8256	5294;5599	3CG;MAI	0.004511816	0.040982325	molecular_function
GO:0016925	protein sumoylation	2	25	54	13332	0.08	0.004050405	19.75111111	7155;7153	)P2B;TOP	0.004550923	0.037338255	biological_process
GO:0007059	chromosome segregation	2	25	55	13332	0.08	0.004125413	19.392	332;7153	RC5;TOP	0.004717249	0.037842818	biological_process
GO:0071456	cellular response to hypoxia	2	25	56	13332	0.08	0.00420042	19.04571429	7422;4780	GFA;NFE	0.004886361	0.038347313	biological_process
GO:0007409	axonogenesis	2	25	66	13332	0.08	0.004950495	16.16	7155;1813	OP2B;DRI	0.006728525	0.039071008	biological_process
GO:0030336	negative regulation of cell migration	2	25	66	13332	0.08	0.004950495	16.16	1813;5728	RD2;PTEI	0.006728525	0.039071008	biological_process
GO:0007204	sitive regulation of cytosolic calcium ion concentrati	2	25	68	13332	0.08	0.00510051	15.68470588	2697;5294	JA1;PIK3C	0.00712939	0.039071008	biological_process
GO:0003700	DNA binding transcription factor activity	5	25	642	12391	0.2	0.051811799	3.860124611	)02;196;54	1;AHR;PI	0.008228598	0.050505078	molecular_function
GO:0097441	basal dendrite	1	25	5	14857	0.04	0.000336542	118.856	5599	MAPK8	0.0083864	0.087382327	cellular_component
GO:1903169	regulation of calcium ion transmembrane transport	1	25	5	13332	0.04	0.000375038	106.656	5294	PIK3CG	0.009342237	0.039071008	biological_process
GO:0071803	positive regulation of podosome assembly	1	25	5	13332	0.04	0.000375038	106.656	5599	MAPK8	0.009342237	0.039071008	biological_process
GO:0015867	ATP transport	1	25	5	13332	0.04	0.000375038	106.656	2697	GJA1	0.009342237	0.039071008	biological_process
GO:0035815	positive regulation of renal sodium excretion	1	25	5	13332	0.04	0.000375038	106.656	1813	DRD2	0.009342237	0.039071008	biological_process
GO:0071467	cellular response to pH	1	25	5	13332	0.04	0.000375038	106.656	2697	GJA1	0.009342237	0.039071008	biological_process
GO:0048149	behavioral response to ethanol	1	25	5	13332	0.04	0.000375038	106.656	1813	DRD2	0.009342237	0.039071008	biological_process
GO:0035148	tube formation	1	25	5	13332	0.04	0.000375038	106.656	7422	VEGFA	0.009342237	0.039071008	biological_process
GO:0071732	cellular response to nitric oxide	1	25	5	13332	0.04	0.000375038	106.656	5599	MAPK8	0.009342237	0.039071008	biological_process
GO:0140059	dendrite arborization	1	25	5	13332	0.04	0.000375038	106.656	1139	CHRNA7	0.009342237	0.039071008	biological_process
GO:0051146	striated muscle cell differentiation	1	25	5	13332	0.04	0.000375038	106.656	1432	MAPK14	0.009342237	0.039071008	biological_process
GO:0002070	epithelial cell maturation	1	25	5	13332	0.04	0.000375038	106.656	2697	GJA1	0.009342237	0.039071008	biological_process
GO:0051823	regulation of synapse structural plasticity	1	25	5	13332	0.04	0.000375038	106.656	1813	DRD2	0.009342237	0.039071008	biological_process
GO:0071276	cellular response to cadmium ion	1	25	5	13332	0.04	0.000375038	106.656	5599	MAPK8	0.009342237	0.039071008	biological_process
GO:0060754	positive regulation of mast cell chemotaxis	1	25	5	13332	0.04	0.000375038	106.656	7422	VEGFA	0.009342237	0.039071008	biological_process
GO:0035162	embryonic hemopoiesis	1	25	5	13332	0.04	0.000375038	106.656	3791	KDR	0.009342237	0.039071008	biological_process
GO:0051770	ve regulation of nitric-oxide synthase biosynthetic pr	1	25	5	13332	0.04	0.000375038	106.656	3791	KDR	0.009342237	0.039071008	biological_process
GO:0030522	intracellular receptor signaling pathway	1	25	5	13332	0.04	0.000375038	106.656	10499	NCOA2	0.009342237	0.039071008	biological_process
GO:0021853	cerebral cortex GABAergic interneuron migration	1	25	5	13332	0.04	0.000375038	106.656	1813	DRD2	0.009342237	0.039071008	biological_process
GO:0002526	acute inflammatory response	1	25	5	13332	0.04	0.000375038	106.656	9536	PTGES	0.009342237	0.039071008	biological_process
GO:0010818	T cell chemotaxis	1	25	5	13332	0.04	0.000375038	106.656	5294	PIK3CG	0.009342237	0.039071008	biological_process
GO:0030529	intracellular ribonucleoprotein complex	2	25	88	14857	0.08	0.005923134	13.50636364	7155;7153	)P2B;TOP	0.009524666	0.087382327	cellular_component
GO:0005021	:ular endothelial growth factor-activated receptor acti	1	25	5	12391	0.04	0.000403519	99.128	3791	KDR	0.010048958	0.050505078	molecular_function
GO:0004032	alditol:NADP+ 1-oxidoreductase activity	1	25	5	12391	0.04	0.000403519	99.128	231	AKR1B1	0.010048958	0.050505078	molecular_function
GO:0043184	vascular endothelial growth factor receptor 2 binding	1	25	5	12391	0.04	0.000403519	99.128	7422	VEGFA	0.010048958	0.050505078	molecular_function
GO:0035005	1-phosphatidylinositol-4-phosphate 3-kinase activity	1	25	5	12391	0.04	0.000403519	99.128	5294	PIK3CG	0.010048958	0.050505078	molecular_function
GO:0048273	mitogen-activated protein kinase p38 binding	1	25	5	12391	0.04	0.000403519	99.128	1432	MAPK14	0.010048958	0.050505078	molecular_function
GO:0071253	connexin binding	1	25	5	12391	0.04	0.000403519	99.128	2697	GJA1	0.010048958	0.050505078	molecular_function
GO:0015643	toxic substance binding	1	25	5	12391	0.04	0.000403519	99.128	1139	CHRNA7	0.010048958	0.050505078	molecular_function
GO:0043220	Schmidt-Lanterman incisure	1	25	6	14857	0.04	0.00040385	99.04666667	231	AKR1B1	0.010055559	0.087382327	cellular_component
GO:0008285	negative regulation of cell proliferation	3	25	243	13332	0.12	0.018226823	6.583703704	13;9536;57	2;PTGES;I	0.010229884	0.039071008	biological_process
GO:0030165	PDZ domain binding	2	25	77	12391	0.08	0.006214188	12.87376623	2697;5728	JA1;PTEN	0.010424461	0.050505078	molecular_function

GO:0005125	cytokine activity	2	25	79	12391	0.08	0.006375595	12.5478481	7422;3565	VEGFA;IL	0.010949724	0.050505078	molecular_function
GO:0060044	negative regulation of cardiac muscle cell proliferatio	1	25	6	13332	0.04	0.000450045	88.88	2697	GJA1	0.011200603	0.039071008	biological_process
GO:1903206	ative regulation of hydrogen peroxide-induced cell de	1	25	6	13332	0.04	0.000450045	88.88	4780	NFE2L2	0.011200603	0.039071008	biological_process
GO:0035994	response to muscle stretch	1	25	6	13332	0.04	0.000450045	88.88	1432	MAPK14	0.011200603	0.039071008	biological_process
GO:0010831	positive regulation of myotube differentiation	1	25	6	13332	0.04	0.000450045	88.88	1432	MAPK14	0.011200603	0.039071008	biological_process
GO:0032495	response to muramyl dipeptide	1	25	6	13332	0.04	0.000450045	88.88	1432	MAPK14	0.011200603	0.039071008	biological_process
GO:0071223	cellular response to lipoteichoic acid	1	25	6	13332	0.04	0.000450045	88.88	1432	MAPK14	0.011200603	0.039071008	biological_process
GO:0042770	signal transduction in response to DNA damage	1	25	6	13332	0.04	0.000450045	88.88	1432	MAPK14	0.011200603	0.039071008	biological_process
GO:0060373	n of ventricular cardiac muscle cell membrane depol	1	25	6	13332	0.04	0.000450045	88.88	2697	GJA1	0.011200603	0.039071008	biological_process
GO:0042304	regulation of fatty acid biosynthetic process	1	25	6	13332	0.04	0.000450045	88.88	3692	EIF6	0.011200603	0.039071008	biological_process
GO:0009410	response to xenobiotic stimulus	1	25	6	13332	0.04	0.000450045	88.88	196	AHR	0.011200603	0.039071008	biological_process
GO:0051272	positive regulation of cellular component movement	1	25	6	13332	0.04	0.000450045	88.88	7422	VEGFA	0.011200603	0.039071008	biological_process
GO:0002031	G-protein coupled receptor internalization	1	25	6	13332	0.04	0.000450045	88.88	1813	DRD2	0.011200603	0.039071008	biological_process
GO:0060158	holipase C-activating dopamine receptor signaling pa	1	25	6	13332	0.04	0.000450045	88.88	1813	DRD2	0.011200603	0.039071008	biological_process
GO:0060124	positive regulation of growth hormone secretion	1	25	6	13332	0.04	0.000450045	88.88	1813	DRD2	0.011200603	0.039071008	biological_process
GO:1901653	cellular response to peptide	1	25	6	13332	0.04	0.000450045	88.88	231	AKR1B1	0.011200603	0.039071008	biological_process
GO:0055118	negative regulation of cardiac muscle contraction	1	25	6	13332	0.04	0.000450045	88.88	5294	PIK3CG	0.011200603	0.039071008	biological_process
GO:0095500	acetylcholine receptor signaling pathway	1	25	6	13332	0.04	0.000450045	88.88	1139	CHRNA7	0.011200603	0.039071008	biological_process
GO:0031223	auditory behavior	1	25	6	13332	0.04	0.000450045	88.88	1813	DRD2	0.011200603	0.039071008	biological_process
GO:0071374	cellular response to parathyroid hormone stimulus	1	25	6	13332	0.04	0.000450045	88.88	2697	GJA1	0.011200603	0.039071008	biological_process
GO:0001516	prostaglandin biosynthetic process	1	25	6	13332	0.04	0.000450045	88.88	9536	PTGES	0.011200603	0.039071008	biological_process
GO:0005654	nucleoplasm	9	25	2356	14857	0.36	0.158578448	2.270169779	92;4780;206;	NFE2L2;	0.01138367	0.087382327	cellular_component
GO:0006357	lation of transcription from RNA polymerase II prom	3	25	254	13332	0.12	0.019051905	6.298582677	548;196;14	;AHR;MA	0.011532131	0.039071008	biological_process
GO:0005634	nucleus	13	25	4252	14857	0.52	0.28619506	1.816942615	780;2002;3	L2;ELK1;	0.011548356	0.087382327	cellular_component
GO:0005916	fascia adherens	1	25	7	14857	0.04	0.000471158	84.89714286	2697	GJA1	0.011722019	0.087382327	cellular_component
GO:0033010	paranodal junction	1	25	7	14857	0.04	0.000471158	84.89714286	231	AKR1B1	0.011722019	0.087382327	cellular_component
GO:0008083	growth factor activity	2	25	82	12391	0.08	0.006617706	12.08878049	7422;3565	VEGFA;IL	0.011759236	0.050505078	molecular_function
GO:0043023	ribosomal large subunit binding	1	25	6	12391	0.04	0.000484222	82.60666667	3692	EIF6	0.012047083	0.050505078	molecular_function
GO:0017081	chloride channel regulator activity	1	25	6	12391	0.04	0.000484222	82.60666667	1139	CHRNA7	0.012047083	0.050505078	molecular_function
GO:0001162	intronic transcription regulatory region sequence-spe	1	25	6	12391	0.04	0.000484222	82.60666667	10499	NCOA2	0.012047083	0.050505078	molecular_function
GO:0010629	negative regulation of gene expression	2	25	93	13332	0.08	0.006975698	11.4683871	3791;2697	KDR;GJA1	0.013011614	0.039071008	biological_process
GO:0001963	synaptic transmission, dopaminergic	1	25	7	13332	0.04	0.000525053	76.18285714	1813	DRD2	0.013055622	0.039071008	biological_process
GO:2000810	regulation of bicellular tight junction assembly	1	25	7	13332	0.04	0.000525053	76.18285714	2697	GJA1	0.013055622	0.039071008	biological_process
GO:0006693	prostaglandin metabolic process	1	25	7	13332	0.04	0.000525053	76.18285714	9536	PTGES	0.013055622	0.039071008	biological_process
GO:0090050	egulation of cell migration involved in sprouting ang	1	25	7	13332	0.04	0.000525053	76.18285714	7422	VEGFA	0.013055622	0.039071008	biological_process
GO:0042417	dopamine metabolic process	1	25	7	13332	0.04	0.000525053	76.18285714	1813	DRD2	0.013055622	0.039071008	biological_process
GO:0010759	positive regulation of macrophage chemotaxis	1	25	7	13332	0.04	0.000525053	76.18285714	1432	MAPK14	0.013055622	0.039071008	biological_process
GO:0019395	fatty acid oxidation	1	25	7	13332	0.04	0.000525053	76.18285714	1432	MAPK14	0.013055622	0.039071008	biological_process
GO:0061045	negative regulation of wound healing	1	25	7	13332	0.04	0.000525053	76.18285714	2697	GJA1	0.013055622	0.039071008	biological_process
GO:0006700	C21-steroid hormone biosynthetic process	1	25	7	13332	0.04	0.000525053	76.18285714	231	AKR1B1	0.013055622	0.039071008	biological_process
GO:0044597	daunorubicin metabolic process	1	25	7	13332	0.04	0.000525053	76.18285714	231	AKR1B1	0.013055622	0.039071008	biological_process
GO:0043666	regulation of phosphoprotein phosphatase activity	1	25	7	13332	0.04	0.000525053	76.18285714	1813	DRD2	0.013055622	0.039071008	biological_process
GO:0051967	gative regulation of synaptic transmission, glutamater	1	25	7	13332	0.04	0.000525053	76.18285714	1813	DRD2	0.013055622	0.039071008	biological_process
GO:0035767	endothelial cell chemotaxis	1	25	7	13332	0.04	0.000525053	76.18285714	7422	VEGFA	0.013055622	0.039071008	biological_process
GO:0016264	gap junction assembly	1	25	7	13332	0.04	0.000525053	76.18285714	2697	GJA1	0.013055622	0.039071008	biological_process
GO:1903071	tion of ER-associated ubiquitin-dependent protein ca	1	25	7	13332	0.04	0.000525053	76.18285714	4780	NFE2L2	0.013055622	0.039071008	biological_process
GO:0046697	decidualization	1	25	7	13332	0.04	0.000525053	76.18285714	2697	GJA1	0.013055622	0.039071008	biological_process
GO:0002675	positive regulation of acute inflammatory response	1	25	7	13332	0.04	0.000525053	76.18285714	5294	PIK3CG	0.013055622	0.039071008	biological_process
GO:0007614	short-term memory	1	25	7	13332	0.04	0.000525053	76.18285714	1139	CHRNA7	0.013055622	0.039071008	biological_process
GO:0030278	regulation of ossification	1	25	7	13332	0.04	0.000525053	76.18285714	1432	MAPK14	0.013055622	0.039071008	biological_process

GO:1902004	positive regulation of amyloid-beta formation	1	25	7	13332	0.04	0.000525053	76.18285714	1139	CHRNA7	0.013055622	0.039071008	biological_process
GO:0030432	peristalsis	1	25	7	13332	0.04	0.000525053	76.18285714	1813	DRD2	0.013055622	0.039071008	biological_process
GO:0044598	doxorubicin metabolic process	1	25	7	13332	0.04	0.000525053	76.18285714	231	AKR1B1	0.013055622	0.039071008	biological_process
GO:0048263	determination of dorsal identity	1	25	7	13332	0.04	0.000525053	76.18285714	5599	MAPK8	0.013055622	0.039071008	biological_process
GO:2001214	positive regulation of vasculogenesis	1	25	7	13332	0.04	0.000525053	76.18285714	3791	KDR	0.013055622	0.039071008	biological_process
GO:0030225	macrophage differentiation	1	25	7	13332	0.04	0.000525053	76.18285714	7422	VEGFA	0.013055622	0.039071008	biological_process
GO:0005243	gap junction channel activity	1	25	7	12391	0.04	0.000564926	70.80571429	2697	GJA1	0.014041336	0.05460806	molecular_function
GO:0008094	DNA-dependent ATPase activity	1	25	7	12391	0.04	0.000564926	70.80571429	7153	TOP2A	0.014041336	0.05460806	molecular_function
GO:0008284	positive regulation of cell proliferation	3	25	279	13332	0.12	0.020927093	5.734193548	91;1139;74	CHRNA7;V	0.014836595	0.039071008	biological_process
GO:0086014	atrial cardiac muscle cell action potential	1	25	8	13332	0.04	0.00060006	66.66	2697	GJA1	0.0149073	0.039071008	biological_process
GO:0060371	ation of atrial cardiac muscle cell membrane depolari	1	25	8	13332	0.04	0.00060006	66.66	2697	GJA1	0.0149073	0.039071008	biological_process
GO:0035810	positive regulation of urine volume	1	25	8	13332	0.04	0.00060006	66.66	1813	DRD2	0.0149073	0.039071008	biological_process
GO:0045475	locomotor rhythm	1	25	8	13332	0.04	0.00060006	66.66	10499	NCOA2	0.0149073	0.039071008	biological_process
GO:0045745	gulation of G-protein coupled receptor protein signali	1	25	8	13332	0.04	0.00060006	66.66	1813	DRD2	0.0149073	0.039071008	biological_process
GO:0007178	orane receptor protein serine/threonine kinase signalir	1	25	8	13332	0.04	0.00060006	66.66	1432	MAPK14	0.0149073	0.039071008	biological_process
GO:0048148	behavioral response to cocaine	1	25	8	13332	0.04	0.00060006	66.66	1813	DRD2	0.0149073	0.039071008	biological_process
GO:0033628	regulation of cell adhesion mediated by integrin	1	25	8	13332	0.04	0.00060006	66.66	5294	PIK3CG	0.0149073	0.039071008	biological_process
GO:1901386	ative regulation of voltage-gated calcium channel acti	1	25	8	13332	0.04	0.00060006	66.66	1813	DRD2	0.0149073	0.039071008	biological_process
GO:0050872	white fat cell differentiation	1	25	8	13332	0.04	0.00060006	66.66	5468	PPARG	0.0149073	0.039071008	biological_process
GO:2000279	negative regulation of DNA biosynthetic process	1	25	8	13332	0.04	0.00060006	66.66	2697	GJA1	0.0149073	0.039071008	biological_process
GO:0021756	striatum development	1	25	8	13332	0.04	0.00060006	66.66	1813	DRD2	0.0149073	0.039071008	biological_process
GO:0051481	gative regulation of cytosolic calcium ion concentrati	1	25	8	13332	0.04	0.00060006	66.66	1813	DRD2	0.0149073	0.039071008	biological_process
GO:0030949	ion of vascular endothelial growth factor receptor sig	1	25	8	13332	0.04	0.00060006	66.66	7422	VEGFA	0.0149073	0.039071008	biological_process
GO:0010039	response to iron ion	1	25	8	13332	0.04	0.00060006	66.66	1813	DRD2	0.0149073	0.039071008	biological_process
GO:0060134	prepulse inhibition	1	25	8	13332	0.04	0.00060006	66.66	1813	DRD2	0.0149073	0.039071008	biological_process
GO:0046855	inositol phosphate dephosphorylation	1	25	8	13332	0.04	0.00060006	66.66	5728	PTEN	0.0149073	0.039071008	biological_process
GO:0071498	cellular response to fluid shear stress	1	25	8	13332	0.04	0.00060006	66.66	4780	NFE2L2	0.0149073	0.039071008	biological_process
GO:0046686	response to cadmium ion	1	25	8	13332	0.04	0.00060006	66.66	5599	MAPK8	0.0149073	0.039071008	biological_process
GO:0008360	regulation of cell shape	2	25	100	13332	0.08	0.00750075	10.6656	3791;7422	DR;VEGF	0.014935732	0.039071008	biological_process
GO:0018105	peptidyl-serine phosphorylation	2	25	103	13332	0.08	0.007725773	10.35495146	1432;5599	PK14;MA	0.015795837	0.039534555	biological_process
GO:0043295	glutathione binding	1	25	8	12391	0.04	0.00064563	61.955	9536	PTGES	0.016031724	0.05460806	molecular_function
GO:0001223	transcription coactivator binding	1	25	8	12391	0.04	0.00064563	61.955	196	AHR	0.016031724	0.05460806	molecular_function
GO:0005161	platelet-derived growth factor receptor binding	1	25	8	12391	0.04	0.00064563	61.955	7422	VEGFA	0.016031724	0.05460806	molecular_function
GO:1990782	protein tyrosine kinase binding	1	25	8	12391	0.04	0.00064563	61.955	2697	GJA1	0.016031724	0.05460806	molecular_function
GO:0007507	heart development	2	25	104	13332	0.08	0.00780078	10.25538462	2697;5728	GJA1;PTEN	0.016087204	0.039534555	biological_process
GO:0001894	tissue homeostasis	1	25	9	13332	0.04	0.000675068	59.25333333	231	AKR1B1	0.016755642	0.039534555	biological_process
GO:0030224	monocyte differentiation	1	25	9	13332	0.04	0.000675068	59.25333333	7422	VEGFA	0.016755642	0.039534555	biological_process
GO:0071310	cellular response to organic substance	1	25	9	13332	0.04	0.000675068	59.25333333	5599	MAPK8	0.016755642	0.039534555	biological_process
GO:0007625	grooming behavior	1	25	9	13332	0.04	0.000675068	59.25333333	1813	DRD2	0.016755642	0.039534555	biological_process
GO:0050918	positive chemotaxis	1	25	9	13332	0.04	0.000675068	59.25333333	7422	VEGFA	0.016755642	0.039534555	biological_process
GO:0050930	induction of positive chemotaxis	1	25	9	13332	0.04	0.000675068	59.25333333	7422	VEGFA	0.016755642	0.039534555	biological_process
GO:0043303	mast cell degranulation	1	25	9	13332	0.04	0.000675068	59.25333333	5294	PIK3CG	0.016755642	0.039534555	biological_process
GO:0042098	T cell proliferation	1	25	9	13332	0.04	0.000675068	59.25333333	5294	PIK3CG	0.016755642	0.039534555	biological_process
GO:0035195	gene silencing by miRNA	1	25	9	13332	0.04	0.000675068	59.25333333	3692	EIF6	0.016755642	0.039534555	biological_process
GO:0097755	positive regulation of blood vessel diameter	1	25	9	13332	0.04	0.000675068	59.25333333	2697	GJA1	0.016755642	0.039534555	biological_process
GO:0071480	cellular response to gamma radiation	1	25	9	13332	0.04	0.000675068	59.25333333	2002	ELK1	0.016755642	0.039534555	biological_process
GO:0007512	adult heart development	1	25	9	13332	0.04	0.000675068	59.25333333	2697	GJA1	0.016755642	0.039534555	biological_process
GO:0014059	regulation of dopamine secretion	1	25	9	13332	0.04	0.000675068	59.25333333	1813	DRD2	0.016755642	0.039534555	biological_process
GO:0045121	membrane raft	2	25	121	14857	0.08	0.008144309	9.822809917	3791;2697	KDR;GJA1	0.017463711	0.110542274	cellular_component
GO:0015075	ion transmembrane transporter activity	1	25	9	12391	0.04	0.000726334	55.07111111	2697	GJA1	0.018018254	0.058873495	molecular_function

GO:0048514	blood vessel morphogenesis	1	25	10	13332	0.04	0.000750075	53.328	2697	GJA1	0.018600655	0.041449609	biological_process
GO:0032228	regulation of synaptic transmission, GABAergic	1	25	10	13332	0.04	0.000750075	53.328	1813	DRD2	0.018600655	0.041449609	biological_process
GO:0051403	stress-activated MAPK cascade	1	25	10	13332	0.04	0.000750075	53.328	5599	MAPK8	0.018600655	0.041449609	biological_process
GO:0051895	negative regulation of focal adhesion assembly	1	25	10	13332	0.04	0.000750075	53.328	5728	PTEN	0.018600655	0.041449609	biological_process
GO:0048169	regulation of long-term neuronal synaptic plasticity	1	25	10	13332	0.04	0.000750075	53.328	1813	DRD2	0.018600655	0.041449609	biological_process
GO:0050709	negative regulation of protein secretion	1	25	10	13332	0.04	0.000750075	53.328	1813	DRD2	0.018600655	0.041449609	biological_process
GO:0032793	ositive regulation of CREB transcription factor activi	1	25	10	13332	0.04	0.000750075	53.328	7422	VEGFA	0.018600655	0.041449609	biological_process
GO:0045824	negative regulation of innate immune response	1	25	10	13332	0.04	0.000750075	53.328	1813	DRD2	0.018600655	0.041449609	biological_process
GO:0086064	unication by electrical coupling involved in cardiac c	1	25	10	13332	0.04	0.000750075	53.328	2697	GJA1	0.018600655	0.041449609	biological_process
GO:0035257	nuclear hormone receptor binding	1	25	10	12391	0.04	0.000807037	49.564	10499	NCOA2	0.020000934	0.058873495	molecular_function
GO:0042166	acetylcholine binding	1	25	10	12391	0.04	0.000807037	49.564	1139	CHRNA7	0.020000934	0.058873495	molecular_function
GO:0005921	gap junction	1	25	12	14857	0.04	0.0008077	49.52333333	2697	GJA1	0.020014008	0.110542274	cellular_component
GO:0051209	release of sequestered calcium ion into cytosol	1	25	11	13332	0.04	0.000825083	48.48	1813	DRD2	0.020442345	0.04340992	biological_process
GO:0060307	on of ventricular cardiac muscle cell membrane repol	1	25	11	13332	0.04	0.000825083	48.48	2697	GJA1	0.020442345	0.04340992	biological_process
GO:0042307	positive regulation of protein import into nucleus	1	25	11	13332	0.04	0.000825083	48.48	1432	MAPK14	0.020442345	0.04340992	biological_process
GO:0001659	temperature homeostasis	1	25	11	13332	0.04	0.000825083	48.48	1813	DRD2	0.020442345	0.04340992	biological_process
GO:0002052	positive regulation of neuroblast proliferation	1	25	11	13332	0.04	0.000825083	48.48	1813	DRD2	0.020442345	0.04340992	biological_process
GO:0001816	cytokine production	1	25	11	13332	0.04	0.000825083	48.48	5294	PIK3CG	0.020442345	0.04340992	biological_process
GO:1901216	positive regulation of neuron death	1	25	11	13332	0.04	0.000825083	48.48	2002	ELK1	0.020442345	0.04340992	biological_process
GO:0006801	superoxide metabolic process	1	25	11	13332	0.04	0.000825083	48.48	6648	SOD2	0.020442345	0.04340992	biological_process
GO:0044853	plasma membrane raft	1	25	13	14857	0.04	0.000875008	45.71384615	1139	CHRNA7	0.021664372	0.110542274	cellular_component
GO:0035326	enhancer binding	1	25	11	12391	0.04	0.000887741	45.05818182	196	AHR	0.021979771	0.058873495	molecular_function
GO:0015464	acetylcholine receptor activity	1	25	11	12391	0.04	0.000887741	45.05818182	1139	CHRNA7	0.021979771	0.058873495	molecular_function
GO:0043403	skeletal muscle tissue regeneration	1	25	12	13332	0.04	0.00090009	44.44	2697	GJA1	0.022280716	0.044194167	biological_process
GO:2001235	positive regulation of apoptotic signaling pathway	1	25	12	13332	0.04	0.00090009	44.44	5599	MAPK8	0.022280716	0.044194167	biological_process
GO:0035584	ium-mediated signaling using intracellular calcium so	1	25	12	13332	0.04	0.00090009	44.44	3791	KDR	0.022280716	0.044194167	biological_process
GO:1901741	positive regulation of myoblast fusion	1	25	12	13332	0.04	0.00090009	44.44	1432	MAPK14	0.022280716	0.044194167	biological_process
GO:0090141	positive regulation of mitochondrial fission	1	25	12	13332	0.04	0.00090009	44.44	3791	KDR	0.022280716	0.044194167	biological_process
GO:0060045	ositive regulation of cardiac muscle cell proliferatio	1	25	12	13332	0.04	0.00090009	44.44	1432	MAPK14	0.022280716	0.044194167	biological_process
GO:0043434	response to peptide hormone	1	25	12	13332	0.04	0.00090009	44.44	2697	GJA1	0.022280716	0.044194167	biological_process
GO:0097150	neuronal stem cell population maintenance	1	25	12	13332	0.04	0.00090009	44.44	5599	MAPK8	0.022280716	0.044194167	biological_process
GO:0043278	response to morphine	1	25	12	13332	0.04	0.00090009	44.44	1813	DRD2	0.022280716	0.044194167	biological_process
GO:0010906	regulation of glucose metabolic process	1	25	12	13332	0.04	0.00090009	44.44	10499	NCOA2	0.022280716	0.044194167	biological_process
GO:2000377	egulation of reactive oxygen species metabolic proces	1	25	12	13332	0.04	0.00090009	44.44	3692	EIF6	0.022280716	0.044194167	biological_process
GO:0035278	miRNA mediated inhibition of translation	1	25	12	13332	0.04	0.00090009	44.44	3692	EIF6	0.022280716	0.044194167	biological_process
GO:0034220	ion transmembrane transport	2	25	126	13332	0.08	0.009450945	8.464761905	1139;2697	IRNA7;GJ.	0.023067841	0.04487523	biological_process
GO:0043027	pe endopeptidase inhibitor activity involved in apopt	1	25	12	12391	0.04	0.000968445	41.30333333	332	BIRC5	0.023954772	0.058873495	molecular_function
GO:0048037	cofactor binding	1	25	12	12391	0.04	0.000968445	41.30333333	332	BIRC5	0.023954772	0.058873495	molecular_function
GO:0008301	DNA binding, bending	1	25	12	12391	0.04	0.000968445	41.30333333	7153	TOP2A	0.023954772	0.058873495	molecular_function
GO:0042056	chemoattractant activity	1	25	12	12391	0.04	0.000968445	41.30333333	7422	VEGFA	0.023954772	0.058873495	molecular_function
GO:0001975	response to amphetamine	1	25	13	13332	0.04	0.000975098	41.02153846	1813	DRD2	0.024115774	0.04487523	biological_process
GO:1901214	regulation of neuron death	1	25	13	13332	0.04	0.000975098	41.02153846	1139	CHRNA7	0.024115774	0.04487523	biological_process
GO:0001502	cartilage condensation	1	25	13	13332	0.04	0.000975098	41.02153846	1432	MAPK14	0.024115774	0.04487523	biological_process
GO:0031954	positive regulation of protein autophosphorylation	1	25	13	13332	0.04	0.000975098	41.02153846	7422	VEGFA	0.024115774	0.04487523	biological_process
GO:0070371	ERK1 and ERK2 cascade	1	25	13	13332	0.04	0.000975098	41.02153846	3791	KDR	0.024115774	0.04487523	biological_process
GO:0045648	positive regulation of erythrocyte differentiation	1	25	13	13332	0.04	0.000975098	41.02153846	1432	MAPK14	0.024115774	0.04487523	biological_process
GO:0000470	maturation of LSU-rRNA	1	25	13	13332	0.04	0.000975098	41.02153846	3692	EIF6	0.024115774	0.04487523	biological_process
GO:0045995	regulation of embryonic development	1	25	13	13332	0.04	0.000975098	41.02153846	4780	NFE2L2	0.024115774	0.04487523	biological_process
GO:0000460	maturation of 5.8S rRNA	1	25	13	13332	0.04	0.000975098	41.02153846	3692	EIF6	0.024115774	0.04487523	biological_process
GO:0000077	DNA damage checkpoint	1	25	13	13332	0.04	0.000975098	41.02153846	1432	MAPK14	0.024115774	0.04487523	biological_process

GO:0031663	lipopolysaccharide-mediated signaling pathway	1	25	13	13332	0.04	0.000975098	41.02153846	1432	MAPK14	0.024115774	0.04487523	biological_process
GO:0005524	ATP binding	6	25	1153	12391	0.24	0.093051408	2.579219428	;7155;5599	P2B;MAPI	0.024271031	0.058873495	molecular_function
GO:0042803	protein homodimerization activity	4	25	559	12391	0.16	0.045113389	3.546618962	1813;1139;	D2;CHRN	0.024391969	0.058873495	molecular_function
GO:0045893	positive regulation of transcription, DNA-templated	3	25	339	13332	0.12	0.025427543	4.719292035	468;196;20	RG;AHR;E	0.024772012	0.045471151	biological_process
GO:0004672	protein kinase activity	2	25	122	12391	0.08	0.009845856	8.125245902	5294;5599	3CG;MAI	0.024884109	0.058873495	molecular_function
GO:0030660	Golgi-associated vesicle membrane	1	25	15	14857	0.04	0.001009625	39.61866667	2697	GJA1	0.024957099	0.110542274	cellular_component
GO:0046983	protein dimerization activity	2	25	123	12391	0.08	0.00992656	8.059186992	196;10499	HR;NCOA	0.025264481	0.058873495	molecular_function
GO:0004438	phosphatidylinositol-3-phosphatase activity	1	25	13	12391	0.04	0.001049149	38.12615385	5728	PTEN	0.025925943	0.058873495	molecular_function
GO:0004714	ansmembrane receptor protein tyrosine kinase activit	1	25	13	12391	0.04	0.001049149	38.12615385	3791	KDR	0.025925943	0.058873495	molecular_function
GO:0005216	ion channel activity	1	25	13	12391	0.04	0.001049149	38.12615385	1139	CHRNA7	0.025925943	0.058873495	molecular_function
GO:0043473	pigmentation	1	25	14	13332	0.04	0.001050105	38.09142857	1813	DRD2	0.025947526	0.045471151	biological_process
GO:0008306	associative learning	1	25	14	13332	0.04	0.001050105	38.09142857	1813	DRD2	0.025947526	0.045471151	biological_process
GO:0098586	cellular response to virus	1	25	14	13332	0.04	0.001050105	38.09142857	1432	MAPK14	0.025947526	0.045471151	biological_process
GO:0030316	osteoclast differentiation	1	25	14	13332	0.04	0.001050105	38.09142857	1432	MAPK14	0.025947526	0.045471151	biological_process
GO:0034614	cellular response to reactive oxygen species	1	25	14	13332	0.04	0.001050105	38.09142857	5599	MAPK8	0.025947526	0.045471151	biological_process
GO:0045907	positive regulation of vasoconstriction	1	25	14	13332	0.04	0.001050105	38.09142857	2697	GJA1	0.025947526	0.045471151	biological_process
GO:0010667	;ative regulation of cardiac muscle cell apoptotic proc	1	25	14	13332	0.04	0.001050105	38.09142857	4780	NFE2L2	0.025947526	0.045471151	biological_process
GO:0016310	phosphorylation	1	25	14	13332	0.04	0.001050105	38.09142857	5294	PIK3CG	0.025947526	0.045471151	biological_process
GO:0051924	regulation of calcium ion transport	1	25	14	13332	0.04	0.001050105	38.09142857	2697	GJA1	0.025947526	0.045471151	biological_process
GO:0030500	regulation of bone mineralization	1	25	14	13332	0.04	0.001050105	38.09142857	2697	GJA1	0.025947526	0.045471151	biological_process
GO:0010975	regulation of neuron projection development	1	25	14	13332	0.04	0.001050105	38.09142857	5728	PTEN	0.025947526	0.045471151	biological_process
GO:0000793	condensed chromosome	1	25	16	14857	0.04	0.001076933	37.1425	7153	TOP2A	0.02659947	0.110542274	cellular_component
GO:0005892	acetylcholine-gated channel complex	1	25	16	14857	0.04	0.001076933	37.1425	1139	CHRNA7	0.02659947	0.110542274	cellular_component
GO:0008022	protein C-terminus binding	2	25	129	12391	0.08	0.010410782	7.684341085	7155;7153	)P2B;TOP:	0.027596727	0.059615076	molecular_function
GO:0015721	bile acid and bile salt transport	1	25	15	13332	0.04	0.001125113	35.552	10499	NCOA2	0.027775978	0.047075718	biological_process
GO:0045931	positive regulation of mitotic cell cycle	1	25	15	13332	0.04	0.001125113	35.552	332	BIRC5	0.027775978	0.047075718	biological_process
GO:0031334	positive regulation of protein complex assembly	1	25	15	13332	0.04	0.001125113	35.552	7422	VEGFA	0.027775978	0.047075718	biological_process
GO:0050482	arachidonic acid secretion	1	25	15	13332	0.04	0.001125113	35.552	1813	DRD2	0.027775978	0.047075718	biological_process
GO:0060135	maternal process involved in female pregnancy	1	25	15	13332	0.04	0.001125113	35.552	231	AKR1B1	0.027775978	0.047075718	biological_process
GO:0001937	negative regulation of endothelial cell proliferation	1	25	15	13332	0.04	0.001125113	35.552	2697	GJA1	0.027775978	0.047075718	biological_process
GO:0002028	regulation of sodium ion transport	1	25	15	13332	0.04	0.001125113	35.552	1813	DRD2	0.027775978	0.047075718	biological_process
GO:0043234	protein complex	3	25	395	14857	0.12	0.026586794	4.513518987	6;7153;104	TOP2A;N(	0.027810367	0.110542274	cellular_component
GO:0097718	disordered domain specific binding	1	25	14	12391	0.04	0.001129852	35.40285714	2697	GJA1	0.027893292	0.059615076	molecular_function
GO:0004033	aldo-keto reductase (NADP) activity	1	25	14	12391	0.04	0.001129852	35.40285714	231	AKR1B1	0.027893292	0.059615076	molecular_function
GO:0005922	connexin complex	1	25	17	14857	0.04	0.001144242	34.95764706	2697	GJA1	0.028239185	0.110542274	cellular_component
GO:0005771	multivesicular body	1	25	17	14857	0.04	0.001144242	34.95764706	2697	GJA1	0.028239185	0.110542274	cellular_component
GO:0045663	positive regulation of myoblast differentiation	1	25	16	13332	0.04	0.00120012	33.33	1432	MAPK14	0.029601133	0.049472265	biological_process
GO:0014065	phosphatidylinositol 3-kinase signaling	1	25	16	13332	0.04	0.00120012	33.33	5294	PIK3CG	0.029601133	0.049472265	biological_process
GO:0042220	response to cocaine	1	25	16	13332	0.04	0.00120012	33.33	1813	DRD2	0.029601133	0.049472265	biological_process
GO:0001968	fibronectin binding	1	25	15	12391	0.04	0.001210556	33.04266667	7422	VEGFA	0.029856827	0.060266558	molecular_function
GO:0022848	acetylcholine-gated cation-selective channel activity	1	25	15	12391	0.04	0.001210556	33.04266667	1139	CHRNA7	0.029856827	0.060266558	molecular_function
GO:0016922	ligand-dependent nuclear receptor binding	1	25	15	12391	0.04	0.001210556	33.04266667	10499	NCOA2	0.029856827	0.060266558	molecular_function
GO:0097730	non-motile cilium	1	25	18	14857	0.04	0.00121155	33.01555556	1813	DRD2	0.029876248	0.110542274	cellular_component
GO:0016239	positive regulation of macroautophagy	1	25	17	13332	0.04	0.001275128	31.36941176	3791	KDR	0.031423	0.052035334	biological_process
GO:0042113	B cell activation	1	25	17	13332	0.04	0.001275128	31.36941176	3565	IL4	0.031423	0.052035334	biological_process
GO:0019838	growth factor binding	1	25	16	12391	0.04	0.00129126	30.9775	3791	KDR	0.031816554	0.063054625	molecular_function
GO:0098794	postsynapse	1	25	20	14857	0.04	0.001346167	29.714	1139	CHRNA7	0.033142436	0.110542274	cellular_component
GO:0005876	spindle microtubule	1	25	20	14857	0.04	0.001346167	29.714	332	BIRC5	0.033142436	0.110542274	cellular_component
GO:0030593	neutrophil chemotaxis	1	25	18	13332	0.04	0.001350135	29.62666667	5294	PIK3CG	0.033241582	0.053098279	biological_process
GO:0019731	antibacterial humoral response	1	25	18	13332	0.04	0.001350135	29.62666667	6590	SLPI	0.033241582	0.053098279	biological_process

GO:0060079	excitatory postsynaptic potential	1	25	18	13332	0.04	0.001350135	29.62666667	1813	DRD2	0.033241582	0.053098279	biological_process
GO:0007194	negative regulation of adenylate cyclase activity	1	25	18	13332	0.04	0.001350135	29.62666667	1813	DRD2	0.033241582	0.053098279	biological_process
GO:0007628	adult walking behavior	1	25	18	13332	0.04	0.001350135	29.62666667	1813	DRD2	0.033241582	0.053098279	biological_process
GO:0046856	phosphatidylinositol dephosphorylation	1	25	18	13332	0.04	0.001350135	29.62666667	5728	PTEN	0.033241582	0.053098279	biological_process
GO:2000463	ositive regulation of excitatory postsynaptic potentia	1	25	18	13332	0.04	0.001350135	29.62666667	1139	CHRNA7	0.033241582	0.053098279	biological_process
GO:0048678	response to axon injury	1	25	18	13332	0.04	0.001350135	29.62666667	1813	DRD2	0.033241582	0.053098279	biological_process
GO:0030054	cell junction	3	25	429	14857	0.12	0.028875278	4.155804196	91;2697;11	;GJA1;CHI	0.034341636	0.110542274	cellular_component
GO:0045070	positive regulation of viral genome replication	1	25	19	13332	0.04	0.001425143	28.06736842	7153	TOP2A	0.035056886	0.055506737	biological_process
GO:0045776	negative regulation of blood pressure	1	25	19	13332	0.04	0.001425143	28.06736842	1813	DRD2	0.035056886	0.055506737	biological_process
GO:0048018	receptor ligand activity	1	25	18	12391	0.04	0.001452667	27.53555556	7422	VEGFA	0.035724612	0.069247943	molecular_function
GO:0005769	early endosome	2	25	179	14857	0.08	0.012048193	6.64	3791;2697	KDR;GJA1	0.036119894	0.110542274	cellular_component
GO:0003677	DNA binding	6	25	1264	12391	0.24	0.102009523	2.352721519	);196;7153;	;AHR;TOP	0.036212227	0.069247943	molecular_function
GO:0002102	podosome	1	25	22	14857	0.04	0.001480783	27.01272727	5599	MAPK8	0.036398066	0.110542274	cellular_component
GO:0030687	preribosome, large subunit precursor	1	25	22	14857	0.04	0.001480783	27.01272727	3692	EIF6	0.036398066	0.110542274	cellular_component
GO:0040018	positive regulation of multicellular organism growth	1	25	20	13332	0.04	0.00150015	26.664	1813	DRD2	0.036868918	0.056636934	biological_process
GO:0007616	long-term memory	1	25	20	13332	0.04	0.00150015	26.664	1813	DRD2	0.036868918	0.056636934	biological_process
GO:0046676	negative regulation of insulin secretion	1	25	20	13332	0.04	0.00150015	26.664	1813	DRD2	0.036868918	0.056636934	biological_process
GO:0003091	renal water homeostasis	1	25	20	13332	0.04	0.00150015	26.664	231	AKR1B1	0.036868918	0.056636934	biological_process
GO:0001890	placenta development	1	25	20	13332	0.04	0.00150015	26.664	1432	MAPK14	0.036868918	0.056636934	biological_process
GO:0051482	on concentration involved in phospholipase C-activat	1	25	20	13332	0.04	0.00150015	26.664	1813	DRD2	0.036868918	0.056636934	biological_process
GO:0048661	ositive regulation of smooth muscle cell proliferatio	1	25	20	13332	0.04	0.00150015	26.664	231	AKR1B1	0.036868918	0.056636934	biological_process
GO:0035255	ionotropic glutamate receptor binding	1	25	19	12391	0.04	0.001533371	26.08631579	1813	DRD2	0.037672958	0.070799181	molecular_function
GO:0007608	sensory perception of smell	1	25	21	13332	0.04	0.001575158	25.39428571	1813	DRD2	0.038677683	0.05793628	biological_process
GO:0002931	response to ischemia	1	25	21	13332	0.04	0.001575158	25.39428571	2697	GJA1	0.038677683	0.05793628	biological_process
GO:0071479	cellular response to ionizing radiation	1	25	21	13332	0.04	0.001575158	25.39428571	1432	MAPK14	0.038677683	0.05793628	biological_process
GO:0032720	egative regulation of tumor necrosis factor productio	1	25	21	13332	0.04	0.001575158	25.39428571	1139	CHRNA7	0.038677683	0.05793628	biological_process
GO:0009411	response to UV	1	25	21	13332	0.04	0.001575158	25.39428571	5599	MAPK8	0.038677683	0.05793628	biological_process
GO:0002027	regulation of heart rate	1	25	21	13332	0.04	0.001575158	25.39428571	1813	DRD2	0.038677683	0.05793628	biological_process
GO:0046875	ephrin receptor binding	1	25	20	12391	0.04	0.001614075	24.782	5294	PIK3CG	0.039617525	0.071902488	molecular_function
GO:0001205	activity, RNA polymerase II distal enhancer sequenc	1	25	20	12391	0.04	0.001614075	24.782	4780	NFE2L2	0.039617525	0.071902488	molecular_function
GO:0000287	magnesium ion binding	2	25	159	12391	0.08	0.012831894	6.234465409	7153;5728	OP2A;PTE	0.040476668	0.071902488	molecular_function
GO:0042110	T cell activation	1	25	22	13332	0.04	0.001650165	24.24	5294	PIK3CG	0.040483186	0.059895206	biological_process
GO:0050808	synapse organization	1	25	22	13332	0.04	0.001650165	24.24	1139	CHRNA7	0.040483186	0.059895206	biological_process
GO:0018108	peptidyl-tyrosine phosphorylation	1	25	22	13332	0.04	0.001650165	24.24	3791	KDR	0.040483186	0.059895206	biological_process
GO:0032993	protein-DNA complex	1	25	25	14857	0.04	0.001682708	23.7712	4780	NFE2L2	0.041261785	0.120838085	cellular_component
GO:0006955	immune response	2	25	173	13332	0.08	0.012976298	6.165086705	6590;3565	SLPI;IL4	0.041316898	0.060879184	biological_process
GO:0016303	1-phosphatidylinositol-3-kinase activity	1	25	21	12391	0.04	0.001694778	23.60190476	5294	PIK3CG	0.041558319	0.071902488	molecular_function
GO:0017025	TBP-class protein binding	1	25	21	12391	0.04	0.001694778	23.60190476	196	AHR	0.041558319	0.071902488	molecular_function
GO:0032870	cellular response to hormone stimulus	1	25	23	13332	0.04	0.001725173	23.18608696	10499	NCOA2	0.042285434	0.061552588	biological_process
GO:0002088	lens development in camera-type eye	1	25	23	13332	0.04	0.001725173	23.18608696	2697	GJA1	0.042285434	0.061552588	biological_process
GO:0034097	response to cytokine	1	25	23	13332	0.04	0.001725173	23.18608696	9536	PTGES	0.042285434	0.061552588	biological_process
GO:0005615	extracellular space	4	25	802	14857	0.16	0.053981288	2.963990025	7422;6590;	1;VEGFA;	0.043174955	0.122080906	cellular_component
GO:0046966	thyroid hormone receptor binding	1	25	22	12391	0.04	0.001775482	22.52909091	6256	RXRA	0.043495347	0.072938352	molecular_function
GO:0048487	beta-tubulin binding	1	25	22	12391	0.04	0.001775482	22.52909091	2697	GJA1	0.043495347	0.072938352	molecular_function
GO:0002062	chondrocyte differentiation	1	25	24	13332	0.04	0.00180018	22.22	1432	MAPK14	0.044084432	0.063404303	biological_process
GO:0001568	blood vessel development	1	25	24	13332	0.04	0.00180018	22.22	196	AHR	0.044084432	0.063404303	biological_process
GO:0070527	platelet aggregation	1	25	24	13332	0.04	0.00180018	22.22	5294	PIK3CG	0.044084432	0.063404303	biological_process
GO:0007186	G-protein coupled receptor signaling pathway	3	25	427	13332	0.12	0.032028203	3.746697892	33;1813;52	3;DRD2;P	0.044530058	0.063791076	biological_process
GO:0030424	axon	2	25	203	14857	0.08	0.013663593	5.854975369	1813;5599	RD2;MAPI	0.045365574	0.123999235	cellular_component
GO:0001102	JA polymerase II activating transcription factor bindi	1	25	23	12391	0.04	0.001856186	21.54956522	4780	NFE2L2	0.045428617	0.07502605	molecular_function

GO:0008219	cell death	1	25	25	13332	0.04	0.001875188	21.3312	5733	PTGER3	0.045880186	0.065465404	biological_process
GO:0006954	inflammatory response	2	25	185	13332	0.08	0.013876388	5.765189189	5294;4780	3CG;NFE2	0.046627648	0.066270003	biological_process
GO:0004866	endopeptidase inhibitor activity	1	25	24	12391	0.04	0.00193689	20.65166667	6590	SLPI	0.047358136	0.076335937	molecular_function
GO:0019904	protein domain specific binding	2	25	174	12391	0.08	0.01404245	5.697011494	10499;478(	OA2;NFE2	0.047622419	0.076335937	molecular_function
GO:0000910	cytokinesis	1	25	26	13332	0.04	0.001950195	20.51076923	332	BIRC5	0.047672701	0.066964377	biological_process
GO:0014068	tive regulation of phosphatidylinositol 3-kinase signa	1	25	26	13332	0.04	0.001950195	20.51076923	3791	KDR	0.047672701	0.066964377	biological_process
GO:0032467	positive regulation of cytokinesis	1	25	26	13332	0.04	0.001950195	20.51076923	1813	DRD2	0.047672701	0.066964377	biological_process
GO:0036126	sperm flagellum	1	25	29	14857	0.04	0.001951942	20.49241379	1813	DRD2	0.047710096	0.126200899	cellular_component
GO:0001046	core promoter sequence-specific DNA binding	1	25	25	12391	0.04	0.002017593	19.8256	5468	PPARG	0.04928391	0.076742088	molecular_function
GO:0015276	ligand-gated ion channel activity	1	25	25	12391	0.04	0.002017593	19.8256	1139	CHRNA7	0.04928391	0.076742088	molecular_function
GO:0030139	endocytic vesicle	1	25	30	14857	0.04	0.00201925	19.80933333	1813	DRD2	0.049315656	0.126371369	cellular_component
GO:0051781	positive regulation of cell division	1	25	27	13332	0.04	0.002025203	19.75111111	7422	VEGFA	0.049461983	0.068151816	biological_process
GO:0051592	response to calcium ion	1	25	27	13332	0.04	0.002025203	19.75111111	9536	PTGES	0.049461983	0.068151816	biological_process
GO:0045785	positive regulation of cell adhesion	1	25	27	13332	0.04	0.002025203	19.75111111	7422	VEGFA	0.049461983	0.068151816	biological_process
GO:0007631	feeding behavior	1	25	27	13332	0.04	0.002025203	19.75111111	1813	DRD2	0.049461983	0.068151816	biological_process
GO:0014070	response to organic cyclic compound	1	25	27	13332	0.04	0.002025203	19.75111111	9536	PTGES	0.049461983	0.068151816	biological_process
GO:0046934	hosphatidylinositol-4,5-bisphosphate 3-kinase activit	1	25	26	12391	0.04	0.002098297	19.06307692	5294	PIK3CG	0.051205946	0.077520113	molecular_function
GO:0070888	E-box binding	1	25	26	12391	0.04	0.002098297	19.06307692	196	AHR	0.051205946	0.077520113	molecular_function
GO:0032024	positive regulation of insulin secretion	1	25	28	13332	0.04	0.00210021	19.04571429	2697	GJA1	0.051248037	0.070344263	biological_process
GO:0006366	transcription from RNA polymerase II promoter	2	25	198	13332	0.08	0.014851485	5.386666667	196;4780	HR;NFE2I	0.052650521	0.071995599	biological_process
GO:0008542	visual learning	1	25	29	13332	0.04	0.002175218	18.38896552	1813	DRD2	0.053030869	0.072242052	biological_process
GO:0005881	cytoplasmic microtubule	1	25	33	14857	0.04	0.002221175	18.00848485	332	BIRC5	0.05411676	0.134471949	cellular_component
GO:0006110	regulation of glycolytic process	1	25	30	13332	0.04	0.002250225	17.776	3692	EIF6	0.054810485	0.073013229	biological_process
GO:0042149	cellular response to glucose starvation	1	25	30	13332	0.04	0.002250225	17.776	4780	NFE2L2	0.054810485	0.073013229	biological_process
GO:0007611	learning or memory	1	25	30	13332	0.04	0.002250225	17.776	1139	CHRNA7	0.054810485	0.073013229	biological_process
GO:0045600	positive regulation of fat cell differentiation	1	25	30	13332	0.04	0.002250225	17.776	5468	PPARG	0.054810485	0.073013229	biological_process
GO:0071320	cellular response to cAMP	1	25	30	13332	0.04	0.002250225	17.776	5294	PIK3CG	0.054810485	0.073013229	biological_process
GO:0009612	response to mechanical stimulus	1	25	30	13332	0.04	0.002250225	17.776	5599	MAPK8	0.054810485	0.073013229	biological_process
GO:0043021	ribonucleoprotein complex binding	1	25	28	12391	0.04	0.002259705	17.70142857	7155	TOP2B	0.055038834	0.082181273	molecular_function
GO:0048813	dendrite morphogenesis	1	25	31	13332	0.04	0.002325233	17.20258065	5599	MAPK8	0.05658689	0.074283153	biological_process
GO:0038083	peptidyl-tyrosine autophosphorylation	1	25	31	13332	0.04	0.002325233	17.20258065	3791	KDR	0.05658689	0.074283153	biological_process
GO:0031098	stress-activated protein kinase signaling cascade	1	25	31	13332	0.04	0.002325233	17.20258065	231	AKR1B1	0.05658689	0.074283153	biological_process
GO:0030900	forebrain development	1	25	31	13332	0.04	0.002325233	17.20258065	7155	TOP2B	0.05658689	0.074283153	biological_process
GO:0050890	cognition	1	25	32	13332	0.04	0.00240024	16.665	1139	CHRNA7	0.058360089	0.076057733	biological_process
GO:0006950	response to stress	1	25	32	13332	0.04	0.00240024	16.665	231	AKR1B1	0.058360089	0.076057733	biological_process
GO:0060170	ciliary membrane	1	25	36	14857	0.04	0.0024231	16.50777778	1813	DRD2	0.058894582	0.141700768	cellular_component
GO:0045071	negative regulation of viral genome replication	1	25	33	13332	0.04	0.002475248	16.16	6590	SLPI	0.060130089	0.077802732	biological_process
GO:0034198	cellular response to amino acid starvation	1	25	33	13332	0.04	0.002475248	16.16	5599	MAPK8	0.060130089	0.077802732	biological_process
GO:0014704	intercalated disc	1	25	37	14857	0.04	0.002490409	16.06162162	2697	GJA1	0.060482035	0.141700768	cellular_component
GO:0001085	RNA polymerase II transcription factor binding	1	25	31	12391	0.04	0.002501816	15.9883871	2002	ELK1	0.060760308	0.089498291	molecular_function
GO:0045669	positive regulation of osteoblast differentiation	1	25	34	13332	0.04	0.002550255	15.68470588	2697	GJA1	0.061896895	0.079802782	biological_process
GO:0008138	protein tyrosine/serine/threonine phosphatase activity	1	25	32	12391	0.04	0.00258252	15.48875	5728	PTEN	0.062660064	0.09106596	molecular_function
GO:0001570	vasculogenesis	1	25	35	13332	0.04	0.002625263	15.23657143	3791	KDR	0.063660511	0.08092058	biological_process
GO:0006006	glucose metabolic process	1	25	35	13332	0.04	0.002625263	15.23657143	1432	MAPK14	0.063660511	0.08092058	biological_process
GO:0032868	response to insulin	1	25	35	13332	0.04	0.002625263	15.23657143	3692	EIF6	0.063660511	0.08092058	biological_process
GO:0071260	cellular response to mechanical stimulus	1	25	35	13332	0.04	0.002625263	15.23657143	2697	GJA1	0.063660511	0.08092058	biological_process
GO:0015459	potassium channel regulator activity	1	25	33	12391	0.04	0.002663223	15.01939394	1813	DRD2	0.064556132	0.091384654	molecular_function
GO:0000976	cription regulatory region sequence-specific DNA bi	1	25	33	12391	0.04	0.002663223	15.01939394	4780	NFE2L2	0.064556132	0.091384654	molecular_function
GO:0035580	specific granule lumen	1	25	40	14857	0.04	0.002692334	14.857	6590	SLPI	0.065228984	0.148577131	cellular_component
GO:0030968	endoplasmic reticulum unfolded protein response	1	25	36	13332	0.04	0.00270027	14.81333333	4780	NFE2L2	0.065420945	0.082003338	biological_process

GO:0007254	JNK cascade	1	25	36	13332	0.04	0.00270027	14.81333333	5599	MAPK8	0.065420945	0.082003338	biological_process
GO:0033138	ositive regulation of peptidyl-serine phosphorylatio	1	25	36	13332	0.04	0.00270027	14.81333333	7422	VEGFA	0.065420945	0.082003338	biological_process
GO:0007519	skeletal muscle tissue development	1	25	36	13332	0.04	0.00270027	14.81333333	1432	MAPK14	0.065420945	0.082003338	biological_process
GO:0000980	olymerase II distal enhancer sequence-specific DNA	1	25	34	12391	0.04	0.002743927	14.57764706	4780	NFE2L2	0.066448517	0.092857543	molecular_function
GO:0007613	memory	1	25	37	13332	0.04	0.002775278	14.41297297	1139	CHRNA7	0.067178201	0.083337906	biological_process
GO:0043154	n of cysteine-type endopeptidase activity involved in	1	25	37	13332	0.04	0.002775278	14.41297297	332	BIRC5	0.067178201	0.083337906	biological_process
GO:0009749	response to glucose	1	25	37	13332	0.04	0.002775278	14.41297297	2697	GJA1	0.067178201	0.083337906	biological_process
GO:0045727	positive regulation of translation	1	25	38	13332	0.04	0.002850285	14.03368421	3692	EIF6	0.068932285	0.084930222	biological_process
GO:0048812	neuron projection morphogenesis	1	25	38	13332	0.04	0.002850285	14.03368421	2697	GJA1	0.068932285	0.084930222	biological_process
GO:0042733	embryonic digit morphogenesis	1	25	39	13332	0.04	0.002925293	13.67384615	2697	GJA1	0.070683202	0.086204851	biological_process
GO:0045652	regulation of megakaryocyte differentiation	1	25	39	13332	0.04	0.002925293	13.67384615	3692	EIF6	0.070683202	0.086204851	biological_process
GO:0032880	regulation of protein localization	1	25	39	13332	0.04	0.002925293	13.67384615	5599	MAPK8	0.070683202	0.086204851	biological_process
GO:0030425	dendrite	2	25	261	14857	0.08	0.017567477	4.553869732	1813;2002	RD2;ELK	0.070792221	0.153040498	cellular_component
GO:0006468	protein phosphorylation	2	25	235	13332	0.08	0.017626763	4.538553191	332;5599	RC5;MAP	0.07119272	0.086533912	biological_process
GO:0030145	manganese ion binding	1	25	37	12391	0.04	0.002986038	13.39567568	6648	SOD2	0.072103648	0.09948478	molecular_function
GO:0002244	hematopoietic progenitor cell differentiation	1	25	40	13332	0.04	0.0030003	13.332	7153	TOP2A	0.072430958	0.087743543	biological_process
GO:0015631	tubulin binding	1	25	38	12391	0.04	0.003066742	13.04315789	332	BIRC5	0.073981372	0.10079962	molecular_function
GO:0000902	cell morphogenesis	1	25	41	13332	0.04	0.003075308	13.00682927	1432	MAPK14	0.074175558	0.089257922	biological_process
GO:0050731	ositive regulation of peptidyl-tyrosine phosphorylatio	1	25	41	13332	0.04	0.003075308	13.00682927	7422	VEGFA	0.074175558	0.089257922	biological_process
GO:0005829	cytosol	10	25	3743	14857	0.4	0.251935115	1.587710393	;3692;559	EIF6;MA	0.074432014	0.153040498	cellular_component
GO:0030141	secretory granule	1	25	46	14857	0.04	0.003096184	12.91913043	7422	VEGFA	0.074653901	0.153040498	cellular_component
GO:0000775	chromosome, centromeric region	1	25	46	14857	0.04	0.003096184	12.91913043	332	BIRC5	0.074653901	0.153040498	cellular_component
GO:0001540	amyloid-beta binding	1	25	39	12391	0.04	0.003147446	12.70871795	1139	CHRNA7	0.075855449	0.100832243	molecular_function
GO:0097110	scaffold protein binding	1	25	39	12391	0.04	0.003147446	12.70871795	2697	GJA1	0.075855449	0.100832243	molecular_function
GO:0007200	pase C-activating G-protein coupled receptor signalin	1	25	42	13332	0.04	0.003150315	12.69714286	5733	PTGER3	0.075917008	0.090748477	biological_process
GO:0006805	xenobiotic metabolic process	1	25	42	13332	0.04	0.003150315	12.69714286	196	AHR	0.075917008	0.090748477	biological_process
GO:0001947	heart looping	1	25	43	13332	0.04	0.003225323	12.40186047	2697	GJA1	0.077655313	0.092520027	biological_process
GO:0003743	translation initiation factor activity	1	25	40	12391	0.04	0.003228149	12.391	3692	EIF6	0.077725884	0.102073751	molecular_function
GO:0043410	positive regulation of MAPK cascade	1	25	44	13332	0.04	0.00330033	12.12	3791	KDR	0.079390479	0.094276194	biological_process
GO:0004722	protein serine/threonine phosphatase activity	1	25	43	12391	0.04	0.003470261	11.52651163	5728	PTEN	0.083315409	0.107949729	molecular_function
GO:0051259	protein oligomerization	1	25	47	13332	0.04	0.003525353	11.34638298	2697	GJA1	0.084577194	0.099778978	biological_process
GO:0031647	regulation of protein stability	1	25	47	13332	0.04	0.003525353	11.34638298	5728	PTEN	0.084577194	0.099778978	biological_process
GO:0019903	protein phosphatase binding	1	25	44	12391	0.04	0.003550964	11.26454545	1432	MAPK14	0.085171346	0.107949729	molecular_function
GO:0043022	ribosome binding	1	25	44	12391	0.04	0.003550964	11.26454545	3692	EIF6	0.085171346	0.107949729	molecular_function
GO:0005887	integral component of plasma membrane	4	25	1018	14857	0.16	0.06851989	2.335088409	2697;5733	A1;PTGE	0.087932722	0.174863086	cellular_component
GO:0006816	calcium ion transport	1	25	49	13332	0.04	0.003675368	10.88326531	1139	CHRNA7	0.088019407	0.103165603	biological_process
GO:0018107	peptidyl-threonine phosphorylation	1	25	49	13332	0.04	0.003675368	10.88326531	5599	MAPK8	0.088019407	0.103165603	biological_process
GO:0034599	cellular response to oxidative stress	1	25	50	13332	0.04	0.003750375	10.6656	4780	NFE2L2	0.08973585	0.104837029	biological_process
GO:0005739	mitochondrion	4	25	1031	14857	0.16	0.069394898	2.305645005	2697;5599	GJA1;MA	0.091170159	0.174863086	cellular_component
GO:0001503	ossification	1	25	51	13332	0.04	0.003825383	10.45647059	5599	MAPK8	0.091449192	0.105811404	biological_process
GO:0045732	positive regulation of protein catabolic process	1	25	51	13332	0.04	0.003825383	10.45647059	2697	GJA1	0.091449192	0.105811404	biological_process
GO:0007626	locomotory behavior	1	25	51	13332	0.04	0.003825383	10.45647059	1813	DRD2	0.091449192	0.105811404	biological_process
GO:0030672	synaptic vesicle membrane	1	25	57	14857	0.04	0.003836575	10.42596491	1813	DRD2	0.091696497	0.174863086	cellular_component
GO:0000978	ymerase II proximal promoter sequence-specific DN	2	25	254	12391	0.08	0.020498749	3.902677165	10499;200	COA2;EL	0.092273357	0.115606849	molecular_function
GO:0045454	cell redox homeostasis	1	25	52	13332	0.04	0.00390039	10.25538462	4780	NFE2L2	0.093159437	0.107103684	biological_process
GO:0008104	protein localization	1	25	52	13332	0.04	0.00390039	10.25538462	1813	DRD2	0.093159437	0.107103684	biological_process
GO:0009055	electron transfer activity	1	25	49	12391	0.04	0.003954483	10.11510204	231	AKR1B1	0.094397054	0.116923623	molecular_function
GO:0000785	chromatin	1	25	59	14857	0.04	0.003971192	10.07254237	4780	NFE2L2	0.094762601	0.17660303	cellular_component
GO:0007416	synapse assembly	1	25	53	13332	0.04	0.003975398	10.06188679	1813	DRD2	0.094866592	0.108720126	biological_process
GO:0005262	calcium channel activity	1	25	51	12391	0.04	0.004115891	9.718431373	1139	CHRNA7	0.098062272	0.120098737	molecular_function

GO:0030168	platelet activation	1	25	56	13332	0.04	0.00420042	9.522857143	5294	PIK3CG	0.099969564	0.114205737	biological_process
GO:0043130	ubiquitin binding	1	25	53	12391	0.04	0.004277298	9.351698113	7153	TOP2A	0.101713246	0.123186043	molecular_function
GO:0016605	PML body	1	25	64	14857	0.04	0.004307734	9.285625	5728	PTEN	0.102384454	0.186567228	cellular_component
GO:0007169	embrane receptor protein tyrosine kinase signaling pa	1	25	59	13332	0.04	0.004425443	9.038644068	3791	KDR	0.105044912	0.119625278	biological_process
GO:0071222	cellular response to lipopolysaccharide	1	25	60	13332	0.04	0.00450045	8.888	5599	MAPK8	0.106730581	0.120782883	biological_process
GO:0019216	regulation of lipid metabolic process	1	25	60	13332	0.04	0.00450045	8.888	10499	NCOA2	0.106730581	0.120782883	biological_process
GO:0005794	Golgi apparatus	3	25	688	14857	0.12	0.046308138	2.591337209	91;2697;47	;GJA1;NF	0.107011862	0.190760275	cellular_component
GO:0004674	protein serine/threonine kinase activity	2	25	281	12391	0.08	0.02267775	3.527686833	5294;5599	3CG;MAF	0.109362111	0.130994177	molecular_function
GO:0045087	innate immune response	2	25	303	13332	0.08	0.022727273	3.52	5294;6590	IK3CG;SL	0.109770587	0.123834943	biological_process
GO:0007165	signal transduction	3	25	628	13332	0.12	0.04710471	2.547515924	39;2697;95	A7;GJA1;	0.111181097	0.124918649	biological_process
GO:0007568	aging	1	25	63	13332	0.04	0.004725473	8.464761905	4780	NFE2L2	0.111769318	0.124918649	biological_process
GO:0000187	activation of MAPK activity	1	25	63	13332	0.04	0.004725473	8.464761905	1139	CHRNA7	0.111769318	0.124918649	biological_process
GO:0035556	intracellular signal transduction	2	25	308	13332	0.08	0.02310231	3.462857143	1432;1813	APK14;DR	0.112797695	0.125678914	biological_process
GO:0006661	phosphatidylinositol biosynthetic process	1	25	64	13332	0.04	0.00480048	8.3325	5294	PIK3CG	0.113442825	0.126008799	biological_process
GO:0005882	intermediate filament	1	25	72	14857	0.04	0.0048462	8.253888889	2697	GJA1	0.114451433	0.199681223	cellular_component
GO:0004713	protein tyrosine kinase activity	1	25	62	12391	0.04	0.005003632	7.994193548	3791	KDR	0.117967793	0.139766189	molecular_function
GO:0001649	osteoblast differentiation	1	25	67	13332	0.04	0.005025503	7.959402985	2697	GJA1	0.118445205	0.131161714	biological_process
GO:0051897	positive regulation of protein kinase B signaling	1	25	68	13332	0.04	0.00510051	7.842352941	5294	PIK3CG	0.120106635	0.132594786	biological_process
GO:0001669	acrosomal vesicle	1	25	79	14857	0.04	0.005317359	7.522531646	1813	DRD2	0.124882154	0.213340347	cellular_component
GO:0006470	protein dephosphorylation	1	25	71	13332	0.04	0.005325533	7.510985915	5728	PTEN	0.125072908	0.137656463	biological_process
GO:0005178	integrin binding	1	25	67	12391	0.04	0.00540715	7.39761194	3791	KDR	0.126875696	0.148703773	molecular_function
GO:0030308	negative regulation of cell growth	1	25	73	13332	0.04	0.005475548	7.305205479	2697	GJA1	0.128368796	0.140854515	biological_process
GO:0043197	dendritic spine	1	25	83	14857	0.04	0.005586592	7.16	1813	DRD2	0.130789504	0.216906972	cellular_component
GO:0008270	zinc ion binding	3	25	629	12391	0.12	0.05076265	2.363942766	32;5468;625	;PPARG;	0.131211306	0.15214928	molecular_function
GO:0010976	positive regulation of neuron projection development	1	25	75	13332	0.04	0.005625563	7.1104	4780	NFE2L2	0.131652763	0.144020143	biological_process
GO:0000777	condensed chromosome kinetochore	1	25	84	14857	0.04	0.005653901	7.074761905	332	BIRC5	0.132260349	0.216906972	cellular_component
GO:0004725	protein tyrosine phosphatase activity	1	25	72	12391	0.04	0.005810669	6.883888889	5728	PTEN	0.1356972	0.155694682	molecular_function
GO:0007417	central nervous system development	1	25	79	13332	0.04	0.005925593	6.750379747	5728	PTEN	0.138185098	0.149804266	biological_process
GO:0006936	muscle contraction	1	25	79	13332	0.04	0.005925593	6.750379747	2697	GJA1	0.138185098	0.149804266	biological_process
GO:0005975	carbohydrate metabolic process	1	25	79	13332	0.04	0.005925593	6.750379747	231	AKR1B1	0.138185098	0.149804266	biological_process
GO:0004867	serine-type endopeptidase inhibitor activity	1	25	74	12391	0.04	0.005972077	6.697837838	6590	SLPI	0.13920179	0.158052032	molecular_function
GO:0043204	perikaryon	1	25	89	14857	0.04	0.005990442	6.677303371	1813	DRD2	0.139578805	0.224420823	cellular_component
GO:0000922	spindle pole	1	25	91	14857	0.04	0.006125059	6.530549451	1432	MAPK14	0.142489564	0.224695082	cellular_component
GO:0045892	negative regulation of transcription, DNA-templated	2	25	358	13332	0.08	0.026852685	2.979217877	332;196	BIRC5;AHI	0.144206341	0.15586374	biological_process
GO:0000086	G2/M transition of mitotic cell cycle	1	25	83	13332	0.04	0.006225623	6.425060241	332	BIRC5	0.144670245	0.155898383	biological_process
GO:0006811	ion transport	1	25	85	13332	0.04	0.006375638	6.273882353	1139	CHRNA7	0.147895224	0.158899333	biological_process
GO:0006351	transcription, DNA-templated	5	25	1565	13332	0.2	0.117386739	1.703769968	2;10499;54	5;NCOA2	0.162229712	0.173783163	biological_process
GO:0005814	centriole	1	25	105	14857	0.04	0.007067376	5.659809524	332	BIRC5	0.162601803	0.251572601	cellular_component
GO:0030335	positive regulation of cell migration	1	25	95	13332	0.04	0.007125713	5.613473684	3791	KDR	0.163845803	0.174995074	biological_process
GO:0017124	SH3 domain binding	1	25	89	12391	0.04	0.007182633	5.568988764	2697	GJA1	0.165054801	0.185473951	molecular_function
GO:0002250	adaptive immune response	1	25	96	13332	0.04	0.00720072	5.555	5294	PIK3CG	0.165425002	0.176160548	biological_process
GO:0042493	response to drug	1	25	98	13332	0.04	0.007350735	5.441632653	1813	DRD2	0.168574814	0.178986788	biological_process
GO:0043123	itive regulation of I-kappaB kinase/NF-kappaB signa	1	25	99	13332	0.04	0.007425743	5.386666667	2697	GJA1	0.170145438	0.180124642	biological_process
GO:0003729	mRNA binding	1	25	93	12391	0.04	0.007505448	5.329462366	6590	SLPI	0.171822031	0.191108178	molecular_function
GO:0008201	heparin binding	1	25	95	12391	0.04	0.007666855	5.217263158	7422	VEGFA	0.17518587	0.192881413	molecular_function
GO:0048471	perinuclear region of cytoplasm	2	25	454	14857	0.08	0.030557986	2.617973568	231;9536	R1B1;PTC	0.176879492	0.268176505	cellular_component
GO:0090090	egative regulation of canonical Wnt signaling pathwa	1	25	105	13332	0.04	0.007875788	5.078857143	1432	MAPK14	0.179509531	0.189482283	biological_process
GO:0042802	identical protein binding	3	25	739	12391	0.12	0.059640061	2.012070365	91;5294;18	;PIK3CG;I	0.18467178	0.20129224	molecular_function
GO:0005635	nuclear envelope	1	25	121	14857	0.04	0.008144309	4.911404959	5733	PTGER3	0.185032763	0.268176505	cellular_component
GO:0030496	midbody	1	25	121	14857	0.04	0.008144309	4.911404959	332	BIRC5	0.185032763	0.268176505	cellular_component

GO:0005741	mitochondrial outer membrane	1	25	122	14857	0.04	0.008211617	4.871147541	2697	GJA1	0.186415375	0.268176505	cellular_component
GO:0001701	in utero embryonic development	1	25	110	13332	0.04	0.008250825	4.848	2697	GJA1	0.187235375	0.197061138	biological_process
GO:0006897	endocytosis	1	25	111	13332	0.04	0.008325833	4.804324324	5294	PIK3CG	0.188772141	0.198100997	biological_process
GO:0005667	transcription factor complex	1	25	127	14857	0.04	0.008548159	4.679370079	196	AHR	0.19329473	0.269655378	cellular_component
GO:0005886	plasma membrane	7	25	2870	14857	0.28	0.193174934	1.449463415	97;5733;47	PTGER3;N	0.194020333	0.269655378	cellular_component
GO:0046777	protein autophosphorylation	1	25	117	13332	0.04	0.008775878	4.557948718	3791	KDR	0.197934321	0.207113884	biological_process
GO:0006810	transport	1	25	118	13332	0.04	0.008850885	4.519322034	2697	GJA1	0.199451661	0.208098409	biological_process
GO:0016477	cell migration	1	25	125	13332	0.04	0.009375938	4.26624	5728	PTEN	0.209996141	0.218468608	biological_process
GO:0030198	extracellular matrix organization	1	25	135	13332	0.04	0.010126013	3.950222222	3791	KDR	0.224828674	0.233227446	biological_process
GO:0016055	Wnt signaling pathway	1	25	136	13332	0.04	0.01020102	3.921176471	1813	DRD2	0.226297135	0.234078125	biological_process
GO:0005764	lysosome	1	25	157	14857	0.04	0.010567409	3.78522293	2697	GJA1	0.233412876	0.318997597	cellular_component
GO:0006974	cellular response to DNA damage stimulus	1	25	145	13332	0.04	0.010876088	3.677793103	7153	TOP2A	0.23939366	0.246917461	biological_process
GO:0014069	postsynaptic density	1	25	162	14857	0.04	0.010903951	3.668395062	1813	DRD2	0.23991022	0.322502263	cellular_component
GO:0045211	postsynaptic membrane	1	25	172	14857	0.04	0.011577034	3.455116279	1139	CHRNA7	0.252746668	0.328971854	cellular_component
GO:0043005	neuron projection	1	25	172	14857	0.04	0.011577034	3.455116279	5599	MAPK8	0.252746668	0.328971854	cellular_component
GO:0004871	signal transducer activity	1	25	147	12391	0.04	0.011863449	3.37170068	2697	GJA1	0.258181445	0.27863146	molecular_function
GO:0043161	ome-mediated ubiquitin-dependent protein catabolic	1	25	161	13332	0.04	0.012076208	3.312298137	4780	NFE2L2	0.262152665	0.269621402	biological_process
GO:0007267	cell-cell signaling	1	25	168	13332	0.04	0.01260126	3.174285714	2697	GJA1	0.27190283	0.278854891	biological_process
GO:0043065	positive regulation of apoptotic process	1	25	173	13332	0.04	0.012976298	3.082543353	7153	TOP2A	0.278791378	0.285109596	biological_process
GO:0003713	transcription coactivator activity	1	25	162	12391	0.04	0.013074005	3.059506173	10499	NCOA2	0.280592064	0.299848382	molecular_function
GO:0005768	endosome	1	25	195	14857	0.04	0.013125126	3.047589744	3791	KDR	0.281485504	0.360653302	cellular_component
GO:0006355	regulation of transcription, DNA-templated	3	25	1017	13332	0.12	0.076282628	1.573097345	68;196;104	G;AHR;N	0.296978003	0.302850449	biological_process
GO:0016324	apical plasma membrane	1	25	210	14857	0.04	0.014134751	2.829904762	2697	GJA1	0.299653304	0.377503811	cellular_component
GO:0008017	microtubule binding	1	25	177	12391	0.04	0.014284561	2.800225989	332	BIRC5	0.302351943	0.316888094	molecular_function
GO:0001077	ctivity, RNA polymerase II proximal promoter seque	1	25	177	12391	0.04	0.014284561	2.800225989	2002	ELK1	0.302351943	0.316888094	molecular_function
GO:0005730	nucleolus	2	25	657	14857	0.08	0.044221579	1.809071537	3692;7153	IF6;TOP2	0.30384453	0.377503811	cellular_component
GO:0016604	nuclear body	1	25	220	14857	0.04	0.014807835	2.701272727	10499	NCOA2	0.311519282	0.381262405	cellular_component
GO:0008134	transcription factor binding	1	25	185	12391	0.04	0.014930191	2.679135135	196	AHR	0.313697411	0.325647788	molecular_function
GO:0043025	neuronal cell body	1	25	229	14857	0.04	0.01541361	2.59510917	2002	ELK1	0.322033445	0.388334449	cellular_component
GO:0005789	endoplasmic reticulum membrane	2	25	703	14857	0.08	0.047317763	1.690697013	2697;9536	JA1;PTGE	0.332807371	0.395510209	cellular_component
GO:0016032	viral process	1	25	217	13332	0.04	0.016276628	2.457511521	3791	KDR	0.336772002	0.342463923	biological_process
GO:0007049	cell cycle	1	25	230	13332	0.04	0.017251725	2.318608696	196	AHR	0.353027998	0.357986257	biological_process
GO:0005925	focal adhesion	1	25	264	14857	0.04	0.017769402	2.251060606	2697	GJA1	0.36147537	0.423442577	cellular_component
GO:0005102	receptor binding	1	25	235	12391	0.04	0.018965378	2.109106383	2697	GJA1	0.380694669	0.391469047	molecular_function
GO:0016607	nuclear speck	1	25	306	14857	0.04	0.020596352	1.942091503	1432	MAPK14	0.405901698	0.465758635	cellular_component
GO:0005759	mitochondrial matrix	1	25	309	14857	0.04	0.020798277	1.923236246	6648	SOD2	0.408958802	0.465758635	cellular_component
GO:0051301	cell division	1	25	279	13332	0.04	0.020927093	1.911397849	332	BIRC5	0.410930302	0.415534563	biological_process
GO:0016567	protein ubiquitination	1	25	289	13332	0.04	0.021677168	1.845259516	4780	NFE2L2	0.42211965	0.425656965	biological_process
GO:0070062	extracellular exosome	4	25	1967	14857	0.16	0.132395504	1.208500254	3692;2697;1	;EIF6;GJ	0.426569087	0.479159796	cellular_component
GO:0043312	neutrophil degranulation	1	25	314	13332	0.04	0.023552355	1.698343949	6590	SLPI	0.449207468	0.451710017	biological_process
GO:0005813	centrosome	1	25	350	14857	0.04	0.023557919	1.697942857	4780	NFE2L2	0.44925523	0.497823363	cellular_component
GO:0009986	cell surface	1	25	357	14857	0.04	0.024029077	1.66464986	7422	VEGFA	0.455866054	0.498413552	cellular_component
GO:0043565	sequence-specific DNA binding	1	25	307	12391	0.04	0.024776047	1.614462541	6256	RXRA	0.46624556	0.47496043	molecular_function
GO:0016020	membrane	3	25	1512	14857	0.12	0.101770209	1.179126984	22;5294;95	A;PIK3CG;	0.474986214	0.512485126	cellular_component
GO:0000139	Golgi membrane	1	25	429	14857	0.04	0.028875278	1.385268065	2697	GJA1	0.519587593	0.551462036	cellular_component
GO:0005622	intracellular	1	25	435	14857	0.04	0.029279128	1.36616092	1813	DRD2	0.524561449	0.551462036	cellular_component
GO:0043231	intracellular membrane-bounded organelle	1	25	447	14857	0.04	0.030086828	1.329485459	2697	GJA1	0.534361264	0.554653464	cellular_component
GO:0030154	cell differentiation	1	25	432	13332	0.04	0.03240324	1.234444444	2002	ELK1	0.561436528	0.562996074	biological_process
GO:0005576	extracellular region	2	25	1153	14857	0.08	0.077606515	1.030841284	3791;6590	KDR;SLPI	0.588356832	0.603065753	cellular_component
GO:0000122	regulation of transcription from RNA polymerase II	1	25	476	13332	0.04	0.03570357	1.120336134	10499	NCOA2	0.597374125	0.597374125	biological_process

GO:0005783	endoplasmic reticulum	1	25	712	14857	0.04	0.047923538	0.834662921	3791	KDR	0.707346452	0.716079124	cellular_component
GO:0016021	integral component of membrane	3	25	2780	14857	0.12	0.187117184	0.641309353	39;5733;95	7;PTGER3	0.872631932	0.872631932	cellular_component
GO:0003723	RNA binding	1	25	1196	12391	0.04	0.096521669	0.414414716	7153	TOP2A	0.921146925	0.929676063	molecular_function
GO:0046872	metal ion binding	1	25	2019	12391	0.04	0.162940844	0.245487865	7155	TOP2B	0.988335825	0.988335825	molecular_function
